# Mechanistic insights into manganese oxidation of a soil-borne Mn(II)-oxidizing *Escherichia coli* strain by global proteomic and genetic analyses

**DOI:** 10.1038/s41598-017-01552-3

**Published:** 2017-05-02

**Authors:** Zhiyong Wang, Jieping Wang, Jin Liu, Hong Chen, Mingshun Li, Lin Li

**Affiliations:** 1 State Key Laboratory of Agricultural Microbiology, Huazhong Agricultural University, Wuhan, 430070 China; 2 Agricultural Bio-resources Institute, Fujian Academy of Agricultural Sciences, Fuzhou, 350003 China

## Abstract

An iTRAQ-based comparative and quantitative proteomics analysis of a soil-borne Mn(II)-oxidizing bacterium, *Escherichia coli* MB266, was conducted during the exponential and stationary growth phases. A total of 1850 proteins were identified in 4 samples, of which 373 and 456 proteins were significantly up- or down-regulated in at least one pairwise comparison, respectively. The iTRAQ data indicated that several enzymes involved in fatty acid metabolism (i.e., FabA, FabD and FabZ) and pyruvate metabolism (particularly pyruvate oxidase PoxB) were significantly up-regulated, while those related to the tricarboxylic acid cycle (such as FrdB, FumB and AcnA) and methylcitrate cycle (i.e., PrpC) were inactivated in the presence of 1 mM Mn(II); the amounts of some stress response and signal transduction system-related proteins (i.e., Spy) were remarkably increased, and the cold shock protein CspD was significantly up-regulated during the exponential growth phase. However, all verified heat shock proteins remained unchanged. The reactive oxygen species response and some redox enzymes might also be involved in Mn oxidation processes. The involvement of several cellular proteins in Mn(II) oxidation, including PoxB, Spy and MCO266, was further confirmed by gene disruption and expression complementation experiments. Based on these results, a signal transduction mechanism coupled to Mn oxidation was proposed.

## Introduction

Manganese is the second most abundant metallic element on Earth. It is widely distributed in soil minerals, seawater, neutral water and various sediments, and it plays an important role in biogeochemical cycles^1^. It is generally recognized that manganese oxide minerals in natural systems are formed by chemical and biological catalysis and oxidation. However, the biogenic Mn(II) oxidation mediated by microorganisms, especially a variety of bacteria that compound specific enzymes and metabolic pathways, dominates the biomineralization of Mn oxides because these biogenesis processes are much faster than abiotic catalysis by more than five orders of magnitude^2^. A variety of Mn(II)-oxidizing bacteria, typically the three model strains with high Mn(II)-oxidizing activity, i.e., *Bacillus* sp. SG-1^3^, *Leptothrix discophora* SS-1^4^, and *Pseudomonas putida* MnB1^5^ and GB-1^6^, have been characterized in terms of Mn(II) oxidation as an enzymatically catalysed cellular biochemical process. Despite the species diversity of Mn(II)-oxidizing bacteria, bacterial Mn(II) oxidization shares some common characteristics^2, 7^: for example, the oxidization process is characterized by the two-step consecutive mono-electron transfer of Mn(II) → Mn(III) → Mn(IV); the reaction requires oxygen and its main product is MnO_2_; and Mn(II) oxidation on the cell surfaces leads to the precipitation of oxides on cell surfaces.

Previous investigations have verified that the oxidation of soluble Mn(II) to Mn(III/IV) oxides is energetically favourable for Mn(II)-oxidizing bacteria^1^, and Mn(II) oxidation can also contribute to protect cells from damage caused by reactive oxygen species (ROS) or other free radicals^7^. Generally, bacterial Mn(II) oxidation is in itself a beneficial metabolic activity that requires various intracellular enzymatic pathways, such as multicopper oxidases (MCOs)^8, 9^, haem peroxidases^9, 10^, a two-component regulatory protein^11^, and even the influence of the surface-orientated flagella of host cells^12^, highlighting the multifactorial and symphyogenetic mechanisms of Mn(II) oxidation. Therefore, profiling the proteome of an Mn(II)-oxidizing bacterium during Mn(II) oxidation and the specification of the proteins associated with Mn(II) oxidation are of great significance. However, to date, there has been only limited investigation of the genome-wide response of a bacterial strain in the presence of Mn(II) compounds during its growth phase^13^, and very limited information is available concerning the global mechanisms and overall genetic requirements that control or contribute to Mn redox reactions that occur in a bacterial host cell.

The rapid development of proteomics incorporates mass spectrometry (MS) and bioinformatics technologies and provides an essential approach to investigate whole-cell variations in protein expression in response to Mn oxidation. High-throughput comparative proteomics enables the parsing of various potential mechanisms and regulatory networks of Mn oxidation, such as certain signalling pathways that sense and transduce Mn(II) stress signals, inducing manganese-oxidizing internal adaptive responses in bacteria and impeling the occurrence of Mn oxidation; an understanding of the timely bacterial metabolic adjustments to mediate the process of Mn(II) oxidation; and an understanding of the unique balance between the corresponding protein expression and ROS production and scavenging, which might drive Mn cycling and oxidation, and reduce the damage caused by oxidative stress. Therefore, proteomics is capable of serving as an effective tool to study protein composition and function in certain target Mn(II)-oxidizing bacterial cells on a large-scale, high-throughput and systematic level.

*Escherichia coli* MB266, a soil-borne Mn(II)-oxidizing bacterium isolated from a Fe-Mn nodule-surrounding soil sample, has been characterized by the ability to oxidize Mn(II) into Mn(IV) and to form microspherical aggregates in laboratory shake-flask trials^8^. Its multicopper oxidase, namely MCO266, has been confirmed to oxidize Mn(II) to Mn(III)/Mn(IV) oxides on the surface of host cells. X-ray photoelectron spectroscopy analysis demonstrated that the existence of Mn(IV) and Mn(III) oxides following Mn(II) oxidation by MB266 cells. The proportions of Mn(IV)-oxides, Mn(III)-oxides and Mn(II) in the total quantity of Mn oxides formed by MB266 were found to be 33.68%, 34.02%, and 32.29%, respectively^8^. Interestingly, gene disruption of *mco266* does not cause a complete loss of Mn(II) oxidation activity in MB266 cells^8^, supporting the involvement of other pathways in whole-cell Mn(II) oxidation. In this study, we performed an MS-based quantitative proteomics analysis of *E*. *coli* MB266 in different growth phases in the presence/absence of Mn(II), with the aims of investigating the comparative proteomic response to Mn(II) and potential Mn(II)-oxidizing or related genes in host cells. Moreover, the mRNA expression of 20 selected potential Mn(II) oxidation genes was determined by real-time quantitative polymerase chain reaction (RT-qPCR), and the activities of 5 candidate genes for Mn(II) oxidation were further validated by gene disruption and plasmid-driven expression complementation experiments. The presumed Mn(II) oxidation mechanism is also discussed.

## Results

### The growth and Mn oxidation phases of *E*. *coli* MB266

**Figure 1:**
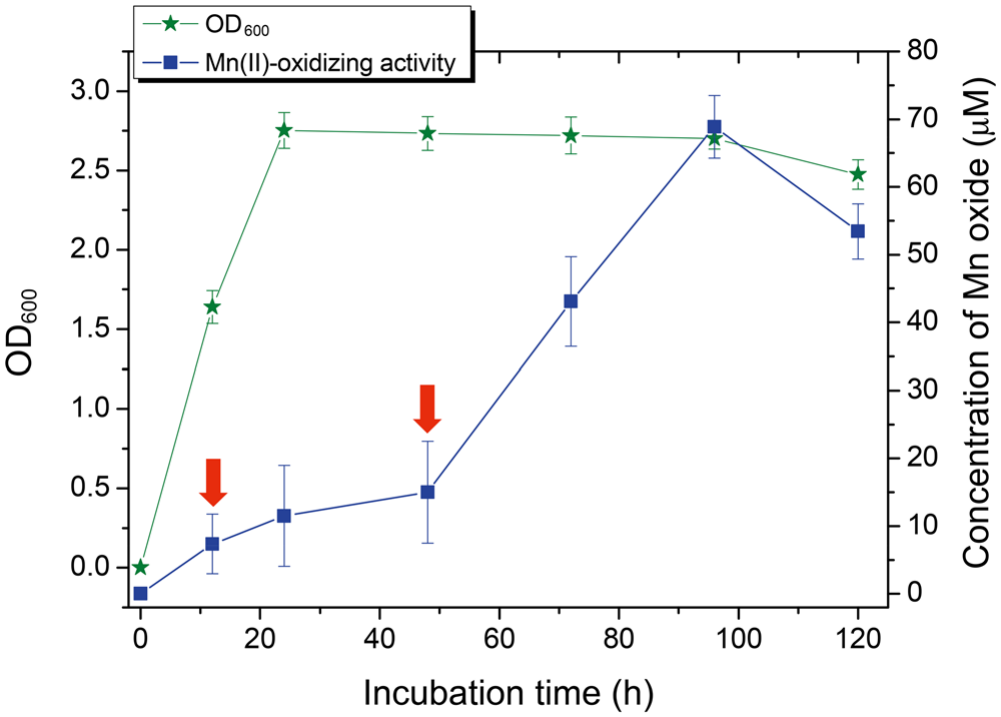
Growth curve and Mn oxidation activity of *E*. *coli* MB266. The green curve represents the growth curve of *E*. *coli* MB266. After inoculation in 250-mL flasks with a 1% inoculum size, the OD_600nm_ value of the culture was determined over a period of 5 d during which the cells were grown in liquid Lept medium. The blue curve shows the changes in the Mn oxidation activity of *E*. *coli* MB266. The time points used for the proteomic analyses are indicated by arrows.

*E*. *coli* MB266 were cultured in liquid Lept medium with or without 1 mM Mn(II) to quantify the Mn oxide concentration using a standard Leucoberbelin Blue (LBB) spectrophotometry assay. Figure 1 shows that the Mn(II)-oxidizing activity of MB266 cells increased steadily throughout the exponential (from approx. 3 to 24 h) and stationary (from approx. 24 to 96 h) growth phases, reached a maximum value (68.9 μM of the equivalent MnO_2_) at the end of the stationary growth phase (at approx. 96 h), and then slightly decreased at the decline growth phase (approx. 96 to 120 h). Moreover, although a sharp increase in cell density from 12 to 24 h, MB266 cells did not exhibit a synchronous sharp increase in Mn(II)-oxidizing activity during this time course, even when cell density was maintained stably from 24 to 48 h, MB266 cells exhibit only slight increase of Mn(II)-oxidizing activity from 24 to 48 h. However, MB266 cells exhibited a sharp increase in activity from 48 to 72 h. In view of slight increased Mn(II)-oxidizing activity started at about 12 h that might be coordinated to the initiation of Mn(II) oxidation, and substantial increase in activity started at about 48 h (Fig. 1, as indicated by the arrow), we therefore consider 12 h and 48 h to be appropriate time points for investigating the Mn(II)-oxidizing genes that respond to Mn(II) stress and are involved in Mn oxidation. Thus, MB266 cells grown in the presence/absence of 1 mM Mn(II) for 12 h and 48 h were sampled for the proteomics experiments.

### Overview of the proteomics data

**Figure 2:**
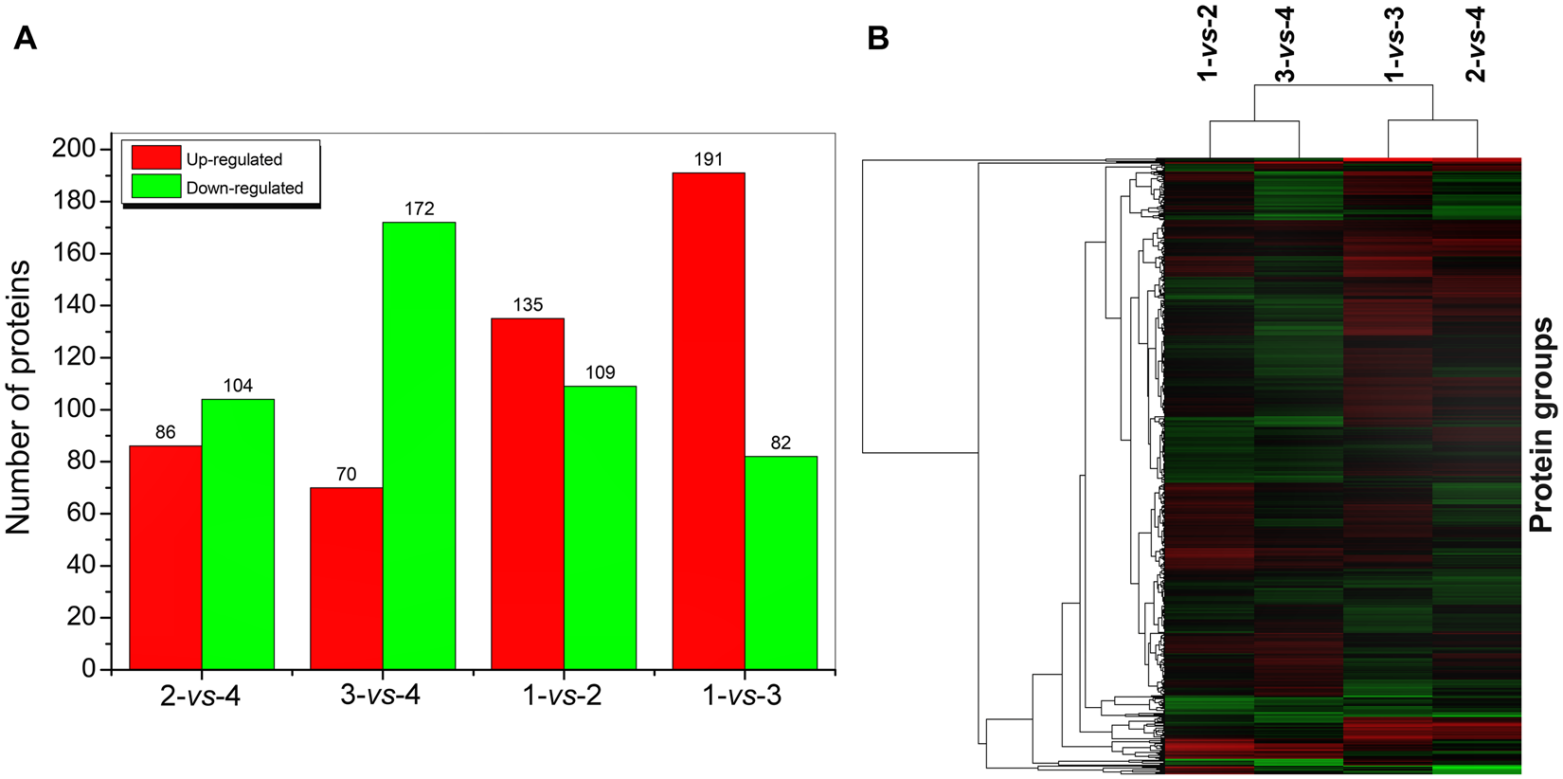
Differential protein expression and their cluster analysis of four sample pairwise comparisons. (**A**) The red and green coloured columns represent up- and down-regulated proteins, and the corresponding number of differentially expressed proteins is indicated. (**B**) The changing profiles of proteins that were differentially expressed more than 1.2-fold in any one comparison were hierarchically clustered. Different colours represent different expression folds of the proteins. Decreased and increased abundances are indicated by green and red shading, respectively. Each protein group (one per line) is shown along the y-axis, and the comparison groups are shown on the x-axis. Four sample pairwise comparisons were as follows: 1-*vs*-2, 12 h with 0 mM *versus* 1 mM Mn(II); 3-*vs*-4, 48 h with 0 mM *versus* 1 mM Mn(II); 1-*vs*-3, 12 h *versus* 48 h with 0 mM Mn(II); and 2-*vs*-4, 12 h *versus* 48 h with 1 mM Mn(II).

In the relative quantitative proteomics analysis, only those proteins with a fold change greater than 20% (a ratio of <0.833 for down-regulation or of >1.20 for up-regulation) and a *p*-value of <0.05 were considered to be differentially expressed^14^. To reveal the protein expression differences under specific conditions or at different growth phases, we designed four sample pairwise comparisons (Fig. 2A), i.e., 12 h with 0 mM *versus* 1 mM Mn(II) (namely 1-*vs*-2); 48 h with 0 mM *versus* 1 mM Mn(II) (3-*vs*-4); 12 h *versus* 48 h with 0 mM Mn(II) (1-*vs*-3); and 12 h *versus* 48 h with 1 mM Mn(II) (2-*vs*-4). Among the 1850 proteins that were identified by isobaric tags for relative and absolute quantitation (iTRAQ), 373 proteins were up-regulated (Supplementary Table S1) and 456 proteins were down-regulated (Supplementary Table S2) in at latest one pairwise comparison. In response to 1 mM Mn(II), 135 and 109 proteins were up-regulated and down-regulated at 12 h (1-*vs*-2), and 70 and 172 at 48 h (3-*vs*-4), respectively. In pairwise comparisons of the growth phase, the expression profiles of MB266 also displayed significant differences in the presence and absence of Mn(II) (2-*vs*-4 and 1-*vs*-3) (Fig. 2A). The 829 differentially expressed proteins induced by Mn(II) or related to the growth phases were subjected to hierarchical clustering analysis with a change threshold of at least 1.2-fold in abundance, which occurred in at least one comparison (Fig. 2B). These results indicated that two Mn(II) response- (1-*vs*-2 and 3-*vs*-4) and growth phase-related (1-*vs*-3 and 2-*vs*-4) pairwise comparisons could be separately clustered with each other, supporting the different effects of Mn(II) and growth phases on the differential protein-expression profiles of *E*. *coli* MB266.

### Global analysis of the iTRAQ results

The proteomic data showed that 57 identified DNA replication-related proteins and 27 transcription-related proteins remained nearly unchanged in both the 1-*vs*-2 and 3-*vs*-4 comparisons (Supplementary Table S3). However, among the identified 50 DNA repair-related proteins, different expression patterns were observed, such as in 1-*vs*-2. Most proteins in the 1-*vs*-2 comparison were up-regulated (9 increased *vs*. 4 decreased), whereas most were down-regulated in the 3-*vs*-4 comparison (14 decreased *vs*. 1 increased). These results indicated that the DNA repair system in MB266 exerted different responses to Mn(II) stress in the exponential growth phase compared with the stationary growth phase. Most of the aminoacyl-tRNA biosynthesis-related proteins for the 20 amino acids that were identified by iTRAQ remained unchanged in the four sample pairwise comparisons (Supplementary Table S4). Among 53 ribosomal structural proteins identified by iTRAQ, 9 and 5 were increased and decreased in 1-*vs*-2, respectively, while 16 were up-regulated but none were down-regulated in 3-*vs*-4. Notably, among the 21 identified preprotein translocases and signal peptidases, 8 and 6 proteins were up-regulated in 1-*vs*-2 and 3-*vs*-4, respectively, suggesting that the protein export and secretion system was activated by the addition of Mn(II). In addition, 62 identified proteins could potentially participate in signal recognition and transduction processes, of which 10 and 6 proteins were increased (including PspA and YgiM) and decreased, respectively (Supplementary Table S5). Furthermore, 55 identified proteins could be associated with inorganic ion transportation, of which 21 and 6 of these proteins were increased and decreased, respectively (Supplementary Table S6).

Because *E*. *coli* MB266 was still in the exponential growth phase at 12 h and did not initiate mass production of manganese oxides, the comparison of 0 mM *versus* 1 mM Mn(II) (1-*vs*-2) was of interest for finding the target proteins associated with the Mn(II) response and the possible precipitating factors of Mn oxidation. Upon Mn(II) addition, 135 proteins were up-regulated and 109 proteins were down-regulated (Fig. 2A). These proteins were classified into 21 different categories based on their predicted functions (Supplementary Fig. S1). For example, the ribonucleoside-diphosphate reductase 2 subunit beta Nrdf (YP_490891.1), which is related to nucleotide transport, was up-regulated by more than 3-fold (1-*vs*-2), which is consistent with a previous study that collectively showed that NrdEF could be activated *in vitro* by manganese and iron, and induced by H_2_O_2_ stress and iron starvation to enable cell replication^15^.

Bacterial cold shock proteins (CSPs) can function as mRNA chaperones and transcription antiterminators in response to temperature downshifts and other stresses^16^. The iTRAQ data revealed that among five identified CSPs (CspA, CspC, CspD, CspE and YfiA), CspA (the major cold shock protein), CspC and YfiA (the CSP associated with the 30 S ribosomal subunit) were markedly up-regulated in 1-*vs*-2, while CspE (a DNA-binding transcriptional repressor) remained unchanged in 1-*vs*-2. We presumed that these CSPs could play critical roles in transcriptional and post-transcriptional regulation in response to Mn(II) stress. CspD, a member of the CspA family of cold shock proteins, can inhibit DNA replication and is proposed to be associated with persister cell formation in *E*. *coli*. Moreover, CspD is regulated by cyclic AMP receptor protein (CRP) and is toxic to cells at high levels^17^. In addition, it is strongly induced under conditions of oxidative stress^18^. Interestingly, CspD is not induced during cold shock but is generally induced during the stationary phase in *E*. *coli* ^17^. CspD was significantly up-regulated during the exponential growth phase in the presence of 1 mM Mn(II) (1-*vs*-2) in *E*. *coli* MB266, supporting the involvement of CspD in the Mn(II) stress response.

*E*. *coli* MB266 entered the stationary growth phase and initiated high-volume production of manganese oxides at 48 h. In the 3-*vs*-4 comparison, 71 and 172 proteins were significantly up-regulated and down-regulated in the presence of Mn(II), respectively. More importantly, proteins related to the stress response and signal transduction (i.e., Spy), ROS response (i.e., SodA) and metal ion transportation (i.e., CirA) were up-regulated. These proteins might play a direct (such as catalysis) or indirect role in the process of Mn oxidation.

### Proteomic analysis of metabolic pathways and the material basis of Mn oxidation

**Figure 3:**
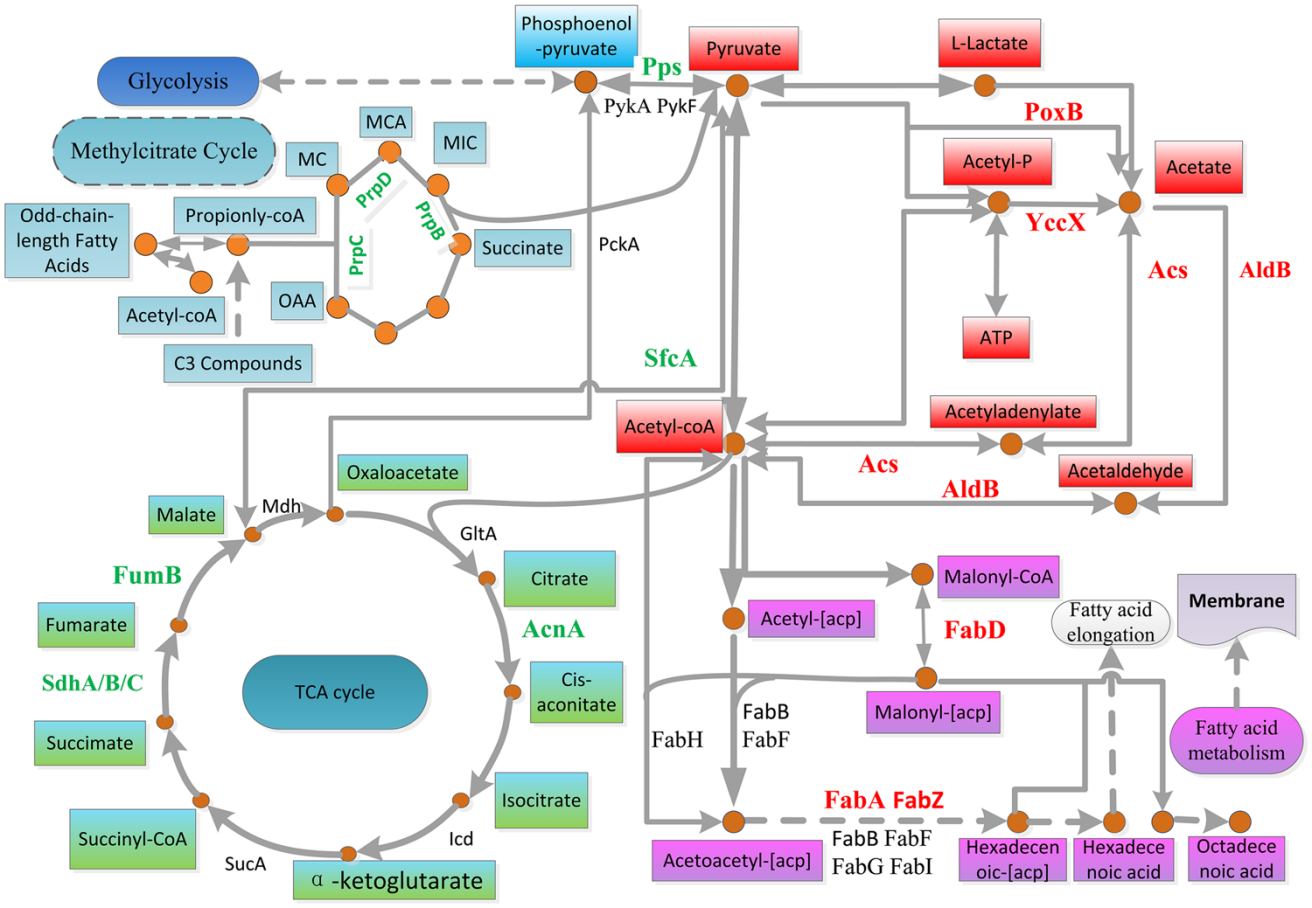
Metabolic pathways potentially associated with Mn oxidation and the Mn(II) stress response. The pathway included pyruvate metabolism, fatty acid metabolism and synthesis, the TCA cycle and glycolysis. The generally up- and down-regulated enzymes are represented in red and green, respectively.

Previous investigations have confirmed that a variety of metabolism-related genes are involved in bacterial Mn oxidation through certain metabolic pathways^5^. Elementary biological pathways such as the TCA cycle and amino acid metabolism not only supply the material basis for Mn oxidation but also provide the redox enzymes that are located directly on the surface of cells^1^. To investigate whether or how bacteria respond to Mn(II)-stimulating signals and implement efficient biological Mn oxidation processes through metabolic regulation in MB266, we performed a proteomics analysis of different metabolic pathways and established a related metabolic network (Fig. 3). The metabolic pathways included fatty acid synthesis, pyruvate metabolism, glycolysis, the citric acid cycle and the methylcitrate cycle, as well as their relationship with Mn oxidation, which was specified as follows.

#### Fatty acid metabolism

Fatty acid metabolism is closely related to the formation of cell membranes. The proteomics data showed that several key enzymes in fatty acid metabolism all tended to exhibit a substantial accumulation (Fig. 3). In the fatty acid biosynthesis pathway, several important enzymes, including acetyl-coA carboxylase (AccA, YP_488487.1), beta-hydroxydecanoyl thioester dehydrase (FabA, YP_489226.1), 3-oxoacyl-[acyl-carrier-protein] synthase I (FabB, YP_490565.1), malonyl-CoA-[acyl-carrier-protein] transacylase (FabD, YP_489360.1), alpha-hydroxysteroid dehydrogenase (FabG, YP_489361.1), 3-oxoacyl-[acyl-carrier-protein] synthase III (FabH, YP_489359.1), enoyl-[acyl-carrier-protein] reductase (FabI, YP_489556.1) and (3 R)-hydroxymyristol acyl carrier protein dehydratase (FabZ, YP_488482.1), were differentially up- or down-regulated in different comparisons. Among them, FabA, FabD and FabZ were up-regulated by 1.248 (2-*vs*-4), 1.241 (2-*vs*-4) and 1.23(1-*vs*-2), respectively, which can be attributed to the adaptive response to the Mn(II) ion stimulus. Upon Mn ion stimulation, the expression of these enzymes tended to increase. As a result, fatty acid synthesis was accelerated and led to the reinforcement of cellular membrane integrity, where Mn oxidation occurs.

#### Pyruvate metabolism

Pyruvic acid is one of the intermediates involved in the basic metabolism of organisms. Generally, abundant glucose flows into the glycolysis pathway to produce large amounts of pyruvate. Next, pyruvate can be used to: 1) yield acetyl-CoA for energy metabolism; 2) participate in the biosynthesis of fatty acids, acetoin and amino acids (particularly branched-chain amino acids, including isoleucine, leucine and valine); and 3) generate other metabolic intermediates such as lactate. In *E*. *coli*, the pyruvate dehydrogenase (Pdh) complex and pyruvate formate lyase (Pfl) are accepted as the main enzymes responsible for transferring pyruvate to acetyl-CoA under aerobic and anaerobic conditions, respectively. An important bypass for the conversion of pyruvate is mediated by pyruvate oxidase (encoded by *poxB*) and acetyl-CoA synthase (encoded by *acs*) (Fig. 3); PoxB is a peripheral membrane protein that catalyses the decarboxylation of pyruvate to produce acetate and CO_2_ with the concomitant reduction of quinones (UQH_2_)^19^. PoxB was originally regarded as a non-essential and potentially wasteful enzyme of uncertain metabolic function^20–23^. However, our results indicated that PoxB (YP_489144.1) was significantly up-regulated [by 1.716-fold (2-*vs*-4)]. Moreover, the genetic analysis confirmed that the Mn(II)-oxidizing activity of the MB266*ΔpoxB* mutant strain was decreased significantly (see below). Therefore, PoxB and the pyruvate metabolism pathway might be tightly associated with Mn oxidation.

Furthermore, as shown for the metabolism pathways, many other related proteins accumulated, including acyltransferase YqeF (YP_491049.1), aldehyde dehydrogenase B AldB (YP_491846.1), and acetyl-coA synthase Acs (YP_492212.1). Among them, acyltransferase YqeF (YP_491049.1) can convert acetyl-coA into acetoacetyl-coA, which is associated with the metabolism of methyl butyrate. Acylphosphatase YccX (YP_489240.1), which is utilized in the pathway for the transformation of pyruvate into acetic acid, was also significantly up-regulated. Moreover, acetyl-coA synthase Acs (YP_492212.1) was up-regulated by 1.392-fold (2-*vs*-4), which can catalyse the transformation of acetic acid into acetyl-coA via the intermediate acetyladenylate^23^ (Fig. 3). Aldehyde dehydrogenase B AldB (YP_491846.1) is thought to be essential for the removal of aldehydes and alcohols in cells under stress condition^24^. The results showed that AldB was up-regulated by 1.897-fold in the presence of Mn(II) (2-*vs*-4). Accordingly, we speculated that AldB could also be crucial during the process of Mn oxidation.

#### Tricarboxylic acid cycle pathway

Interestingly, it has been found that some enzyme-encoding genes for TCA, including genes encoding different subunits of succinate dehydrogenase and isocitrate dehydrogenase, are associated with the oxidation of Mn(II), as demonstrated by transposon insertion mutation analyses^5^. According to our proteomics data, 26 TCA-related proteins were identified, and 6 and 6 proteins were increased and decreased in at least one comparison, respectively (Supplementary Table S7). Among them, fumarate reductase FrdB (YP_492298.1) and fumarate hydratase FumB (YP_492265.1), which catalyse the mutual conversion of malate and fumarate, were down-regulated by 0.566-fold (0.742-fold in 2-*vs*-4) in the presence of Mn(II) during the stationary phase and 0.649-fold (3-*vs*-4), respectively. Moreover, SdhA, ShdB and SdhC in the succinate dehydrogenase complex were respectively down-regulated by 0.785-, 0.773- and 0.654-fold (3-*vs*-4), and aconitate hydratase AcnA (YP_489544.1) was down-regulated by 0.752-fold (1-*vs*-2). These results indicated that different steps of the TCA cycle exhibited various responses to Mn(II) under different conditions. Notably, the 2-methylisocitrate lyase PrpB (YP_488626.1), 2-methylcitrate synthase PrpC (YP_488627.1) and 2-methylcitrate dehydratase PrpD (YP_488628.1) in the methylcitrate cycle were all down-regulated in the presence of 1 mM Mn(II) in both the logarithmic phase (1-*vs*-2) and the stationary phase (3-*vs*-4), while they were all up-regulated in the stationary phase compared with the logarithmic phase by 3.627- and 5.738-fold, 3.659- and 5.056-fold, 3.758- and 7.08-fold with (2-*vs*-4) and without (1-*vs*-3) 1 mM Mn(II), respectively. The methylcitrate cycle can provide an additional supplement to the TCA pathway because it can completely oxidize the propionyl-CoA yielded mainly via the *β*-oxidation of odd-chain-length fatty acids^25^. Consequently, we speculated that the methylcitrate cycle might be inhibited to reduce the *β*-oxidation of odd-chain-length fatty acids, which would be very beneficial for membrane stability during conditions of Mn(II) stress; by contrast, it would be activated during the stationary phase under starvation stress.

Taken together, our proteomics data showed that Mn(II) led to major changes in the proteomics profile of MB266 at both protein number and abundance levels. The four important metabolic pathways were involved in manganese responses and oxidation, in which the pyruvate and fatty acid metabolism pathways were enhanced during the process of Mn oxidation, while the TCA pathway was inhibited to a certain degree.

### Spy, a key factor in the stress response and signal transduction systems of manganese oxidation

Several previous studies have suggested that two-component system (TCS) proteins are involved in the process of manganese oxidation^8, 11^. In this study, 11 TCS proteins from strain MB266 were identified by iTRAQ analysis (Supplementary Table S8). The results showed that a periplasmic protein, namely Spy (spheroplast protein Y) (YP_490004.1), was significantly up-regulated (by 1.61-fold at 48 h in the presence of Mn(II)) (3-*vs*-4); a hybrid sensory histidine kinase that senses intracellular oxygen and hydrogen peroxide, BarA (YP_490994.1), was increased by the addition of Mn(II) by 1.38-fold during the exponential phase (1-*vs*-2) and 1.43-fold during the stationary phase (3-*vs*-4). Moreover, the expression of the histidine kinase QseB (YP_491217.1), which is responsible for quorum sensing, was up-regulated in both 2-*vs*-4 and 3-*vs*-4, indicating that these proteins could be involved in Mn(II) stress response and oxidation processes.

Furthermore, 15 proteins associated with environmental pressure were identified in the proteomic data, of which 9 were clearly up-regulated (Supplementary Table S9), suggesting that Mn(II) could lead to the generation of external environmental pressure. It is believed that the extracytoplasmic stress response system (ESR) plays an important role in coupling envelope stress by regulating gene expression in response to stress signals^26^. Spy is homologous to CpxP, a well-studied negative regulator of Cpx signalling in *E*. *coli*, and it is highly conserved within the family *Enterobacteriaceae*. Expression of the *spy* gene is activated by the TCS CpxAR under envelope stress and Mn(II) shock, while Zn-induced expression is regulated by the TCS BaeSR^27, 28^. It is noteworthy that the expression of Spy in *E*. *coli* is normally induced during the spheroplasting phase^29^, the time at which Mn(II) was added to the medium. Therefore, the remarkably up-regulated expression of Spy upon Mn(II) addition supports the significance of this protein in Mn oxidation in MB266 cells.

Based on these results, we performed gene disruption/expression complementation experiments of the gene *spy* to verify its function in Mn oxidation. The Mn(II)-oxidizing activity of *E*. *coli* MB266 was decreased by approximately 37.8% as a consequence of *spy* gene disruption, and the activity was restored to a certain extent after expression complementation of the *spy* gene (see details below). These results suggested that Spy, which functions as a signal recognition protein in *E*. *coli* MB266, was accumulated in response to Mn(II) stress to initiate the ESR system, which in turn transduced signals to the cytoplasmic space across the inner membrane to regulate the expression of target genes^26^ that contribute to manganese oxidation.

### Mn(II) oxidation and reactive oxygen species (ROS) response

It is generally recognized that O_2_ is harmless to *E*. *coli* cells. However, when the distribution of electrons is altered, they can be converted into reactive oxygen species (ROS) and cause certain toxic reactions^30^. Aerobic metabolism generates ROS, such as superoxide, hydrogen peroxide and hydroxyl radicals, which may cause oxidative damage in living cells, in which oxidative stress can be caused by the action of free radicals, other ROS, and reactive nitrogen species (RNS)^31^. Several previous investigations have verified that ROS alone can oxidize Mn to protect bacteria^32^, and Mn(II) oxidation can scavenge ROS such as superoxide^33–35^. Moreover, *Roseobacter* sp. AzwK-3b has been shown to oxidize Mn(II) to generate Mn oxides through extracellular production of ROS superoxides^13^; an *Neisseria gonorrhoeae* strain can accumulate manganese via the ATP binding cassette (ABC)-type Mn transporter MntABC to prevent killing by O_2_^−^ and H_2_O_2_ by a superoxide dismutase (SOD)- and catalase-independent mechanism^36^; and in *Pseudomonas putida* GB-1, oxidative stress is apparently involved in intracellular Mn(II) oxidation^37^. All these results support a strong interconnection between Mn(II) oxidation and ROS stress in Mn(II)-oxidizing bacteria.

The proteomics data showed that at least 16 ROS scavenging-related proteins in *E*. *coli* MB266 could be identified by iTRAQ analysis and that their expression was influenced by the presence of Mn(II) (Table 1), suggesting that Mn(II) might serve as an antioxidant to activate a group of proteins related to antioxidant activity. These proteins included superoxide dismutase, reductases and several different catalases and peroxidases. Furthermore, the presence of these proteins revealed a strong connection with Mn(II) irrespective of the growth phase, (i.e., at 12 h or 48 h). Superoxide dismutase (Mn) SodA was particularly up-regulated, by approximately 2-fold (1-*vs*-2 and 3-*vs*-4) (Table 1), which suggested that organisms need to supplement superoxide dismutases to resist toxic oxygen metabolites. These results suggested that Mn oxidation in *E*. *coli* MB266 might be largely propelled by the presence of ROS.

**Table 1:** The proteins involved in the reactive oxygen species scavenging network of MB266.

Enzyme and reaction	Protein or domain	Accession	Description	1-*vs*-2^a^	3- *vs*-4	1- *vs*-3	2- *vs*-4
Superoxide and dismutase (SOD) O_2_^−^ + O_2_^−^ + 2H^+^ → H_2_O_2_ + O_2_	SodA	YP_491542.1	Superoxide dismutase, Mn	**2**.**036** ^b^	**1**.**756**	1.144	0.967
	SodB	YP_489920.1	Superoxide dismutase, Fe	0.677	0.999	0.85	**1**.**202**
	SodC	YP_489910.1	Superoxide dismutase, Cu, Zn	—	—	—	—
Catalase 2H_2_O_2_ → 2H_2_O + O_2_	KatE	YP_489993.1	Hydroperoxidase HPII(III)	0.874	1.155	1.19	**1**.**599**
	KatG	YP_491509.1	Catalase/hydroperoxidase HPI(I)	1.002	1.062	0.943	1.007
Glutathione reductase (GR) GSSG + NAD(P)H → 2GSH + NAD(P)^−^	Gor	YP_491935.1	Glutathione oxidoreductase	0.934	0.98	1.113	1.091
Glutathione peroxidase (GPX) H_2_O_2_ + 2GSH → 2H_2_O + GSSG	BtuE	YP_489972.1	Glutathione peroxidase	1.128	1.016	1.451	**1**.**288**
NADPH oxidase NADPH + e^−^ + O_2_ → NADP^−^ + O_2_^−^ + H^+^ ROS producer	AhpC	YP_488895.1	Peroxiredoxin, alkyl hydroperoxide reductase	1.049	1.087	0.99	1.087
	AhpF	YP_488896.1	Alkyl hydroperoxide reductase, large subunit	1.099	1.152	1.064	1.083
Thioredoxin and thioredoxin reductase H_2_O_2_ + Trx_red_ → Trx_ox_ + 2 H_2_O	TrxA	YP_491658.1	Thioredoxin 1	**1**.**254**	1.124	1.047	0.993
	TrxB	YP_489160.1	Thioredoxin reductase	1.174	0.983	1.292	1.105
	YbbN	YP_488783.1	Thioredoxin domain-containing protein	1.067	0.889	1.405	1.163
Ferredoxin	Fdx	YP_490753.1	[2Fe-2S] ferredoxin	**1**.**337**	0.672	1.249	0.621
Cytochrome H^+^ + Cytc_red_ → Cytc_ox_ + OH^−^	CyoA	YP_488724.1	Cytochrome c oxidase subunit 2	1.063	0.971	1.01	0.952
	CyoB	YP_488723.1	Cytochrome c oxidase subunit 1	1.03	1.122	1.055	1.01
	YhjA	YP_491917.1	Cytochrome c peroxidase	1.012	**1**.**217**	0.794	0.945

Note: ^a^1-*vs*-2, 12 h with 0 mM *vs*. 1 mM Mn (II); 3-*vs*-4, 48 h with 0 mM *vs*. 1 mM Mn (II); 1-*vs*-3, 12 h *vs*. 48 h with 0 mM Mn (II); and 2-*vs*-4, 12 h *vs*. 48 h with 1 mM Mn (II). ^b^Bold value represents up-regulated expression (>1.2 fold).

Superoxides are powerful and versatile redox effectors that have been confirmed to play a significant role in the bio-geochemical cycles of numerous metals, including the reduction of Fe(III) and Cu(II) and the oxidation of Mn(II)^38^. Because Mn(II) oxidation through superoxides can also generate ROS hydrogen peroxide (H_2_O_2_)^34^, some H_2_O_2_-degrading enzymes such as catalases and peroxidases were mobilized in the presence of Mn(II). For example, the catalases KatG and KatE were both identified, and katE was up-regulated by 1.599-fold at 48 h following the addition of Mn(II). During this period, protein maintenance and repair may be particularly important for bacterial survival^39^.

Taken together, our work could aid in understanding the unique balance of the corresponding protein expression and ROS production and scavenging, which might drive Mn cycling and oxidation. Moreover, bacterial Mn oxidation would be a complex biological catalytic process, and various factors and proteins could participate in the Mn oxidation process in an independent but synergic manner. In addition, many redox enzymes were found to be significantly up-regulated, including AcpD, Bfr, CydB, Fdx, PanE, SufI, YacK, YahK, YciW, YhdH, YheM and YncB, as well as the above-mentioned SodA, PoxB and KatE (Table 2). Research has shown that proteins such as cytochrome bd oxidase CydB (YP_489013.1) can reduce O_2_ via a single four-electron transfer reaction, and minimize the production of reactive oxygen species such as terminal oxidases^40^. YncB (YP_489714.1), a putative NAD(P)-binding oxidoreductase that belongs to the LTD-type MDR superfamily, can catalyse oxidation or reduction reactions^41^. These redox enzymes, together with MCOs, might be directly involved in the bacterial Mn oxidation process. It is worth noting that the 2-dehydropantoate reductase PanE was clearly inhibited in response to Mn(II) stress (1-*vs*-2 and 3-*vs*-4), but it was very significantly up-regulated (38.3-fold) during the stationary phase in comparison to the logarithmic phase (2-*vs*-4). Indeed, PanE can catalyse NADPH-dependent reduction of alpha-ketopantoate to form D-(-)-pantoate in the pantothenate/coenzyme A biosynthetic pathway. Moreover, pantothenate (commonly referred to as vitamin B5) is an essential molecule in the metabolism of living organisms and forms the core of coenzyme A^42^. Thus, the role of PanE in the Mn(II) stress response and oxidation is worth further investigation.

**Table 2:** Oxidoreductases identified by iTRAQ.

Protein or Domain	Accession	Description	1-*vs-*2	Sig^a^	3-*vs*-4	Sig	1-*vs*-3	Sig	2-*vs*-4	Sig
AcpD	YP_489679.1	NADH-azoreductase, FMN-dependent	1.021^b^		**1**.**281**	*	1.276	*	**1**.**788**	*
Bfr	YP_492096.1	Bacterioferritin, iron storage and de- toxification protein	0.735	*	0.878	*	1.375	*	**1**.**604**	*
CydB	YP_489013.1	Cytochrome d terminal oxidase, subunit II	0.726	*	0.894	*	1.223	*	**1**.**401**	*
Fdx	YP_490753.1	[2Fe-2S] Ferredoxin	**1**.**337**		0.672		1.249		0.621	
KatE	YP_489993.1	Hydroperoxidase HPII(III)	0.874	*	1.155	*	1.19	*	**1**.**599**	
PanE	YP_488717.1	2-Dehydropantoate reductase, NADPH- Specific	0.022		0.577		1.501		**38**.**308**	
PoxB	YP_489144.1	Pyruvate dehydrogenase (pyruvate oxi- dase, thiamin-dependent, FAD-binding	0.74	*	1.128	*	1.15	*	**1**.**716**	*
SodA	YP_491542.1	Superoxide dismutase, Mn	**2**.**036**	*	**1**.**756**	*	1.144		0.967	
SodB	YP_489920.1	Superoxide dismutase, Fe	0.677		0.999		0.85		**1**.**202**	*
YacK	YP_488426.1	Multicopper oxidase (laccase)	0.981		1.12		0.94		1.11	
YahK	YP_488620.1	Oxidoreductase	0.865	*	1.173		1.145	*	**1**.**299**	*
YciW	YP_489555.1	Oxidoreductase	0.788	*	0.862		1.229		**1**.**514**	*
YhdH	YP_488628.1	2-Methylcitrate dehydratase	0.778		0.665	*	7.08		**3**.**758**	
YheM	YP_492088.1	Intracellular sulfur oxidation protein	**1**.**426**		0.635		2.592		1.142	
YncB	YP_489714.1	Oxidoreductase	0.862		1.085		1.188		**1**.**464**	*

Note: ^a^Sig = significance, statistical test of expression difference. *Statistically significant; ^b^the number indicates the fold change of the differential proteins. The four sample pairwise comparisons were as follows: 12 h with 0 mM *vs*.1 mM Mn(II), 1-*vs*-2; 48 h with 0 mM *vs*. 1 mM Mn(II), 3-*vs*-4; 12 h *vs*.48 h with 0 mM M (II), 1-*vs*-3; and 12 h *vs*.48 h with 1 mM Mn(II), 2-*vs*-4. The bold values represent up-regulated expression (>1.2 fold).

### Mn(II) oxidation-related gene expression at the mRNA level by RT-qPCR analysis

Based on the proteomics data and previous literature, a total of 20 candidate genes (Supplementary Table S10) that could be associated with Mn(II) oxidation and the ROS response were selected for expression analyses. The results (Supplementary Fig. S2) showed that the relative transcription of a variety of flagellum-associated genes, signal recognition factors and redox enzymes was different. The alteration patterns (up- or down-regulation) of the genes at the mRNA level were mostly consistent with those observed at the protein level in the proteomic analysis. Some variations might be attributed to differences in post-transcriptional and post-translational modifications of mRNAs and proteins, respectively, as well as to differences in the rates of mRNA and protein degradation during bacterial growth^43^.

**Figure 4:**
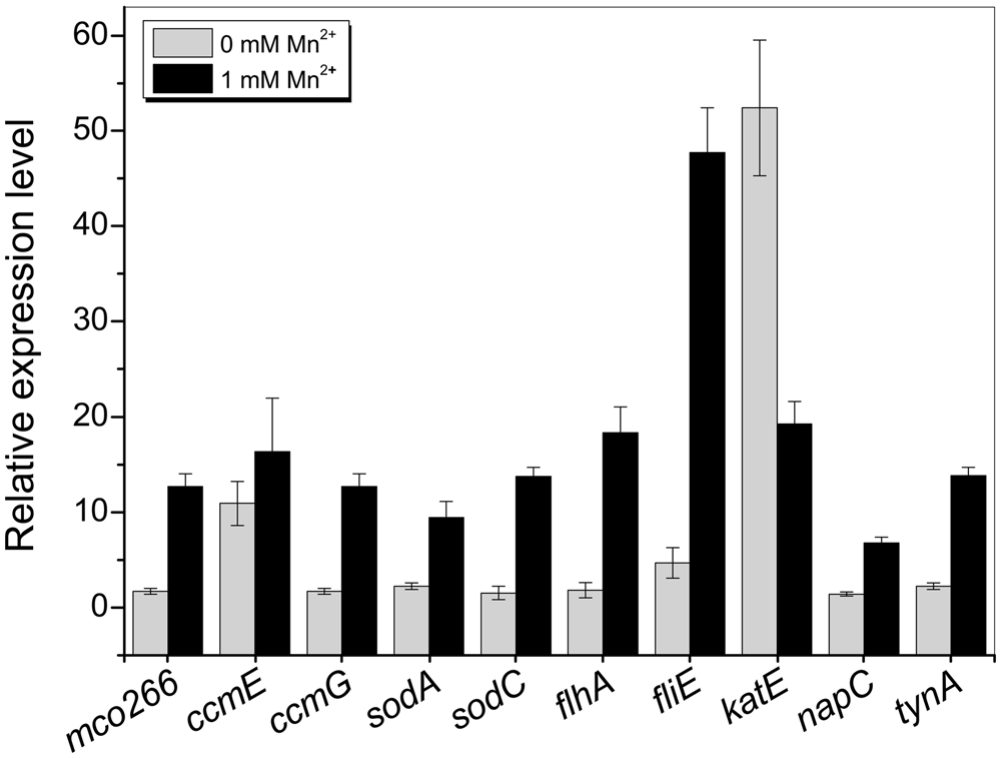
RT-qPCR analysis of the transcriptional activities of 10 representative genes related to Mn(II) oxidation. Total RNA was isolated from strain MB266 cultured for 48 h with or without 1 mM Mn(II) in Lept medium. The 16 S rRNA gene was used as a reference.

Among the 20 genes, 10 representative genes might be directly related to Mn oxidation: multicopper oxidase *mco266*, the flagella-related genes *fliA* and *fliE*, cytochrome C-related genes *ccmE* and *ccmG*, superoxidases *sodA* and *sodC*, catalase *katE*, and other genes such as *napC* and *tynA*. The RT-qPCR results indicated that the expression of most of these genes, such as *mco*, *sodA*, *katE* and *ccmG*, was up-regulated by more than 10-fold at 48 h (oxidation starting point) in the presence of Mn(II) in Lept medium, while differences in their expression were not apparent in the absence of Mn(II) (Fig. 4). Nevertheless, the relative transcriptional level of the other 10 genes, *aceK*, *fliC*, *fliG*, *fliS*, *icd*, *katG*, *motA*, *motB*, *sdhB* and *sdhC*, did not exhibit the same expression tendency. Details and RNA expression levels of all the genes are shown in Supplementary Figure S2.

### Mn oxidation activity verification of five key genes by gene disruption and complementation

**Figure 5:**
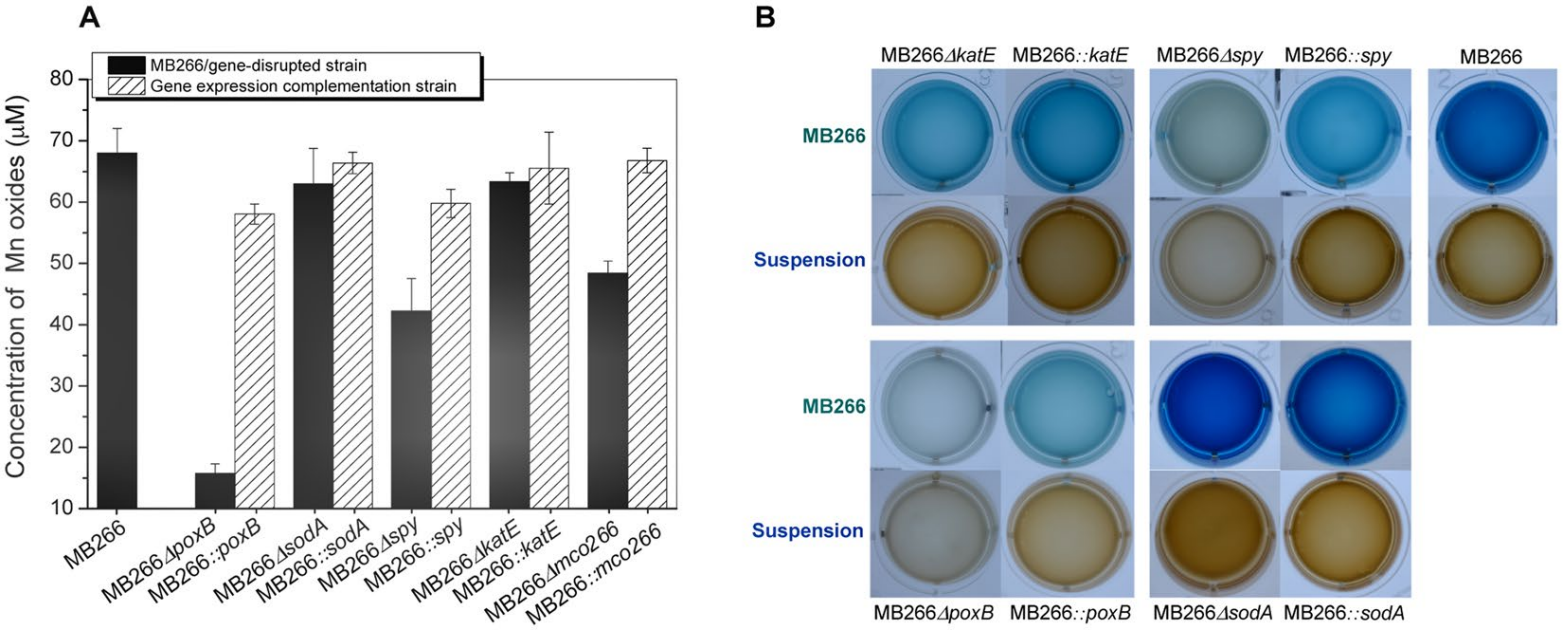
Measurement of Mn(II)-oxidizing activity in the gene disruption strains and corresponding complementation strains. Gene disruption and complementation strains of the key genes *poxB*, *sodA*, *spy* and *katE* were constructed. All the strains were grown in liquid Lept medium in the presence of 1 mM Mn(II) for 5 d. Subsequently, the Mn oxide concentrations of these strains were measured using the LBB method. The wild strain MB266 was used as a control.

To verify whether the genes in the metabolic pathways/ROS response/signal transduction pathway mentioned above were responsible for the Mn(II)-oxidizing activity of MB266 cells, gene-disrupted mutant strains corresponding to 5 key genes were constructed: *sodA* and *katE* (ROS response), *poxB* (metabolic pathway), *mco266* (multicopper oxidase) and *spy* (stress response and signal transduction). As shown in Fig. 5, the disruption of both *sodA* and *katE* caused only a slight decline in Mn(II)-oxidizing activity, and disruption of *spy* and *mco266* caused activity loss by 37.8% and 28.8%, respectively. However, surprisingly, disruption of the *poxB* gene conferred almost complete loss of Mn(II)-oxidizing activity, but complementation of this gene almost completely restored the activity. These results clearly demonstrated that *poxB*, *spy* and *mco266* are involved in Mn(II) oxidation in *E*. *coli* MB266.

## Discussion

The cooperative response of metabolic pathways and the combined action of a variety of enzymes are clearly important in the process of Mn oxidation of Mn(II)-oxidizing bacterium. However, limited information is available regarding the global gene expression of soil-borne bacteria with high Mn(II)-oxidizing activity. Accordingly, a macroscopic analysis of the differences in protein expression profiles of a bacterial strain grown with and without Mn(II) might provide new insight concerning the mechanisms of bacterial Mn oxidation. Therefore, we performed an in-depth quantitative proteomics analysis of a soil-borne *E*. *coli* MB266 coupled to Mn oxidation using the iTRAQ technique, and we mainly focused on the differential protein expression profiles of related metabolic pathways, the stress response, signal transduction pathways and ROS response pathways, with the aim of characterizing the global response and potential mechanisms responsible for Mn oxidation in a soil-borne bacterium model.

Mn(II) ions are generally recognized as a precipitating factor of cellular stress responses and signal transduction. They not only cause instability of the DNA structure through base damage or DNA pentose ring fracture, but they also lead to base pairing mistakes and instability of DNA polymerases^44^. Thus, Mn(II) ions in the intracellular compartment are thought to be able to induce gene mutations and increase the possibility of protein misfolding to some extent. Moreover, it is presumed that MB266 cells are capable of sensing outer membrane stress and osmotic pressure during Mn oxidation processes, as well as the cellular redox potential and concentration of terminal electron acceptors. Therefore, a stress response and signal transduction system should exist so that cells can adjust and adapt to the stress caused by Mn(II). Undoubtedly, oxidative removal of soluble Mn(II) to form insoluble Mn(III/IV) oxides via Mn(II) oxidation could be preferential for cells in terms of reducing the Mn(II) burden. As a result, these adaptive responses preferred the occurrence of cellular Mn oxidation. The stress and TCS-related protein Spy identified by iTRAQ, as well as the finding that the *spy* mutation caused a substantial reduction of Mn(II)-oxidizing activity, suggested that this protein could play a role in regulating Mn(II) oxidation. Based on the proteomics analysis, it was presumed that CSPs could contribute to transcriptional and post-transcriptional regulation in response to Mn(II) stress (Supplementary Table S11). Chemotaxis signal transduction is another special two-component signal transduction pathway. The signals are first sensed by MCPs, and then the active MCPs specifically activate the histidine kinase CheA; the activated CheA transmits the phosphate to the response regulator protein CheY. The CheY protein only contains the receptor domain; the output domain of normal response regulator proteins is lacking. The mobility of CheY is mainly based on the interaction between FliM and downstream proteins. This interaction affects the direction of rotation, thereby regulating the chemotactic response^45^. Similarly, the 18 CheY-associated proteins (Supplementary Table S12) identified by iTRAQ mainly belonged to the response regulators and consisted of a CheY-like receiver domain and a winged-helix DNA-binding domain or an HTH DNA-binding domain. Some of these proteins were down-regulated, such as PhoP(YP_489398.1) (3-*vs*-4 0.615-fold), while some were up-regulated, such as the two-component system regulatory protein QseB (YP_491217.1) (3-*vs*-4 1.558-fold). In *Roseobacter* sp., a group of proteins related to chemotaxis were analogously activated in the stress response to a high concentration of Mn(II)^13^. Therefore, the up-regulation of these chemotaxis-related proteins could imply the stress stimulation signalling role of manganese ions. Through MCP chemotaxis signal transduction, bacterial Mn oxidation was induced to regulate the Mn(II) ion concentration and thus to enable bacterial survival.

A variety of proteins, including superoxide dismutase, reductase, and several different catalases and peroxidases, were verified to be related to ROS^13^. Mn(II) is believed to serve as an antioxidant by scavenging ROS and can influence the expression of ROS-related proteins. In this study, we confirmed that the expression level of at least 16 ROS-related proteins identified from the iTRAQ data for MB266 (Table 1), were significantly influenced by the presence of Mn(II), consistent with a recent investigation showing that oxidative stress was closely related to intracellular Mn(II) levels^37^. These results suggest that the ROS response may also be involved in Mn oxidation processes.

**Figure 6:**
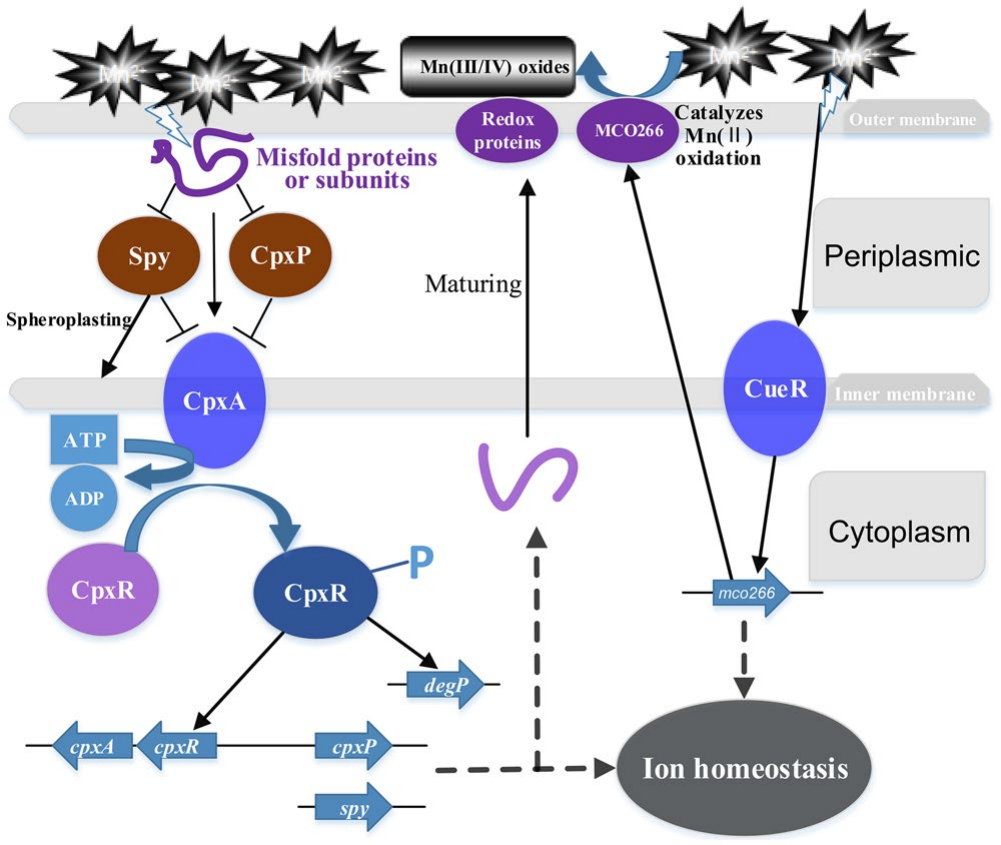
Predicted signal transduction mechanism associated with Mn oxidation and the stress response.

Based on the proteomics data, a signal transduction mechanism coupled to Mn oxidation in *E*. *coli* MB266 is proposed as follows (Fig. 6). After the addition of Mn(II), MB266 cells sensed extracellular Mn(II) ion stress via signal recognition proteins such as the extracytoplasmic stress response protein Spy. Subsequently, MB266 was immediately activated by the signal transduction systems and ESR to regulate the expression of many key genes to adjust to the stress environment. The key proteins might include fatty acid synthesis proteins (reinforcing the cell membrane against ion pressure), metal ion transport proteins (transport of metal ions to the membrane surface where Mn oxidation occurs), catalytic proteins and redox enzymes (direct involvement of Mn oxidation). Furthermore, the copper-responsive transcription factor CueR can activate the transcription of multicopper oxidase^46^ and then the multicopper oxidase Mco266 (GenBank accession number: JF682492) in MB266, leading directly to the formation of Mn oxides^8^. As a result, solid manganese oxides were generated and deposited around the bacterial cell surface to protect cells from UV/ROS/heavy metal damage.

## Materials and Methods

### Plasmids, strains, culture conditions and Mn(II)-oxidizing activity assay

The bacterial strains and plasmids used in this study are listed in Supplementary Table S13. The soil-borne bacterium *E*. *coli* MB266 was isolated from surface soil with Fe-Mn nodules in Shangdong Province, China^47^. The bacteria were cultured on Lept medium containing 20 mM 4-(2-hydroxyethyl)-1-piperazineethanesulfonic acid (HEPES pH 7.2) and 1 mM Mn(II) at 30 °C^8^. To produce Mn oxides, the overnight-cultured bacteria were inoculated into Lept medium at a ratio of 2% (V/V), and MnCl_2_ was added to the medium at a final concentration of 1 mM. The cultures were incubated continuously at 30 °C with shaking at 200 rpm for 5 d unless otherwise stated. The LBB method was used for Mn oxide quantification^48, 49^ with the addition of 1.0 mM MnCl_2_ (final concentration) in Lept medium. The *E*. *coli* WM3064 employed for the gene disruption and complementation assay were cultured at 37 °C in Luria−Bertani medium. When needed, sucrose (10% w/v), ampicillin (Amp, 100 μg/mL), kanamycin (Kan, 50 μg/mL) or gentamicin (Gm, 50 μg/mL) was added^50^.

### Protein sample preparation

The 12 h and 48 h growth time points were considered appropriate for the proteomic analysis (Fig. 1). The cells grown in Lept medium with or without 1 mM Mn(II) were collected by centrifugation (13,400 × *g*, 10 min, at 4 °C) and washed three times with ice-cold TE buffer (pH 7.4). The samples were sonicated at 200 W for 15 min and then centrifuged at 4 °C, 30,000 × *g* for 15 min. The proteins were extracted with a lysis buffer (7 M urea, 2 M thiourea, 4% CHAPS, 40 mM Tris-HCl, pH 8.5) containing 1 mM PMSF and 2 mM EDTA (final concentration). After 5 min, 10 mM DTT (final concentration) was added to the samples, and then the samples were centrifuged at 4 °C and 30,000 × *g* for 15 min. The supernatants were mixed well with a 5 × volume of chilled acetone containing 10% (v/v) TCA and incubated at −20 °C overnight. After centrifugation at 4 °C and 30,000 × *g*, the supernatants were discarded. The precipitates were washed three times with chilled acetone. The pellets were air-dried and dissolved in lysis buffer (7 M urea, 2 M thiourea, 4% NP40, 20 mM Tris-HCl, pH 8.0–8.5). The suspensions were sonicated at 200 W for 15 min and centrifuged at 4 °C and 30,000 × *g* for 15 min. The resulting supernatants were transferred to new tubes. To reduce disulfide bonds in the supernatant proteins, 10 mM DTT (final concentration) was added, and the samples were incubated at 56 °C for 1 h. Subsequently, 55 mM IAM (final concentration) was added to block the cysteines, by incubation for 1 h in the darkroom. The supernatants were mixed well with a 5 × volume of chilled acetone for 2 h at −20 °C to precipitate the proteins. After centrifugation at 4 °C and 30,000 × *g*, the supernatants were discarded, and the pellets were air-dried for 5 min, dissolved in 500 μL of 0.5 M TEAB (Applied Biosystems, Milan, Italy), and sonicated at 200 W for 15 min. Finally, the samples were centrifuged at 4 °C and 30,000 × *g* for 15 min. The supernatants were transferred to new tubes and quantified. The proteins in the supernatants were stored at −80 °C for further analysis.

### iTRAQ labelling and SCX fractionation

One hundred micrograms of total protein were collected from each sample solution and digested with Trypsin Gold (Promega, Madison, WI, USA) at a protein: trypsin ratio of 30: 1 at 37 °C for 16 h. After trypsin digestion, the peptides were dried by vacuum centrifugation. The peptides were reconstituted in 0.5 M TEAB and processed according to the manufacturer’s protocol for 8-plex iTRAQ reagent (Applied Biosystems). Briefly, one unit of iTRAQ reagent was thawed and reconstituted in 24 μL of isopropanol. The samples were labelled with iTRAQ tags as follows: Sample 1 (119 tag), Sample 2 (113 tag), Sample 3 (115 tag) and Sample 4 (117 tag). The peptides were incubated at room temperature for 2 h for labelling with isobaric tags. The labelled peptide mixtures were then pooled and dried by vacuum centrifugation. Subsequently, SCX chromatography was performed with a LC-20AB HPLC Pump system (Shimadzu, Kyoto, Japan). The iTRAQ-labelled peptide mixtures were reconstituted with 4 mL of buffer A (25 mM NaH_2_PO_4_ in 25% ACN, pH 2.7) and loaded onto a 4.6 × 250 mm Ultremex SCX column containing 5-μm particles (Phenomenex). The peptides were eluted at a flow rate of 1 mL/min with a gradient of buffer A for 10 min, 5–60% buffer B (25 mM NaH_2_PO_4_, 1 M KCl in 25% ACN, pH 2.7) for 27 min and 60–100% buffer B for 1 min. The system was then maintained with 100% buffer B for 1 min before equilibration with buffer A for 10 min prior to the next injection. Elution was monitored by measuring the absorbance at 214 nm, and fractions were collected every 1 min. The eluted peptides were pooled into 20 fractions, desalted with a Strata X C18 column (Phenomenex) and vacuum-dried.

### LC-ESI-MS/MS analysis based on Triple TOF 5600

Each fraction was resuspended in buffer A (5% ACN, 0.1% FA) and centrifuged at 20,000 × *g* for 10 min. The final concentration of peptides was approximately 0.5 μg/μL. Ten microliters of the supernatant was loaded onto a 2-cm C18 trap column on a LC-20AD nanoHPLC (Shimadzu, Kyoto, Japan) by the autosampler. The peptides were eluted onto a 10-cm analytical C18 column (inner diameter of 75 μm) that was packed in-house. The samples were loaded at 8 μL/min for 4 min, and then a 35-min gradient was run at 300 nL/min beginning with 2 to 35% B (95% ACN, 0.1% FA), followed by a 5-min linear gradient to 60%, followed by a 2-min linear gradient to 80%, maintenance at 80% B for 4 min, and finally a return to 5% in 1 min.

Data acquisition was performed using a TripleTOF 5600 System (AB SCIEX, Concord, ON) fitted with a Nanospray III source (AB SCIEX, Concord, ON) and a pulled quartz tip as the emitter (New Objectives, Woburn, MA). Data were acquired using an ion spray voltage of 2.5 kV, curtain gas of 30 psi, nebulizer gas of 15 psi, and an interface heater temperature of 150 °C. The MS was operated with an RP greater than or equal to 30,000 FWHM for the TOF MS scans. For IDA, survey scans were acquired in 250 ms, and as many as 30 product ion scans were collected if a threshold of 120 counts per second (counts/s) and a 2+ to 5+ charge state were exceeded. The total cycle time was fixed at 3.3 s. The Q2 transmission window was 100 Da for 100%. Four time bins were summed for each scan at a pulser frequency value of 11 kHz by monitoring the 40 GHz multichannel TDC detector with four-anode channel detection. A sweeping collision energy setting of 35 ± 5 eV coupled to iTRAQ was used to adjust the rolling collision energy applied to all precursor ions for collision-induced dissociation. Dynamic exclusion was set at 1/2 of the peak width (15 s), and then the precursor was refreshed off the exclusion list.

### Bioinformatics analysis

Raw data files acquired from the Orbitrap were converted into MGF files using Proteome Discoverer 1.2 (PD 1.2, Thermo). Protein identification was performed using the Mascot search engine (Matrix Science, London, UK; version 2.3.02) against the NCBI database: *Escherichia coli* str. K-12 substr. W3110 (with 4164 entries; http://www.ncbi.nlm.nih.gov/genome/167? project_id = 161931). For protein identification, only peptides with significance scores (≥20) at the 99% confidence interval based on a Mascot probability analysis greater than “identity” were counted as identified to reduce the probability of false peptide identification. Each identified protein included at least one unique peptide. For protein quantitation, a protein had to contain at least two unique peptides. The quantitative protein ratios were weighted and normalized by the median ratio in Mascot. Only *p*-values of <0.05 and fold changes of >1.2 were considered significant.

The KEGG pathway is a collection of manually drawn pathway maps representing our knowledge of the molecular interaction and reaction networks. Molecules are represented as nodes, and the biological relationships between two nodes is represented as an edge (line).

### Quantitative RT-PCR analysis

*E*. *coli* MB266 were cultured in Lept medium with or without 1 mM Mn(II), and the cells were collected by centrifugation (13,400 × *g*, 10 min, at 4 °C) after 12 h, 24 h 48 h and 72 h. Total RNA was extracted using TRIzol reagent according to the manufacturer’s instructions (ComWin Biotech, Beijing, China). The RNA samples were treated with RNase-free DNase I to remove genomic DNA contamination. The quality and quantity of the RNA samples were evaluated using a spectrophotometer (NanoDrop 2000, Thermo). Reverse transcription was performed with the RevertAid™ First Strand cDNA Synthesis Kit (Fermentas). The obtained cDNA was diluted 10-fold for further qRT-PCR analysis with SYBR Green Real-time PCR Master Mix (Takara). The primers used for the reactions are listed in Supplementary Table S14. Gene expression was normalized by F = 2^−ΔΔt^ analysis according to the instructions provided by Bioteke Corporation company (Beijing, China). The 16 S rRNA gene was used as a reference in the calculations.

### Gene disruption and complementation assay

As shown in Supplementary Fig. S3, markerless mutants were constructed using the SOE PCR technique^50^. A fused fragment of the target gene was inserted into the pDS3.0 suicide vector (Sucrose^s^, Gm^r^) via a *Sac*I restriction site. The newly constructed vector was then transferred into *E*. *coli* WM3064, a *dap* genetic defect-type strain. Subsequently, the suicide vector was transferred into *E*. *coli* MB266 (recipient strain) by conjugation. After an overnight incubation, the bacterial lawn was scratched and spread on LB agar containing 50 μg/mL Gm at the final concentration to isolate the merodiploid MB266. Mutants carrying the first homologous recombination (single crossover between the constructed plasmid and the chromosome of MB266) were screened for sucrose-negative and Gm-positive phenotypes. To screen second homologous recombination mutants, a single crossover mutant was grown in LB medium, and the bacterial colonies were screened for sucrose-positive and Gm-negative phenotypes. To confirm the gene knockout, the primers used for confirmation were designed based on sequences flanking further upstream and downstream regions. For gene complementation, a fragment of the target gene was inserted into pTrcHis B/C by treatment with restriction enzymes. The plasmids were then separately transformed into the corresponding mutant strains. Primer details and a schematic of the gene knock out methodology are presented in Supplementary Table S15 and Fig. S3, respectively.

## Electronic supplementary material

Supplementary material
