# Supplementary material for: Mechanistic insights into manganese oxidation of a soil-borne Mn(II)-oxidizing Escherichia coli strain by global proteomic and genetic analyses
Source: Sci Rep. 2017 May 2;7:1352. doi: 10.1038/s41598-017-01552-3 (PMC5430989; doi:10.1038/s41598-017-01552-3)
Supplement: Supplementary file 1 — Supplementary material [file 41598_2017_1552_MOESM1_ESM.pdf]

Supporting Information

Mechanistic insights into manganese oxidation of a soil-borne Mn(II)-oxidizing *Escherichia coli* strain by global proteomic and genetic analyses

Zhiyong Wang[1], Jieping Wang[2], Jin Liu[1], Hong Chen[1], Mingshun Li[1], Lin Li[1,*]

[1]State Key Laboratory of Agricultural Microbiology, Huazhong Agricultural University, Wuhan 430070, China;

[2]Agricultural Bio-resources Institute, Fujian Academy of Agricultural Sciences, Fuzhou 350003, China.

* Corresponding author. Phone: +86-27-8728 6952; Fax: +86-27-8728 0670; E-mail: lilin@mail.hzau.edu.cn

List of supplementary tables and figures

1. **Table S1.** All of the up-regulated proteins identified by iTRAQ······················3
2. **Table S2.** All of the down-regulated proteins identified by iTRAQ··················30
3. **Table S3.** Up- or down-regulated proteins related to DNA replication, transcription and translation···63
4. **Table S4.** Up- or down-regulated protein synthesis related proteins··················83
5. **Table S5.** All of the signal transduction related proteins·····························100
6. **Table S6.** All of the inorganic ion transport and metabolism related proteins······105
7. **Table S7.** All of the metabolism pathway-related proteins···························109
8. **Table S8.** The two-component regulatory system related proteins··················123
9. **Table S9.** The stress response-related proteins···124
10. **Table S10.** The genes selected for RT-qPCR analysis of transcriptional activity···125
11. **Table S11.** Heat shock proteins and cold shock proteins identified by iTRAQ··128
12. **Table S12.** The CheY-related proteins identified by iTRAQ·························129
13. **Table S13.** *E. coli* strains and plasmids used in this study··························130
14. **Table S14.** The oligonucleotide primers used for RT-qPCR analyses··············132
15. **Table S15.** The oligonucleotide primers used for gene disruption and complementation··134
16. **Figure S1.** Functional classification of differential expressed proteins in the pairwise comparison 1-vs-2··135
17. **Figure S2.** Real time quantitative PCR analysis of the transcriptional activities of 20 selected genes··136
18. **Figure S3.** Schematic illustration of in-frame gene disruption strategy for *E. coli* MB266 target genes using the suicide vector pDS3.0 recombinant system········137

Table S1. All of the up-regulated proteins identified by iTRAQ.[a–c]

Protein or Domain	Accession	Description	1-vs-2	Sig	3-vs-4	Sig	1-vs-3	Sig	2-vs-4	Sig
AccB	YP_491437.1	acetyl CoA carboxylase, BCCP subunit	1.2	*	0.959		1.224		1.097	
AceA	YP_492158.1	isocitrate lyase	1.128		1.154		1.176		1.208	
AceK	YP_492159.1	isocitrate dehydrogenase kinase/phosphatase	1.065		1.014		1.338		1.242	
AcpD	YP_489679.1	NADH-azoreductase, FMN-dependent	1.021		1.281	*	1.276	*	1.788	*
AcrA	YP_488754.1	multidrug efflux system	1.309	*	0.935		1.179	*	0.88	*
Acs	YP_492212.1	bifunctional acetyl-CoA synthetase/propionyl-CoA synthetase	0.864		0.999		1.114		1.392	
AdhC	YP_488650.1	alcohol dehydrogenase class III/glutathione-dependent formaldehyde dehydrogenase	1.059		1.253	*	1.015		1.132	*
AdhP	YP_489743.1	alcohol dehydrogenase, 1-propanol preferring	1.126	*	0.86	*	2.782	*	1.857	*
AldA	YP_489682.1	aldehyde dehydrogenase A, NAD-linked	1.176		1.254		1.218		1.34	
AldB	YP_491846.1	aldehyde dehydrogenase B	0.857	*	0.866	*	1.81	*	1.897	*
AmiC	YP_491022.1	N-acetylmuramoyl-L-alanine amidase	0.985		1.057		1.135		1.205	
AmyA	YP_490184.1	cytoplasmic alpha-amylase	0.836		0.945		1.606		1.683	

ApbA	YP_488717.1	2-dehydropantoate reductase, NADPH-specific	0.022		0.577		1.501		38.308	
AphA	YP_492198.1	acid phosphatase/phosphotransferase, class B, non-specific	1.633	*	0.887		1.053		0.664	*
Apt	YP_488760.1	adenine phosphoribosyltransferase	1.157	*	1.239	*	0.861	*	0.922	
ArgD	YP_492072.1	bifunctional acetylornithine aminotransferase/succinyldiaminopimelate	0.919		1.182		1.448		1.844	
ArgE	YP_491495.1	acetylornithine deacetylase	1.053		1.341	*	0.842		1.092	
ArgR	YP_491421.1	DNA-binding transcriptional dual regulator	1.231		1.605		0.803		1.036	
ArtI	YP_489136.1	arginine transporter subunit	1.298	*	0.947		1.414		0.972	
AscG	YP_490923.1	DNA-binding transcriptional regulator	0.946		1.542		1.059		1.13	
Asd	YP_492000.1	aspartate-semialdehyde dehydrogenase	1.204	*	1.003		1.094		0.979	
AstA	YP_490008.1	arginine succinyltransferase	1.076		1.071		1.98	*	1.737	*
AstB	YP_490006.1	succinylarginine dihydrolase	0.686	*	1.367		0.972		1.917	*
AstD	YP_490007.1	succinylglutamic semialdehyde dehydrogenase	0.908	*	0.838	*	1.91	*	1.726	*
AtpA	YP_491695.1	F1 sector of membrane-bound ATP synthase subunit alpha	1.262		1.058		1.274	*	1.075	
AtpC	YP_491698.1	F1 sector of membrane-bound ATP synthase subunit epsilon	1.33		1.167	*	1.398	*	1.175	*

AtpD	YP_491697.1	F1 sector of membrane-bound ATP synthase subunit beta	1.412		1.01		1.337		1.016	
AtpH	YP_491694.1	F1 sector of membrane-bound ATP synthase subunit delta	1.372	*	1.118		1.126	*	0.969	
BarA	YP_490994.1	hybrid sensory histidine kinase, in two-component regulatory system with UvrY	1.383		1.433	*	0.977		1.002	
BetB	YP_488607.1	betaine aldehyde dehydrogenase	1.207	*	1.089		1.07		1.015	
Bfr	YP_492096.1	bacterioferritin, iron storage and detoxification protein	0.735	*	0.878	*	1.375	*	1.604	*
BioH	YP_492020.1	carboxylesterase of pimeloyl-CoA synthesis	1.192		1.347		0.971		1.086	
BirA	YP_491483.1	bifunctional biotin-[acetylCoA carboxylase] holoenzyme synthetase/DNA-binding	1.277		1.089		1.192		1.006	
BolA	YP_488727.1	regulator of penicillin binding proteins and beta lactamase transcription (morphogene)	0.897		0.755		2.542	*	1.843	
BtuE	YP_489972.1	glutathione peroxidase	1.128		1.016		1.451	*	1.288	*
BtuR	YP_489538.1	cob(I)alamin adenolsyltransferase/cobinamide ATP-	0.926		1.292		0.761		1.051	
Cdd	YP_490382.1	cytidine/deoxycytidine deaminase	1.019		1.222	*	1.005		0.994	
CirA	YP_490394.1	ferric iron-catecholate outer membrane transporter	2.595		2.558	*	1.41		1.072	
Crr	YP_490653.1	PTS system glucose-specific transporter subunit IIA	1.095	*	1.034		1.336	*	1.206	
CsdA	YP_491018.1	cysteine sulfinate desulfinase	0.873		0.831		1.494		1.579	

CsiE	YP_490763.1	stationary phase inducible protein	1.193		0.9		1.685	*	1.374	
CspA	YP_491880.1	major cold shock protein	1.567	*	0.915		0.746	*	0.396	*
CspC	YP_490084.1	stress protein, member of the CspA-family	1.765		1.235	*	0.78	*	0.536	*
CspD	YP_489153.1	cold shock protein	3.245	*	0.627		0.961		0.184	*
CstC	YP_490009.1	succinylornithine transaminase, PLP-dependent	0.866	*	0.825		2.301	*	2.008	
CycA	YP_492350.1	D-alanine/D-serine/glycine transporter	0.993		0.892		1.236		1.209	*
CyoA	YP_488724.1	cytochrome o ubiquinol oxidase subunit II	1.063		0.971		1.01		0.952	
CyoB	YP_488723.1	cytochrome o ubiquinol oxidase subunit I	1.03		1.122		1.055		1.01	
CysH	YP_490971.1	3~-phosphoadenosine 5~-phosphosulfate reductase	0.64		0.962		1.088	*	1.62	
CysK	YP_490650.1	cysteine synthase A, O-acetylserine sulfhydrolase A subunit	0.859		1.042		1.165		1.444	
CysM	YP_490657.1	cysteine synthase B	1.15		1.055		1.286	*	1.326	*
CysQ	YP_492356.1	PAPS (adenosine 3~-phosphate 5~-phosphosulfate) 3~(2~),5~-bisphosphate	1.334		0.902		1.887	*	1.362	
DacA	YP_488923.1	D-alanyl-D-alanine carboxypeptidase	0.962		0.935		1.299	*	1.233	*
DacC	YP_489112.1	D-alanyl-D-alanine carboxypeptidase	0.836	*	1.006		1.106		1.536	

DadX	YP_489457.1	alanine racemase 2, PLP-binding	0.911		0.99		1.304	*	1.36	*
DamX	YP_492044.1	hypothetical protein Y75_p3788	1.26		1.213		1.232		1.118	*
Dcp	YP_489801.1	dipeptidyl carboxypeptidase II	0.837	*	0.902	*	1.152	*	1.307	
Def	YP_492146.1	peptide deformylase	0.975		1.23	*	0.686	*	0.923	
DicA	YP_489834.1	regulator for DicB	1.22		1.538	*	0.923		1.129	
DkgA	YP_491205.1	2,5-diketo-D-gluconate reductase A	0.859	*	0.943		1.441	*	1.703	
DnaG	YP_491258.1	DNA primase	1.499		0.974		1.299	*	0.836	
DppA	YP_491891.1	dipeptide transporter	1.021		1.036		1.398	*	1.389	
DsbA	YP_491589.1	periplasmic protein disulfide isomerase I	1.234	*	0.905	*	1.184	*	0.838	*
DsbC	YP_491094.1	protein disulfide isomerase II	1.787		0.856		1.356		0.642	
Dtd	YP_491563.1	D-Tyr-tRNA(Tyr) deacylase	1.499	*	0.981		1.815	*	1.176	
ElaB	YP_490506.1	hypothetical protein Y75_p2230	0.748		0.911		1.268		1.527	
Eno	YP_490987.1	enolase	1.236		1.099		1.252	*	1.023	
EntA	YP_488885.1	2,3-dihydro-2,3-dihydroxybenzoate dehydrogenase	1.719		1.497		1.053		0.908	

EntB	YP_488884.1	isochorismatase	2.961	*	2.203	*	1.107		0.932	
EntF_1	YP_488875.1	enterobactin synthase multienzyme complex component, ATP-dependent	1.901	*	1.588	*	1.084		0.808	
Exo	YP_491006.1	exonuclease IX (5~-3~ exonuclease)	0.939		1.094		1.171		1.351	
ExuR	YP_491285.1	DNA-binding transcriptional repressor	1.086		1.21		1.092		0.889	
FabA	YP_489226.1	beta-hydroxydecanoyl thioester dehydrase	0.89	*	0.928		1.235	*	1.248	*
FabD	YP_489360.1	malonyl-CoA-[acyl-carrier-protein] transacylase	1.04		1.067		1.195	*	1.241	
FabZ	YP_488482.1	(3R)-hydroxymyristol acyl carrier protein dehydratase	1.23		1.093	*	1.189	*	1.098	*
FadA	YP_491599.1	3-ketoacyl-CoA thiolase	0.965		0.869		1.552		1.442	*
FadD	YP_490066.1	acyl-CoA synthetase	0.912		0.81		1.687		1.533	
FadH_1	YP_491272.1	2,4-dienoyl-CoA reductase	0.836		0.935		1.211	*	1.412	
FadL	YP_490586.1	long-chain fatty acid outer membrane transporter	1.015		0.84		1.658	*	1.324	*
FbaB	YP_490337.1	fructose-bisphosphate aldolase class I	0.96		1.009		1.171		1.218	
FeoB	YP_492023.1	fused ferrous iron transporter, protein B	2.335		0.897		0.999		0.659	
Fic	YP_492070.1	stationary-phase protein, cell division	0.988		0.856		2.023		1.735	*

Fiu	YP_489078.1	iron outer membrane transporter	2.484	*	1.879		1.672	*	0.861	
FkpA	YP_492085.1	FKBP-type peptidyl-prolyl cis-trans isomerase	1.982	*	1.453		0.982		0.666	*
FldA	YP_488964.1	flavodoxin 1	1.203	*	0.904		1.358	*	1.066	
GabD	YP_490876.1	succinate-semialdehyde dehydrogenase I, NADP-dependent	1.022		1.036		1.585	*	1.621	
GabT	YP_490877.1	4-aminobutyrate aminotransferase, PLP-dependent	1.069	*	0.9	*	1.663	*	1.455	
GalK	YP_489030.1	galactokinase	1.228		0.904		1.109		0.882	
GalS	YP_490390.1	DNA-binding transcriptional repressor	1.254		1.038		1.152	*	0.944	
GalU	YP_489504.1	glucose-1-phosphate uridylyltransferase	0.966		1.201		0.802	*	0.933	
GlgB	YP_492001.1	1,4-alpha-glucan branching protein	1.044		0.969		1.383		1.301	
GlnH	YP_489084.1	glutamine transporter subunit	1.637	*	1.138		1.037		0.701	*
GlnK	YP_488742.1	nitrogen assimilation regulatory protein for GlnL, GlnE, and AmtB	2.72	*	2.287	*	1.272		0.974	
GlnP	YP_489083.1	glutamine transporter subunit	1.437		0.898		1.621	*	1.003	
GlnQ	YP_489082.1	glutamine transporter subunit	1.153		1.275		0.936		0.942	
Gltl	YP_488946.1	glutamate and aspartate transporter subunit	1.301	*	0.838	*	1.308	*	0.834	

Gmk	YP_491786.1	guanylate kinase	1.324	*	1.793	*	0.727	*	0.933	
GntY	YP_492018.1	gluconate transport associated protein	1.027		1.059		1.307		1.326	
GreA	YP_491366.1	transcription elongation factor	1.085	*	1.022		1.309	*	1.236	
GrxC	YP_491823.1	glutaredoxin 3	2.153	*	1.659	*	0.766	*	0.615	*
GyrB	YP_491736.1	DNA gyrase, subunit B	0.988		1.217		0.799		0.999	
HdhA	YP_489882.1	7alpha-hydroxysteroid dehydrogenase	0.908	*	1.115	*	1.366	*	1.714	*
HlpA	YP_488480.1	periplasmic chaperone	2.161	*	1.14		1.775	*	0.97	
HtrA	YP_488464.1	serine endoprotease (protease Do), membrane-associated	1.916		1.27	*	1.308	*	0.952	
HupA	YP_491460.1	HU, DNA-binding transcriptional regulator subunit alpha	1.577	*	1.445	*	0.776		0.653	*
HupB	YP_488732.1	HU, DNA-binding transcriptional regulator subunit beta	1.502	*	1.054		0.953		0.67	*
HypE	YP_490939.1	carbamoyl phosphate phosphatase, hydrogenase 3 maturation protein	39.393		0.527		72.8		0.963	
IlvD	YP_490874.1	hypothetical protein Y75_p2602	1.115	*	0.945		2.429	*	2.176	
IlvI	YP_488383.1	acetolactate synthase III, large subunit	1.295		1.164		1.089		0.969	
Imp	YP_488360.1	exported protein required for envelope biosynthesis and integrity	1.071		1.102		1.225	*	1.254	*

IscU	YP_490757.1	scaffold protein	1.445	*	1.117		1.329	*	0.86	*
IspA	YP_488713.1	geranyltranstransferase	0.89		0.874		1.299		1.262	
KatE	YP_489993.1	hydroperoxidase HPII(III)	0.874	*	1.155	*	1.19	*	1.599	
KdsA	YP_489482.1	3-deoxy-D-manno-octulosonate 8-phosphate synthase	1.253	*	1.34		0.989		1.027	
KduI	YP_491048.1	5-keto 4-deoxyuronate isomerase	1.576		1.092		0.875		0.6	*
LacZ	YP_488638.1	beta-D-galactosidase	1.491		1.014		1.25	*	0.941	
LasT	YP_492533.1	rRNA methyltransferase	1.021		0.892		1.653		1.43	
LolA	YP_489163.1	chaperone for lipoproteins	1.529	*	0.81		1.286		0.674	
LolD	YP_489385.1	outer membrane-specific lipoprotein transporter subunit	1.457	*	1.222		1.139		0.904	
LpxA	YP_488483.1	UDP-N-acetylglucosamine acetyltransferase	1.053		1.632	*	0.713	*	1.072	
LpxB	YP_488484.1	tetraacyldisaccharide-1-P synthase	1.397		1.028		1.422		1.035	
Lrp	YP_489161.1	DNA-binding transcriptional dual regulator	1.285	*	1.048		0.865	*	0.853	
LuxS	YP_490901.1	S-ribosylhomocysteinase	1.379	*	0.947		1.235	*	0.86	*
MalE	YP_492177.1	maltose transporter subunit	1.557		1.094		1.043		0.725	*

ManA	YP_489876.1	mannose-6-phosphate isomerase	0.962		1.201		0.842		0.907	*
ManX	YP_490078.1	fused mannose-specific PTS enzyme IIAB components	1.23	*	1.028		1.169	*	1.014	
MdoG	YP_489317.1	glucan biosynthesis protein, periplasmic	1.289	*	1.123		1.051		0.93	
MenG	YP_491522.1	ribonuclease E (RNase E) inhibitor protein	1.215	*	1.067		1.3	*	1.231	*
MetC	YP_491201.1	cystathionine beta-lyase, PLP-dependent	1.332	*	1.395	*	1.041		1.034	
MetE	YP_491613.1	5-methyltetrahydropteroyltriglutamate-homocysteine S-methyltransferase	1.387	*	1.133		1.047		1.024	
MetK	YP_491141.1	methionine adenosyltransferase 1	1.285	*	1.195	*	1.018		0.969	
MetN	YP_488502.1	DL-methionine transporter subunit	1.004		1.536		0.684		1.036	
MglA	YP_490388.1	methyl-galactoside ABC transporter ATP-binding protein	1.238	*	1.141	*	0.981		0.972	
MglB	YP_490389.1	methyl-galactoside transporter subunit	1.783		1.13	*	1.15		0.715	*
MinC	YP_489443.1	cell division inhibitor	0.911		1.411	*	0.818		1.171	
MioC	YP_491687.1	FMN-binding protein MioC	1.52	*	1.16		1.257		0.843	
Mll1012	YP_492369.1	sugar transporter subunit	1.295		0.928		1.642		1.165	
NagA	YP_488957.1	N-acetylglucosamine-6-phosphate deacetylase	1.048		1.181		1.135		1.284	*

NagE	YP_488959.1	fused N-acetyl glucosamine-specific PTS enzyme IICBA components	1.322		1.034		1.134		0.961	
NanE	YP_491407.1	N-acetylmannosamine-6-P epimerase	1.288		1.161	*	0.993		0.976	
NanK	YP_491406.1	N-acetylmannosamine kinase	1.287	*	1.133		1.099		0.917	
Ndh	YP_489377.1	respiratory NADH dehydrogenase 2/cupric reductase	1.315		0.835	*	1.139		0.89	
NrdA	YP_490473.1	ribonucleoside diphosphate reductase 1 subunit alpha	1.396		1.43		0.84		0.947	
NrdB	YP_490474.1	ribonucleoside diphosphate reductase 1 subunit beta, ferritin-like	1.18	*	1.218	*	1.037		1.069	
NrdF	YP_490891.1	ribonucleoside-diphosphate reductase 2 subunit beta, ferritin-like	3.268	*	2.178		1.466	*	0.892	
OppA	YP_489511.1	oligopeptide transporter subunit	1.069		1.233		0.873		0.978	
OsmC	YP_489747.1	osmotically inducible, stress-inducible membrane protein	0.904		1.119	*	1.642	*	1.847	
OsmE	YP_490000.1	DNA-binding transcriptional regulator	0.89		0.935		1.109		1.339	
OtsA	YP_490157.1	trehalose-6-phosphate synthase	0.927		0.976		1.349		1.542	
PaaG	YP_489663.1	acyl-CoA hydratase	1.121		1.318		0.8		0.931	
Pal	YP_489021.1	peptidoglycan-associated outer membrane lipoprotein	0.976		1.162	*	0.96		1.249	*
PanB	YP_488437.1	3-methyl-2-oxobutanoate hydroxymethyltransferase	0.938		1.072		1.007		1.216	

Pcm	YP_490952.1	L-isoaspartate protein carboxylmethyltransferase type II	0.853		1.022		1.046		1.24	
PfkB	YP_489984.1	6-phosphofructokinase II	0.928		0.91		1.262		1.283	*
Pfs	YP_488462.1	5~-methylthioadenosine/S-adenosylhomocysteine nucleosidase	0.869		0.985		1.082		1.215	
PhnA	YP_492251.1	phosphonate metabolizing protein	1.103		1.581		0.715		1.006	
PhnB	YP_492250.1	hypothetical protein Y75_p3994	0.956		1.196		2.994	*	3.721	*
PitA	YP_491942.1	phosphate transporter, low-affinity	1.386		1.462		1.085	*	1.133	
PlsC	YP_491210.1	1-acyl-sn-glycerol-3-phosphate acyltransferase	1.102		1.389		0.647		0.84	
PncA	YP_490029.1	nicotinamidase/pyrazinamidase	0.954		1.067		1.123		1.253	
PotA	YP_489394.1	polyamine transporter subunit	0.989		0.841	*	1.257	*	1.306	*
PotD	YP_489391.1	polyamine transporter subunit	1.25		1.132		1.115		0.964	
PoxB	YP_489144.1	pyruvate dehydrogenase (pyruvate oxidase), thiamin-dependent, FAD-binding	0.74	*	1.128	*	1.15	*	1.716	*
PpiA	YP_492068.1	peptidyl-prolyl cis-trans isomerase A	1.572		1.249	*	1.077		0.833	*
PpiB	YP_488814.1	peptidyl-prolyl cis-trans isomerase B	1.201		0.971		1.093		1.082	
PpiC	YP_491663.1	peptidyl-prolyl cis-trans isomerase C	1.021		1.304	*	0.892		1.091	

PqiB	YP_489223.1	paraquat-inducible protein B	1.415	*	0.862		1.645		1.179	
ProA	YP_488538.1	gamma-glutamylphosphate reductase	1.01		1.186		1.187	*	1.208	*
ProP	YP_492254.1	proline/glycine betaine transporter	0.849		0.855		1.252		1.248	
ProV	YP_490892.1	glycine betaine transporter subunit	1.454	*	1.303	*	1.013		0.87	
ProX	YP_490894.1	glycine betaine transporter subunit	1.708		1.068		1.297	*	0.789	
PspA	YP_489572.1	regulatory protein for phage-shock-protein operon	1.286	*	2.761	*	0.964		2.095	*
PspE	YP_489576.1	thiosulfate:cyanide sulfurtransferase	2.168	*	0.76		0.656		0.187	*
PstB	YP_491704.1	phosphate transporter subunit	1.137		1.501	*	0.814		1.102	
PstS	YP_491701.1	phosphate transporter subunit	1.73	*	1.331	*	0.95		0.674	*
PurA	YP_492319.1	adenylosuccinate synthetase	0.928		1.065		1.008		1.209	
PurH	YP_491454.1	fused IMP cyclohydrolase and phosphoribosylaminoimidazolecarboxamide	0.717	*	0.884	*	1.23	*	1.502	*
PurM	YP_490727.1	phosphoribosylaminoimidazole synthetase	0.934		0.884		1.315	*	1.208	*
PurT	YP_490111.1	phosphoribosylglycinamide formyltransferase 2	0.585		0.728	*	1.199		1.647	*
Qor	YP_492194.1	quinone oxidoreductase, NADPH-dependent	1.026		1.139	*	1.287		1.319	

QseB	YP_491217.1	DNA-binding response regulator in two-component regulatory system with QseC	1.106		1.558		0.88		1.227	*
QseC	YP_491218.1	sensory histidine kinase in two-component regulatory system with QseB	0.879		0.899		1.129		1.24	*
RbsA	YP_491680.1	fused D-ribose transporter subunits and ATP-binding components ABC superfamily	1.312	*	1.028		1.087		0.985	
RbsB	YP_491678.1	D-ribose transporter subunit	2.028	*	0.95		1.407		0.686	*
RbsD	YP_491681.1	cytoplasmic sugar-binding protein	2.312		1.018		0.959		0.418	
RecD	YP_491024.1	exonuclease V (RecBCD complex) subunit alpha	1.513		1.089		0.888		0.632	
RfaB	YP_491805.1	UDP-D-galactose:(glucosyl)lipopolysaccharide-1, 6-	1.522	*	0.769		2.128		1.064	
RfaI	YP_491806.1	UDP-D-galactose:(glucosyl)lipopolysaccharide-	1.201		1.206		1.083		1.076	*
RfaP	YP_491803.1	kinase that phosphorylates core heptose of lipopolysaccharide	0.943	*	1.07		1.399	*	1.572	*
RibB	YP_491233.1	3,4 dihydroxy-2-butanone-4-phosphate synthase	1.762	*	0.583	*	1.068		0.34	*
RibH	YP_488707.1	riboflavin synthase beta chain	1.359	*	0.927		1.357		0.912	
RimM	YP_490831.1	16S rRNA processing protein	1.251	*	1.067		1.183		1.045	
RpiA	YP_491114.1	ribosephosphate isomerase	1.227		1.004		1.058		0.905	
RplC	YP_492112.1	50S ribosomal protein L3	1.403	*	1.334	*	1.036		0.95	

RplF	YP_492127.1	50S ribosomal protein L6	1.199	*	1.299		0.931	*	0.995	
RplK	YP_491478.1	50S ribosomal protein L11	1.24	*	0.952		1.211	*	0.91	
RplU	YP_491371.1	50S ribosomal protein L21	1.054		1.218	*	0.873	*	0.95	
RplV	YP_492117.1	50S ribosomal protein L22	1.214	*	1.406		0.937		1.083	
RplW	YP_492114.1	50S ribosomal protein L23	0.668		1.366	*	0.834		1.687	
RplX	YP_492123.1	50S ribosomal protein L24	1.302		1.472	*	0.765	*	0.931	
RpmB	YP_491796.1	50S ribosomal protein L28	1.261	*	1.521	*	1.014		1.127	*
RpmD	YP_492130.1	50S ribosomal protein L30	0.971		1.283	*	0.862	*	0.996	
RpmF	YP_489357.1	50S ribosomal protein L32	0.733	*	1.46	*	0.846	*	1.762	*
RpsL	YP_492090.1	30S ribosomal protein S12	1.491	*	1.171		1.138		1.038	
RpsN	YP_492125.1	30S ribosomal protein S14	2.161	*	2.247		1.017		0.994	
RpsR	YP_492344.1	30S ribosomal protein S18	1.387	*	1.447	*	0.93		1.075	
RpsS	YP_492116.1	30S ribosomal protein S19	1.045		1.437	*	0.645		0.969	
RpsT	YP_488329.1	30S ribosomal protein S20	1.601		1.587		1.145		1.102	

RpsU	YP_491257.1	30S ribosomal protein S21	2.398	*	2.674	*	1.299		1.286	*
SecB	YP_491824.1	protein export chaperone	1.327	*	1.257		0.843	*	0.901	
SecF	YP_488701.1	SecYEG protein translocase auxillary subunit	1.263		1.017		1.061		0.894	
SecG	YP_491360.1	preprotein translocase membrane subunit	1.104		1.577		0.569	*	0.769	
SelD	YP_490025.1	selenophosphate synthase	1.131	*	1.185	*	1.216	*	1.233	*
SixA	YP_490582.1	phosphohistidine phosphatase	1.517		1.076		1.477		1.037	
SodA	YP_491542.1	superoxide dismutase, Mn	2.036	*	1.756	*	1.144		0.967	
SppA	YP_490027.1	protease IV	1.311		1.136		1.249		1.074	
Spy	YP_490004.1	envelope stress induced periplasmic protein	1.174	*	1.614	*	0.969		1.501	*
SrlD	YP_490914.1	sorbitol-6-phosphate dehydrogenase	1.538	*	1.073		1.347	*	0.88	
SrlE	YP_490912.1	PTS system glucitol/sorbitol-specific transporter subunit IIB	1.751		2.118	*	0.771		0.923	
SurA	YP_488359.1	peptidyl-prolyl cis-trans isomerase	1.29		0.997		1.104		0.869	
TalA	YP_490691.1	transaldolase A	0.862	*	0.967		1.181	*	1.518	
Tam	YP_489782.1	trans-aconitate methyltransferase	1.647		0.46	*	2.193		0.606	*

TbpA	YP_488374.1	thiamin transporter subunit	1.234		0.946		1.04		0.882	
Tdh	YP_491817.1	threonine 3-dehydrogenase	0.957		1.215	*	0.815	*	1.009	
Tgt	YP_488698.1	tRNA-guanine transglycosylase	1.237	*	1.185		0.954		1.04	
TktB	YP_490692.1	transketolase 2, thiamin-binding	0.85		0.907		1.281		1.423	
TolB	YP_489020.1	hypothetical protein Y75_p0720	1.253	*	1.162		1.205		1.101	
TolC	YP_491227.1	transport channel	1.33	*	0.927		1.761	*	1.224	*
TpiA	YP_491532.1	triosephosphate isomerase	0.924		1.217		1.012		1.256	
Tpx	YP_489593.1	lipid hydroperoxide peroxidase	1.33		1.149	*	1.037		1.002	
TrpS	YP_492048.1	tryptophanyl-tRNA synthetase	1.208	*	1.396	*	0.777		0.984	
TrxA	YP_491658.1	thioredoxin 1	1.254		1.124	*	1.047		0.993	
TrxC	YP_490810.1	thioredoxin 2	1.52		0.783		1.31		1.067	
TyrR	YP_489592.1	DNA-binding transcriptional dual regulator	1.198	*	0.954		1.057		0.859	*
Udp	YP_491611.1	uridine phosphorylase	1.104		1.058		1.283	*	1.21	
UidA	YP_489880.1	beta-D-glucuronidase	0.964		1.326	*	1.025		1.271	*

UlaR	YP_492333.1	DNA-binding transriptional dual regulator	0.88		1.285	*	0.932		1.265	
XylF	YP_491868.1	D-xylose transporter subunit	1.249	*	1.057		1.213	*	1.014	
YacK	YP_488426.1	multicopper oxidase (laccase)	0.981		1.12		0.94		1.11	
YadF	YP_488429.1	carbonic anhydrase	1.398	*	1.21	*	1.056		0.961	
YaeC	YP_488500.1	DL-methionine transporter subunit	1.24		0.994		1.139		0.923	
YaeT	YP_488479.1	hypothetical protein Y75_p0173	1.329		1.086		1.163	*	0.972	
YafH	YP_488518.1	acyl coenzyme A dehydrogenase	1.002		1.027		1.641	*	1.63	*
YafJ	YP_488520.1	amidotransfease	1.027		1.131		1.146		1.249	
YagE	YP_488564.1	lyase/synthase	1.575	*	0.784		1.25		0.759	
YahD	YP_488613.1	transcriptional regulator with ankyrin domain	1.218		1.135		0.978		1.028	
YahK	YP_488620.1	oxidoreductase	0.865	*	1.173		1.145	*	1.299	*
YahO	YP_488624.1	hypothetical protein Y75_p0319	1.268		0.942		1.555		1.103	
YajC	YP_488699.1	SecYEG protein translocase auxillary subunit	1.209	*	1.333	*	0.939		0.968	
YajD	YP_488702.1	hypothetical protein Y75_p0398	1.485		1.227		0.967		0.87	

YajG	YP_488726.1	lipoprotein	1.238	*	1.275	*	1.254		1.222	*
YbaR	YP_488775.1	copper transporter	1.11		0.976		1.199		1.034	
YbaW	YP_488735.1	hypothetical protein Y75_p0431	0.735		0.871		1.815	*	2.399	*
YbaY	YP_488745.1	outer membrane lipoprotein	1.043		0.921		1.479	*	1.359	*
YbbU	YP_488796.1	DNA-binding transcriptional regulator	0.888		1.492	*	0.719	*	1.125	
YbgI	YP_488990.1	metal-binding protein	1.287	*	0.939		1.214	*	1.065	
YbgK	YP_488992.1	hypothetical protein Y75_p0692	0.844		0.841		1.074		1.464	
YbiU	YP_489094.1	hypothetical protein Y75_p0794	1.799		1.169		1.114		0.717	
YbjL	YP_489120.1	transporter	1.192		1.219		0.984		0.995	
YbjU	YP_489143.1	L-allo-threonine aldolase, PLP-dependent	0.843		1.041		0.989		1.206	
YbjY	YP_489151.1	macrolide transporter subunit, membrane fusion protein (MFP) component	0.941		0.897		1.372	*	1.295	
YcaC	YP_489169.1	hydrolase	1.006		1.181		1.455	*	1.787	*
YcaR	YP_489189.1	hypothetical protein Y75_p0889	1.707		0.844		1.155		0.565	
YcbL	YP_489199.1	metal-binding enzyme	1.088		1.239		0.901		0.951	

YcbX_1	YP_489219.1	2Fe-2S cluster-containing protein	1.185		1.264	*	1.231		1.181	
YccJ	YP_489276.1	hypothetical protein Y75_p0976	1.488	*	1.092		1.594	*	1.224	*
YccX	YP_489240.1	acylphosphatase	0.732		1.567	*	0.814		1.725	
YcdO	YP_489289.1	hypothetical protein Y75_p0990	1.762	*	1.38		1.211		0.784	
YceI	YP_489324.1	hypothetical protein Y75_p1026	1.404	*	0.976		1.269	*	1.036	
YcgB	YP_489455.1	hypothetical protein Y75_p1160	0.941		1.037		1.624	*	1.938	
YcgF_2	YP_489430.1	FAD-binding phosphodiesterase	0.852		0.92		1.117	*	1.33	
YcgM	YP_489447.1	isomerase/hydrolase	1.06		1.373		0.893		1.206	
YcgT	YP_489467.1	dihydroxyacetone kinase, N-terminal domain	1.214	*	1.294	*	1.15	*	1.168	
YciI	YP_489519.1	hypothetical protein Y75_p1225	1.329	*	1.064		1.075		0.885	
YciN	YP_489541.1	hypothetical protein Y75_p1247	1.045		1.349	*	0.85	*	0.931	
YciT	YP_489552.1	DNA-binding transcriptional regulator	1.27		0.91		1.27		0.901	
YdaA	YP_489603.1	stress-induced protein	0.901		1.202		0.888	*	1.148	
YdcF	YP_489681.1	hypothetical protein Y75_p1390	1.025		0.922		1.665	*	1.209	

YdcJ	YP_489689.1	hypothetical protein Y75_p1399	0.813		0.886		2.645	*	2.7	
YdcW	YP_489709.1	medium chain aldehyde dehydrogenase	0.967		0.94		1.696	*	1.521	*
YddE	YP_489729.1	hypothetical protein Y75_p1440	1.151	*	1.298		1.037		1.015	
YdeN	YP_489763.1	hypothetical protein Y75_p1474	1.733		0.844		1.519		0.787	
YdjF	YP_490031.1	DNA-binding transcriptional regulator	1.049		1.23		0.646		0.864	
YdjS	YP_490005.1	succinylglutamate desuccinylase	0.892		1.077		1.556	*	1.991	*
YeaE	YP_490042.1	oxidoreductase	1.242		1.332		1.153		1.223	
YeaF	YP_490043.1	scaffolding protein for murein synthesizing machinery	1.293	*	1.352		1.235	*	1.3	
YeaG	YP_490044.1	hypothetical protein Y75_p1758	0.989		1.072		2.143		2.24	
YeaT	YP_490060.1	DNA-binding transcriptional regulator	1.696		0.994		0.878		0.509	
YeaY	YP_490067.1	lipoprotein	1.209		1.195		1.248		1.395	*
YebC	YP_490126.1	hypothetical protein Y75_p1840	0.892		1.277	*	0.782	*	1.144	
YebV	YP_490098.1	hypothetical protein Y75_p1812	0.853		0.592	*	1.799	*	1.504	
YecC	YP_490174.1	transporter subunit	0.841		0.939		1.112		1.23	

YecD	YP_490129.1	hydrolase	1.133		1.291		1.075		1.15	
YedD	YP_490185.1	hypothetical protein Y75_p1899	1.125		1.001		1.453		1.363	
YedO	YP_490176.1	D-cysteine desulfhydrase, PLP-dependent	1.001		1.076		1.33	*	1.391	*
YedP	YP_490208.1	hypothetical protein Y75_p1924	0.651		0.759		1.551		1.582	
YeeE	YP_490255.1	inner membrane protein	0.772		1.546		0.628	*	1.193	
YegP	YP_490322.1	hypothetical protein Y75_p2043	1.008		0.927		1.8		1.976	*
YfaW	YP_490486.1	enolase	0.971		1.442		0.718		0.837	
YfbE	YP_490492.1	uridine 5~-(beta-1-threo-pentapyranosyl-4-ulose diphosphate) aminotransferase, PLP-	2.67	*	1.55	*	1.246		0.94	
YfbF	YP_490493.1	undecaprenyl phosphate-L-Ara4FN transferase	2.608		0.759		2.659		0.766	
YfbG_2	YP_490494.1	fused UDP-L-Ara4N formyltransferase and UDP-GlcA C-4~-decarboxylase	1.248		1.571		1.173		1.115	
YfcB	YP_490572.1	N5-glutamine methyltransferase	0.995		1.483		0.659		0.922	
YfdH	YP_490592.1	bactoprenol glucosyl transferase	1.083		1.518	*	0.757	*	1.059	
YfeY	YP_490668.1	hypothetical protein Y75_p2393	1.295		1.288		1.131		1.283	*
YfgA	YP_490744.1	hypothetical protein Y75_p2469	1.202		1.04		1.026		0.916	

YfhO	YP_490758.1	cysteine desulfurase (tRNA sulfurtransferase), PLP-dependent	1.196	*	1.205		1.374		1.388	
YfhP	YP_490759.1	DNA-binding transcriptional activator	0.794		0.868		2.463	*	2.997	*
YfjB	YP_490837.1	NAD kinase	0.938		1.36		0.755		1.083	
YgaU	YP_490880.1	hypothetical protein Y75_p2608	1.597	*	1.374		2.21	*	1.678	*
YgdI	YP_491017.1	hypothetical protein Y75_p2746	1.031		1.222		1.436	*	1.684	*
YggE	YP_491122.1	hypothetical protein Y75_p2853	1.545	*	1.231	*	1.291		0.925	
YggG	YP_491135.1	peptidase	1.319	*	1.018		1.227		1.029	
YggH	YP_491159.1	tRNA (m7G46) methyltransferase, SAM-dependent	1.071		1.527	*	0.625	*	0.959	
YggJ	YP_491145.1	hypothetical protein Y75_p2876	1.215	*	0.846		1.545		1.065	
YggN	YP_491157.1	hypothetical protein Y75_p2888	1.351		1.037		1.132		0.952	
YghA	YP_491198.1	glutathionylspermidine synthase, with NAD(P)-binding Rossmann-fold domain	1.118		1.108		2.177		1.961	*
YghU	YP_491186.1	S-transferase	0.974		1.344	*	0.912		1.171	
YgiM	YP_491247.1	signal transduction protein	1.681	*	1.563		1.009		1.046	
YgiW	YP_491216.1	hypothetical protein Y75_p2950	2.484	*	3.357	*	1.621		1.994	

YgjF	YP_491260.1	G/U mismatch-specific DNA glycosylase	1.295	*	0.927		1.541		1.088	
YgjG	YP_491264.1	putrescine:2-oxoglutaric acid aminotransferase, PLP-dependent	0.895		0.685	*	3.325		2.291	
YhbN	YP_491385.1	transporter subunit	1.639		0.989		1.493		0.642	*
YhbZ	YP_491368.1	GTPase involved in cell partioning and DNA repair	0.961		1.293	*	0.854	*	1.012	
YhdR+P	YP_491429.1	membrane protein transporter	1.325		0.922		1.296		0.893	
YheO	YP_492086.1	DNA-binding transcriptional regulator	1.089		1.381		0.706	*	0.872	*
YhhW	YP_491995.1	hypothetical protein Y75_p3739	0.661	*	0.556	*	1.821	*	1.548	*
YhjA	YP_491917.1	cytochrome C peroxidase	1.012		1.217		0.794		0.945	
YhjG	YP_491911.1	outer membrane biogenesis protein	0.918		0.947		1.097		1.455	
YhjW	YP_491890.1	metal dependent hydrolase	1.856	*	1.261		1.039		0.835	
YibF	YP_491842.1	glutathione S-transferase	1.023		1.22	*	1.141		1.342	*
YibN	YP_491822.1	rhodanese-related sulfurtransferase	1.23	*	0.974		1.045		0.875	*
YibT	YP_491832.1	hypothetical protein Y75_p3573	1.014		0.929		1.528	*	1.212	
YidC	YP_491729.1	cytoplasmic insertase into membrane protein, Sec system	1.211		1.066		0.987		1.016	

YifE	YP_491672.1	hypothetical protein Y75_p3410	0.905		1.573	*	0.557	*	0.902	
YihD	YP_491591.1	hypothetical protein Y75_p3327	1.235	*	1.314	*	0.816	*	0.848	*
YihE	YP_491590.1	kinase	0.862		0.92		1.186		1.251	*
YihI	YP_491584.1	hypothetical protein Y75_p3320	1.385	*	1.432		0.839		0.965	
YiiD	YP_491562.1	acetyltransferase	1.002		1.098		0.943		1.286	
YiiT	YP_491528.1	stress-induced protein	0.85	*	1.216	*	0.652	*	0.991	
YijP	YP_491497.1	inner membrane protein	1.827	*	1.242		1.045		0.846	
YjbJ	YP_492188.1	stress response protein	1.199		0.8	*	2.792		1.914	*
YjdJ	YP_492270.1	acyltransferase	33.82		1.444		18.02		0.761	
YjeI	YP_492287.1	hypothetical protein Y75_p4031	2.451	*	1.25		0.793		0.332	*
YjgA	YP_492375.1	hypothetical protein Y75_p4119	0.924		1.546		0.637		1.054	
YjgB	YP_492407.1	alcohol dehydrogenase, Zn-dependent and NAD(P)-binding	1.115		1.078		1.582	*	1.598	*
YjgR	YP_492401.1	ATPase	0.868		0.881	*	1.348		1.28	
YjiY	YP_492485.1	inner membrane protein	1.607	*	1.105		0.984		0.862	

YnaF	YP_489644.1	stress-induced protein, ATP-binding protein	1.275		1.216		1.115	*	1.016	
YncB	YP_489714.1	oxidoreductase	0.862		1.085		1.188		1.464	*
YncE	YP_489717.1	hypothetical protein Y75_p1428	2.964	*	1.83	*	1.134		0.828	*
YneA	YP_489779.1	AI2 transporter	1.517	*	1.035		1.187		0.786	*
YneB	YP_489780.1	aldolase	1.303		1.125	*	0.959		0.941	
YnhD	YP_489944.1	component of SufBCD complex, ATP-binding component of ABC superfamily	2.602	*	1.115		1.633		0.85	
YnhE	YP_489945.1	component of SufBCD complex	1.323		1.373		1.191		1.223	
YoaC	YP_490071.1	hypothetical protein Y75_p1785	1.52	*	0.59		1.516	*	0.568	
YohF	YP_490376.1	oxidoreductase with NAD(P)-binding Rossmann-fold domain	0.989		1.053		1.439	*	1.516	*
YohN	YP_490347.1	hypothetical protein Y75_p2069	1.759		1.373		0.939		0.725	
YqeF	YP_491049.1	acyltransferase	1.537	*	1.252		1.213		1.087	
YqgF	YP_491148.1	Holliday junction resolvase	1.296		0.952		1.223		0.889	
YqhD	YP_491204.1	alcohol dehydrogenase, NAD(P)-dependent	1.084		1.282	*	0.872		0.984	
YqjD	YP_491289.1	hypothetical protein Y75_p3023	0.946		1.201	*	0.871		1.111	

YqjG	YP_491293.1	S-transferase	0.589	*	1.162		0.898		1.619	*
YqjH	YP_491261.1	siderophore interacting protein	1.885	*	1.197		1.286	*	0.886	
YqjI	YP_491262.1	transcriptional regulator	1.703	*	1.394	*	1.016		0.782	*
YraL	YP_491333.1	methyltransferase	1.246		1.284		0.978		0.988	
YraO	YP_491336.1	DnaA initiator-associating factor for replication initiation	1.4	*	0.886		1.538	*	1.075	
YraP	YP_491337.1	hypothetical protein Y75_p3072	1.289	*	1.007		1.066		1.016	
YrfF	YP_492034.1	inner membrane protein	0.793		1.519		0.854		1.618	
YsgA	YP_491612.1	hydrolase	0.925		1.316		0.973		1.255	*
YtfP	YP_492364.1	hypothetical protein Y75_p4108	1.782		1.085		0.994		0.599	
Zfba	YP_491125.1	fructose-bisphosphate aldolase, class II	1.119		1.446		0.77	*	1.011	
ZilvD	YP_491667.1	dihydroxyacid dehydratase	1.167		0.971		1.467		1.208	

Note: [a] 1-vs-2, 12 h with 0 mM versus 1 mM Mn (II); 3-vs-4, 48 h with 0 mM versus 1 mM Mn (II); 1-vs-3, 12 h versus 48 h with 0 mM Mn (II); and 2-vs-4, 12 h versus 48 h with 1 mM Mn (II). Red value denotes up-regulated expression (>1.2 fold); Green value denotes down-regulated (< 0.833 fold).

[b] Sig, significance.

[c] *, p -values < 0.05 and the up-regulated value >1.2 fold / down-regulated value < 0.833 fold.

Table S2. All of the down-regulated proteins identified by iTRAQ.[a–c]

Protein or	Accession	Desription	1-vs-2	Sig	3-vs-4	Sig	1-vs-3	Sig	2-vs-4	Sig
Aas	YP_491041.1	bifunctional 2-acylglycerophospho-ethanolamine acyl transferase and acyl-acyl	0.94		0.751		1.239		1.004	
AcnA	YP_489544.1	aconitate hydratase 1	0.752		0.815		1.045		1.117	
AefA	YP_488756.1	fused mechanosensitive channel proteins	0.823		0.691		0.954		0.793	
AldH	YP_489568.1	gamma-Glu-gamma-aminobutyraldehyde dehydrogenase, NAD(P)H-dependent	0.487		0.613	*	0.836	*	0.98	
Alr	YP_492196.1	alanine racemase 1, PLP-binding, biosynthetic	0.992		0.896		0.925		0.827	
Amn	YP_490228.1	AMP nucleosidase	0.888		0.76	*	1.044		0.941	
AmpD	YP_488413.1	N-acetyl-anhydromuranmyl-L-alanine amidase	0.792		0.88		1.083		1.192	
AnsB	YP_491156.1	periplasmic L-asparaginase II	1.01		0.622	*	1.099	*	0.643	
ApaG	YP_488356.1	protein associated with Co2+ and Mg2+ efflux	1.142		0.923		0.943		0.754	
AppA	YP_489251.1	phosphoanhydride phosphorylase	0.766		0.951		1.019		1.155	
ArcB	YP_491395.1	hybrid sensory histidine kinase in two-component regulatory system with ArcA	1.009		0.768	*	1.147	*	0.884	
AroE	YP_492151.1	dehydroshikimate reductase	0.783		0.891		1.253		0.976	

AroF	YP_490824.1	3-deoxy-D-arabino-heptulosonate-7-phosphate synthase, tyrosine-repressible	0.783		0.562		1.095		0.777	
AroK	YP_492042.1	shikimate kinase I	1.021		0.803		1.098		0.919	
AroL	YP_488681.1	shikimate kinase II	0.724		0.863		0.976		1.152	
AsmA	YP_490306.1	assembly protein	1.107		0.822		1.304	*	1.044	
AspA	YP_492282.1	aspartate ammonia-lyase	0.857	*	0.921		0.788		0.83	
AtpB	YP_491691.1	F0 sector of membrane-bound ATP synthase, subunit a	1.039		0.67		1.716	*	1.094	
AtpE	YP_491692.1	F0 sector of membrane-bound ATP synthase, subunit c	1.12		0.662	*	1.621	*	0.948	
BetA	YP_488606.1	choline dehydrogenase	0.878		0.7	*	1.341	*	0.986	
BtuB	YP_491486.1	vitamin B12/cobalamin outer membrane transporter	0.911		0.775		1.497	*	1.09	
CadA	YP_492275.1	lysine decarboxylase 1	1.092		0.966		0.793		0.694	
CelA	YP_489999.1	PTS system N,N~-diacetylchitobiose-specific transporter subunit IIB	1.188		1.135		0.724	*	0.771	*
ClpX	YP_488730.1	ATPase and-specificity subunit of ClpX-ClpP ATP-dependent serine protease	1.134		0.91		0.802		0.581	
Cls	YP_489517.1	cardiolipin synthase 1	1.174		0.979		1.571	*	1.06	
CoaA	YP_491482.1	pantothenate kinase	0.97		0.77		1.517		0.845	

CyaY	YP_491635.1	frataxin, iron-binding and oxidizing protein	0.943		0.824		1.148		0.986	
CydC	YP_489158.1	fused cysteine transporter subunits and membrane component and ATP-binding	1.064		0.655	*	1.514	*	0.822	
CydD	YP_489159.1	fused cysteine transporter subunits and membrane component and ATP-binding	1.157		0.549		1.833		0.726	
CysA	YP_490658.1	sulfate/thiosulfate transporter subunit	0.954		0.792		1.168		0.851	
CysN	YP_490960.1	sulfate adenylyltransferase, subunit 1	0.637	*	0.812	*	1.056		1.193	
CysP	YP_490661.1	thiosulfate transporter subunit	1.076		0.851	*	1.521	*	1.178	*
DadA	YP_489456.1	D-amino acid dehydrogenase	0.881		0.694	*	1.047		0.751	*
DapA	YP_490706.1	dihydrodipicolinate synthase	0.97		0.825	*	1.177	*	0.976	
DapE	YP_490699.1	N-succinyl-diaminopimelate deacylase	0.88		0.817		1.019		0.97	
DcuA	YP_492281.1	C4-dicarboxylate antiporter	0.786		0.749	*	1.143		1.088	
DdpA	YP_489752.1	D-Ala-D-Ala transporter subunit	1.007		0.648		1.349		0.859	
DeaD	YP_491349.1	ATP-dependent RNA helicase	0.831	*	0.471	*	0.474	*	0.268	*
DinG	YP_489072.1	ATP-dependent DNA helicase	1.173		0.776		1.687		1.105	*
DmsA	YP_489166.1	dimethyl sulfoxide reductase, anaerobic, subunit A	0.813	*	0.761	*	0.653	*	0.525	*

DmsB	YP_489167.1	dimethyl sulfoxide reductase, anaerobic, subunit B	1.173		0.712		0.923		0.487	*
DnaC	YP_492492.1	DNA biosynthesis protein	0.828		0.951		0.916		1.041	
DnaE	YP_488486.1	DNA polymerase III alpha subunit	1.107		0.819		1.215		0.823	
DnaJ	YP_488321.1	chaperone Hsp40, co-chaperone with DnaK	0.767	*	1.022		0.756	*	0.989	
DnaN	YP_491734.1	DNA polymerase III subunit beta	1.013		0.821	*	1.262	*	1.083	
DppD	YP_491894.1	dipeptide transporter	0.761	*	0.888		1.066		1.16	
DppF	YP_491895.1	dipeptide transporter	1.159		0.685	*	1.269		0.759	*
Dps	YP_489085.1	Fe-binding and storage protein	0.381		0.379	*	1.185	*	1.13	
DsdA	YP_490608.1	D-serine ammonia-lyase	0.714	*	0.646	*	0.977		0.901	
Dxr	YP_488475.1	1-deoxy-D-xylulose 5-phosphate reductoisomerase	0.719		0.839		0.886		1.002	
Era	YP_490794.1	membrane-associated, 16S rRNA-binding GTPase	0.803	*	0.87		0.835		0.815	
EvgA	YP_490611.1	DNA-binding response regulator in two-component regulatory system with EvgS	0.713	*	0.81	*	0.926		1.122	
FabR	YP_491489.1	DNA-binding transcriptional repressor	0.795		0.92		0.921		1.064	
FadR	YP_489454.1	DNA-binding transcriptional dual regulator	0.953		0.623	*	1.188		0.782	

FdnG	YP_489739.1	formate dehydrogenase-N subunit alpha, nitrate-inducible	0.745		0.89		0.724		0.856	
FimA	YP_492448.1	major type 1 subunit fimbrin	0.891		0.489		0.896	*	0.46	
FliY	YP_490177.1	cystine transporter subunit	1.027		0.804	*	1.474	*	1.115	*
folA	YP_488354.1	dihydrofolate reductase	0.73		0.583		0.866		0.857	
FolD	YP_488818.1	bifunctional 5,10-methylene-tetrahydrofolate dehydrogenase/5,10-	1.07		1.007		0.855		0.771	
FolP	YP_491362.1	7,8-dihydropteroate synthase	0.999		0.784	*	0.941		0.779	*
FrdA	YP_492299.1	fumarate reductase (anaerobic) catalytic and NAD/flavoprotein subunit	0.621		0.623		1.042		1.076	
FrdB	YP_492298.1	fumarate reductase (anaerobic), Fe-S subunit	0.894	*	0.566	*	1.138	*	0.742	*
Ftn	YP_490165.1	cytoplasmic ferritin iron storage protein	0.487	*	0.63		0.715	*	0.951	
FucO	YP_491007.1	L-1,2-propanediol oxidoreductase	0.895	*	0.761	*	1.152	*	0.99	
FucR	YP_491013.1	DNA-binding transcriptional activator	1.126		0.997		0.935		0.82	
FucU	YP_491012.1	L-fucose mutarotase	0.823		1.105		0.766		0.898	
FumB_1	YP_492265.1	anaerobic class I fumarate hydratase	0.575	*	0.649	*	0.93		0.986	
GadB	YP_489758.1	glutamate decarboxylase B, PLP-dependent	0.555		0.662		0.98		1.215	

GalE	YP_489032.1	UDP-galactose-4-epimerase	1.037		1		0.884		0.811	*
GalM	YP_489029.1	galactose-1-epimerase	1.026		0.755		1.081		0.731	
GalT	YP_489031.1	galactose-1-phosphate uridylyltransferase	1.071		0.733		1.148		0.702	
GatC	YP_490330.1	PTS system galactitol-specific transporter subunit IIC	1.071		0.815	*	0.692	*	0.539	*
Gcl	YP_488797.1	glyoxylate carboligase	0.481		0.648		0.856		1.141	
GcpE	YP_490743.1	1-hydroxy-2-methyl-2-(E)-butenyl 4-diphosphate synthase	0.939		0.8	*	0.873		0.799	*
GcvR	YP_490707.1	DNA-binding transcriptional repressor	0.981		0.829		1.313		1.098	
GlcB	YP_491172.1	malate synthase G	0.713		0.81		0.947		1.078	
GlcC	YP_491177.1	DNA-binding transcriptional dual regulator	0.881	*	0.827		0.72		0.712	
GlgA	YP_492004.1	glycogen synthase	1.153	*	0.812		1.405		1.081	
GlgC	YP_492003.1	glucose-1-phosphate adenylyltransferase	0.812		0.905		0.964		1.169	
Glk	YP_490630.1	glucokinase	0.822		0.991		0.954		1.004	
GlmU	YP_491699.1	fused N-acetyl glucosamine-1-phosphate uridyltransferase and glucosamine-1-	0.764	*	1.066		0.922		1.136	
GlnD	YP_488469.1	uridylyltransferase	1.036		0.772		1.265	*	0.983	

GlnL	YP_491581.1	sensory kinase in two-component regulatory system with GlnG	1.06		0.687		1.124		0.789	
GlpA	YP_490480.1	sn-glycerol-3-phosphate dehydrogenase (anaerobic), large subunit	0.609	*	0.379	*	0.996		0.8	
GlpD	YP_492007.1	sn-glycerol-3-phosphate dehydrogenase, aerobic	0.96		0.652	*	0.702	*	0.546	*
GlpK	YP_491525.1	glycerol kinase	0.706		0.773		0.904		0.954	
GlpQ	YP_490478.1	periplasmic glycerophosphodiester phosphodiesterase	0.822		0.532		1.041		0.64	
GlpR	YP_491566.1	DNA-binding transcriptional regulator	1.01		0.642		1.31		0.823	
GlpX	YP_491526.1	fructose 1,6-bisphosphatase II	0.771	*	0.687	*	0.88		0.926	
GltB	YP_491397.1	glutamate synthase, large subunit	0.922	*	0.833	*	0.875	*	0.83	
GltD	YP_491398.1	glutamate synthase, 4Fe-4S protein, small subunit	0.809	*	1.135		0.809		1.061	
GltL	YP_488943.1	glutamate and aspartate transporter subunit	1.07		0.739		1.108		0.757	
GntR	YP_491996.1	DNA-binding transcriptional repressor	0.799		0.961		0.914		1.098	
GoaG	YP_489570.1	GABA aminotransferase, PLP-dependent	0.665	*	0.603	*	0.905	*	0.866	*
GpmB	YP_492525.1	phosphoglyceromutase 2, co-factor independent	0.832	*	0.711		1.2	*	0.867	
GpmI	YP_491821.1	phosphoglycero mutase III, cofactor-independent	0.81	*	0.817	*	0.962		0.963	

GppA	YP_491660.1	guanosine pentaphosphatase/exopolyphosphatase	1.069		0.586	*	1.613	*	0.911	*
GreB	YP_492026.1	transcription elongation factor	0.874		0.667		1.18		0.891	
GuaC	YP_488408.1	GMP reductase	0.739	*	0.842		0.91		1.182	
HdfR	YP_491673.1	DNA-binding transcriptional regulator	0.977		0.619		1.367		0.857	
HelD	YP_489234.1	DNA helicase IV	0.761	*	0.815	*	1.022		1.108	
HemC	YP_491637.1	hydroxymethylbilane synthase	1.067		0.829		1.116		0.864	
HemD	YP_491638.1	uroporphyrinogen III synthase	0.955		0.827		1.256		0.883	
HemL	YP_488457.1	glutamate-1-semialdehyde aminotransferase	0.756	*	0.801	*	1.036		1.023	
HemM	YP_489476.1	chaperone for lipoproteins	0.893		0.713	*	1.288		1.024	
HemN	YP_491583.1	coproporphyrinogen III oxidase, SAM and NAD(P)H dependent, oxygen-independent	0.8		0.874		0.88		0.993	
HemX	YP_491639.1	uroporphyrinogen III methylase	0.977		0.831	*	1.337	*	1.088	
Hfq	YP_492314.1	HF-I, host factor for RNA phage Q beta replication	0.803	*	1.076		0.744	*	0.984	
HimA	YP_489974.1	integration host factor (IHF), DNA-binding protein subunit alpha	1.133	*	1.168		0.663	*	0.707	*
HisA	YP_490267.1	N-(5~-phospho-L-ribosyl-formimino)-5-amino-1- (5~-phosphoribosyl)-4-	1.095		0.546	*	1.465	*	0.84	

HisB_2	YP_490265.1	fused histidinol-phosphatase and imidazoleglycerol-phosphate dehydratase	0.79	*	0.936		0.982		1.193	*
HisC	YP_490264.1	histidinol-phosphate aminotransferase	0.756	*	0.86	*	0.84	*	0.983	
HisD	YP_490263.1	bifunctional histidinal dehydrogenase/histidinol dehydrogenase	0.809	*	0.792		1.096		1.135	*
HisF	YP_490268.1	imidazole glycerol phosphate synthase, catalytic subunit with HisF	0.859	*	0.802	*	1.086		1.021	
HisG	YP_490262.1	ATP phosphoribosyltransferase	0.696	*	0.778		0.85		1.015	
HisI_1	YP_490269.1	fused phosphoribosyl-AMP cyclohydrolase and phosphoribosyl-ATP pyrophosphatase	0.982		0.811		1.178	*	0.964	
HlyE	YP_489449.1	hemolysin E	1.01		0.96		0.861		0.81	
Hns	YP_489505.1	global DNA-binding transcriptional dual regulator H-NS	1.038		1.063		0.613		0.614	
HolA	YP_488931.1	DNA polymerase III subunit delta	0.808		0.58		1.748		1.241	
HsdM	YP_492480.1	DNA methylase M	0.868		0.897	*	0.901		0.809	*
HsdS	YP_492479.1	specificity determinant for hsdM and hsdR	0.809		0.785		1.278		0.776	
HtpX	YP_490091.1	endopeptidase	0.63	*	0.619		1.203		1.116	
HyaB	YP_489244.1	hydrogenase 1, large subunit	0.549		0.496	*	0.849	*	0.776	
HybA	YP_491193.1	hydrogenase 2 4Fe-4S ferredoxin-type component	0.789		0.657		0.932		0.767	

HybC	YP_491191.1	hydrogenase 2, large subunit	0.527	*	0.696		0.75		0.897	
HybO	YP_491194.1	hydrogenase 2, small subunit	0.783		0.447	*	1.086		0.613	
HycI	YP_490926.1	protease involved in processing C-terminal end of HycE	0.728		0.429		1.797		1.048	
HypB	YP_490936.1	GTP hydrolase involved in nickel liganding into hydrogenases	0.818		0.668	*	1.075		0.848	
IadA	YP_492462.1	isoaspartyl dipeptidase	0.86	*	0.7		1.395		1.077	
IlvA	YP_491666.1	threonine deaminase	1.189		0.754		1.124		0.759	
IlvB	YP_491763.1	acetolactate synthase I, large subunit	0.754	*	0.536	*	1.245	*	0.972	
IlvH	YP_488384.1	acetolactate synthase III, thiamin-dependent, small subunit	1.083		0.437		1.918	*	0.766	
InfC	YP_489980.1	protein chain initiation factor IF-3	1.132	*	1.055		0.815	*	0.831	
KdsB	YP_489190.1	3-deoxy-manno-octulosonate cytidylyltransferase	0.821	*	0.903		1.058		1.091	
KgtP	YP_490815.1	alpha-ketoglutarate transporter	0.932		0.817		1.142		1.043	
KsgA	YP_488357.1	S-adenosylmethionine-6-N~,N~-adenosyl (rRNA) dimethyltransferase	0.643		0.711		0.979		0.866	
LeuB	YP_488379.1	3-isopropylmalate dehydrogenase	0.682	*	0.671		1.168		0.818	
LexA	YP_492186.1	DNA-binding transcriptional repressor	1.086		0.852		0.83		0.717	*

Lgt	YP_491033.1	phosphatidylglycerol-prolipoprotein diacylglyceryl transferase	1.069		0.655	*	1.546	*	1.066	
Lhr	YP_489917.1	ATP-dependent helicase	1.165		0.835		1.173		0.832	
Lig	YP_490647.1	DNA ligase, NAD(+)-dependent	1.072		0.77	*	1.442	*	0.97	
LipA	YP_488919.1	lipoate synthase	0.932		0.79	*	0.764	*	0.692	
LipB	YP_488921.1	lipoyl-protein ligase	1.167		1.073		0.744		0.678	
Lnt	YP_488948.1	apolipoprotein N-acyltransferase	0.703		0.57		0.755		0.911	
LplA	YP_492516.1	lipoate-protein ligase A	0.861	*	0.714	*	1.213		1.098	
Lpp	YP_489939.1	murein lipoprotein	0.699	*	0.846		0.966		1.169	
LysC	YP_492167.1	aspartokinase III	0.745	*	0.799		1.146		1.088	
ManZ	YP_490080.1	PTS system mannose-specific transporter subunit IID	0.968		0.564		1.519		0.953	
MazG	YP_490989.1	nucleoside triphosphate pyrophosphohydrolase	1.121		0.818		1.566		1.131	*
McrB	YP_492477.1	5-methylcytosine-specific restriction enzyme McrBC, subunit McrB	0.747		0.771		0.86		0.878	
MdaA	YP_489124.1	nitroreductase A, NADPH-dependent, FMN-dependent	1.016		0.79	*	1.16		0.949	
MdaB	YP_491220.1	NADPH quinone reductase	0.93		0.82		0.971		0.876	

MelA	YP_492262.1	alpha-galactosidase	1.053		0.875		0.946		0.832	
MenB	YP_490502.1	dihydroxynaphthoic acid synthetase	0.812	*	0.894	*	1.01		1.154	*
MenD	YP_490504.1	bifunctional 2-oxoglutarate decarboxylase/SHCHC synthase	0.64		0.376		1.085		0.824	
MesJ	YP_488490.1	tRNA(Ile)-lysidine synthetase	0.766		0.704		1.145		1.041	
MglC	YP_490387.1	methyl-galactoside transporter subunit	0.917		0.89		0.733		0.704	
MhpR	YP_488640.1	DNA-binding transcriptional activator	0.807		0.882		1.062		1.117	
ModA	YP_489036.1	molybdate transporter subunit	0.913		0.524	*	1.885		1.036	
MoeA	YP_489100.1	molybdopterin biosynthesis protein	1.096		0.614	*	1.335	*	0.776	
Mog	YP_488315.1	molybdochelatase	1.007		0.749		1.16	*	0.989	
Mpl	YP_492374.1	UDP-N-acetylmuramate:L-alanyl-gamma-D-glutamyl- meso-diaminopimelate ligase	0.948		0.794	*	1.044		1.024	
MrcB	YP_488452.1	fused glycosyl transferase and transpeptidase	1.033		0.785		1.175		0.924	
MscL	YP_492142.1	mechanosensitive channel	0.989		0.737	*	1.192		0.838	
MsrA	YP_492361.1	methionine sulfoxide reductase A	1.042		0.702	*	1.37	*	1.045	
MukB	YP_489196.1	chromosome partitioning proteins	1.137	*	0.872		0.88		0.65	*

MukE	YP_489195.1	protein involved in chromosome partitioning	1.117		0.923		0.947		0.776	*
MurC	YP_488396.1	UDP-N-acetylmuramate:L-alanine ligase	0.99		0.703	*	1.226	*	0.832	
MurE	YP_488390.1	UDP-N-acetylmuramoyl-L-alanyl-D-glutamate:meso- diaminopimelate ligase	0.999		0.727	*	1.455		0.952	
MurG	YP_488395.1	N-acetylglucosaminyl transferase	0.892		0.583		1.625	*	1.214	
MutM	YP_491798.1	formamidopyrimidine/5-formyluracil/ 5-hydroxymethyluracil DNA glycosylase	1.074		0.552		1.309		0.666	
NanR	YP_491410.1	DNA-binding transcriptional dual regulator	0.769		0.955		0.797		0.936	
NapA	YP_490444.1	nitrate reductase, periplasmic, large subunit	0.694	*	0.618	*	0.57	*	0.498	*
NarK	YP_489493.1	nitrate/nitrite transporter	1.098		0.722		1.392		1.026	
NarP	YP_490431.1	DNA-binding response regulator in two-component regulatory system with NarQ or	0.908		0.858	*	0.74		0.773	*
NarZ	YP_489733.1	nitrate reductase 2 (NRZ) subunit alpha	0.817		0.729		0.891		0.788	
Ndk	YP_490746.1	multifunctional nucleoside diphosphate kinase, apyrimidinic endonuclease, and 3~-	1.089	*	0.827	*	1.098		0.865	
NemA	YP_489914.1	N-ethylmaleimide reductase, FMN-linked	0.794		0.944		0.694	*	0.989	
NirB	YP_492066.1	nitrite reductase, large subunit, NAD(P)H-binding	0.692	*	0.584	*	0.83		0.654	*
NlpB	YP_490705.1	lipoprotein	1.184	*	0.805	*	1.532	*	1.002	

NrdD	YP_492378.1	anaerobic ribonucleoside-triphosphate reductase	0.821	*	0.786	*	0.85	*	0.79	*
NrdE	YP_490890.1	ribonucleoside-diphosphate reductase 2 subunit alpha	1.15		0.688		1.629		0.964	
NrfA	YP_492213.1	nitrite reductase, formate-dependent, cytochrome	0.724	*	0.813		0.706	*	0.853	
NudE	YP_492035.1	ADP-ribose diphosphatase	1.132		0.773		1.392		1.067	
NuoB	YP_490527.1	NADH:ubiquinone oxidoreductase, chain B	1.172	*	0.924		1.062		0.817	*
NuoH	YP_490522.1	NADH:ubiquinone oxidoreductase, membrane subunit H	0.909		0.738	*	1.146	*	0.834	*
NupC	YP_490634.1	nucleoside (except guanosine) transporter	0.729		0.813		1.03		1.149	
NusG	YP_491479.1	transcription termination factor	1.081		0.98		0.87		0.82	
OmpT	YP_488852.1	outer membrane protease VII	0.323	*	0.298	*	1.298	*	1.143	
OsmY	YP_492505.1	hypothetical protein Y75_p4259	1.194	*	0.81	*	1.809	*	1.124	
PaaK	YP_489667.1	phenylacetyl-CoA ligase	1.114		0.929		1.059		0.787	
PcnB	YP_488446.1	poly(A) polymerase I	0.86		0.766	*	0.985		0.984	
PdhR	YP_488416.1	DNA-binding transcriptional dual regulator	0.623		0.612		1.125		1.082	
PdxH	YP_489902.1	pyridoxine 5~-phosphate oxidase	0.888	*	0.788	*	0.943		0.845	*

PepB	YP_490751.1	aminopeptidase B	0.973		0.748		1.207	*	1.003	
PepE	YP_492164.1	(alpha)-aspartyl dipeptidase	0.961		0.651		0.966		0.858	
PepQ	YP_491597.1	proline dipeptidase	0.778		1.01		0.888		1.047	
PepT	YP_489395.1	peptidase T	0.818		0.892	*	0.84	*	0.983	
PheS	YP_489976.1	phenylalanine tRNA synthetase subunit alpha	0.767	*	0.87	*	0.893	*	1.016	
PhoP	YP_489398.1	DNA-binding response regulator in two-component regulatory system with PhoQ	0.629	*	0.615	*	1.097		1.022	
PotF	YP_489127.1	putrescine transporter subunit	1.149		0.802		1.566		1.082	
Ppa	YP_492368.1	inorganic pyrophosphatase	1.038		0.964		0.9	*	0.813	*
PrfA	YP_489478.1	peptide chain release factor RF-1	0.774		0.757	*	1.215		1.033	
PriA	YP_491516.1	primosome factor n~	0.71		0.813		0.942		1.066	
PrkB	YP_492076.1	phosphoribulokinase	0.702		0.88	*	0.941		1.019	
PrmA	YP_491441.1	methylase for 50S ribosomal protein L11	1.081		0.605		1.515	*	1.032	
ProQ	YP_490093.1	structural transport element	0.89		0.978		0.653		0.789	
PrpB	YP_488626.1	2-methylisocitrate lyase	1.087		0.682	*	5.738	*	3.627	

PrpC	YP_488627.1	2-methylcitrate synthase	0.815		0.566	*	5.056		3.659	
PrpD	YP_488628.1	2-methylcitrate dehydratase	0.778		0.665	*	7.08		3.758	
Pth	YP_489471.1	peptidyl-tRNA hydrolase	1.023		0.995		0.921		0.83	
PtsA_1	YP_491504.1	fused PTS enzymes Hpr component, enzyme I component, and enzyme IIA	1.077		0.832	*	1.261	*	1.009	
PurB	YP_489399.1	adenylosuccinate lyase	0.817		0.94		0.959		1.033	
PurC	YP_490704.1	phosphoribosylaminoimidazole-succinocarboxamide synthetase	0.727	*	1.11		0.8	*	1.165	*
PurD	YP_491455.1	phosphoribosylglycinamide synthetase phosphoribosylamine-glycine ligase	0.766	*	0.848		1.109		1.169	
PurE	YP_488812.1	N5-carboxyaminoimidazole ribonucleotide mutase	0.858		0.548		1.314		1.224	*
PurK	YP_488811.1	N5-carboxyaminoimidazole ribonucleotide synthase	0.76		0.985		0.908		1.165	
PurL	YP_490785.1	phosphoribosylformyl-glycineamide synthetase	0.805		0.813		1.1		1.159	
PurT	YP_490111.1	phosphoribosylglycinamide formyltransferase 2	0.585		0.728	*	1.199		1.647	*
PyrB	YP_492385.1	aspartate carbamoyltransferase, catalytic subunit	0.683		0.764		0.881	*	0.948	
PyrC	YP_489330.1	dihydro-orotase	0.881	*	0.814		1.116		0.98	
PyrD	YP_489217.1	dihydro-orotate oxidase, FMN-linked	0.936		0.832	*	1.041		0.968	

PyrG	YP_490988.1	CTP synthetase	0.81	*	0.941		0.745		0.9	
PyrI	YP_492384.1	aspartate carbamoyltransferase, regulatory subunit	0.718	*	0.818		0.945		0.931	*
RbfA	YP_491354.1	30s ribosome binding factor	0.987		0.791	*	0.975		0.811	*
RbsK	YP_491677.1	ribokinase	1.174		0.826		1.117		0.778	
RecC	YP_491027.1	exonuclease V (RecBCD complex) subunit gamma	0.954		0.768		1.089	*	0.869	
RffG	YP_491651.1	dTDP-glucose 4,6-dehydratase	0.928		0.736		0.892		0.7	
RhlE	YP_489070.1	RNA helicase	0.648	*	0.597	*	1.108		0.971	
RibA	YP_489545.1	GTP cyclohydrolase II	0.819	*	0.873		1.106		1.101	
RibD_2	YP_488706.1	fused diaminohydroxyphosphoribosylaminopyrim	1.041		0.781	*	1.126		0.908	
RluC	YP_489354.1	23S rRNA pseudouridylate synthase	0.789		0.887		0.992		1.104	
Rmf	YP_489225.1	ribosome modulation factor	0.633	*	1.192	*	0.348	*	0.836	
Rnd	YP_490065.1	ribonuclease D	0.801		0.775	*	1.024		0.991	
Rnk	YP_488899.1	regulator of nucleoside diphosphate kinase	0.817		0.736		1.344	*	1.197	
Rpe	YP_492046.1	D-ribulose-5-phosphate 3-epimerase	1.064		0.82	*	1.286	*	0.987	

RplA	YP_491477.1	50S ribosomal protein L1	1.035		1.099		0.833		0.815	
RplD	YP_492113.1	50S ribosomal protein L4	0.825	*	0.975		0.973		1.173	
RpsI	YP_491414.1	30S ribosomal protein S9	0.803		1.12	*	0.811		1.053	
RpsK	YP_492135.1	30S ribosomal protein S11	0.797	*	0.991		0.918		1.089	
RpsP	YP_490832.1	30S ribosomal protein S16	1.007		1.026		0.613		0.679	
RpsQ	YP_492121.1	30S ribosomal protein S17	0.782		1.067		0.909		1.11	
RssA	YP_489502.1	hypothetical protein Y75_p1207	1.115		0.457		1.223		0.555	
RuvA	YP_490123.1	component of RuvABC resolvasome, regulatory subunit	0.881		0.728		1.107		0.797	
RuvB	YP_490122.1	ATP-dependent DNA helicase, component of RuvABC resolvasome	0.828	*	0.941		0.675		0.679	*
SbcB	YP_490253.1	exonuclease I	0.731		0.823		0.995		1.109	
SdaA	YP_490075.1	L-serine deaminase I	0.78	*	0.907		0.83		0.931	
SdhA	YP_489003.1	succinate dehydrogenase, flavoprotein subunit	0.992		0.785		1.237		0.954	
SdhB	YP_489004.1	succinate dehydrogenase, FeS subunit	0.96		0.773	*	1.189		1.006	
SdhC	YP_489001.1	succinate dehydrogenase, membrane subunit, binds cytochrome b556	0.937		0.654		1.559	*	0.853	

SelA	YP_491843.1	selenocysteine synthase	0.746	*	0.85	*	0.938		1.027	
SelB	YP_491844.1	selenocysteinyl-tRNA-specific translation factor	0.806		1.028		0.926		1.136	
SerS	YP_489165.1	seryl-tRNA synthetase, also charges selenocysteinyl-tRNA with serine	0.824	*	0.931		0.845	*	1.029	
SfcA	YP_489744.1	malate dehydrogenase, NAD-requiring	0.715		0.816	*	1.036		1.155	*
SgaH	YP_492338.1	3-keto-L-gulonate 6-phosphate decarboxylase	0.62		0.377	*	1.145		0.793	
Slp	YP_491929.1	outer membrane lipoprotein	0.542		0.382	*	1.306		0.905	
Slt	YP_492522.1	lytic murein transglycosylase, soluble	0.78		0.865	*	1.191		1.156	
SlyD	YP_492083.1	FKBP-type peptidyl prolyl cis-trans isomerase	1.071		0.824	*	1.332	*	1.083	
SpeC	YP_491164.1	ornithine decarboxylase	0.894		0.398		1.114		0.543	
SpeG	YP_489847.1	spermidine N1-acetyltransferase	1.106		0.717		0.992		0.775	
SpoU	YP_491783.1	tRNA (Guanosine-2~-O-)-methyltransferase	0.738		0.922		0.94		1.142	
SseA	YP_490749.1	3-mercaptopyruvate sulfurtransferase	0.973		0.687		1.479	*	1.097	*
SsnA	YP_491081.1	chlorohydrolase/aminohydrolase	0.511		0.741	*	0.61		0.935	
SspA	YP_491413.1	stringent starvation protein A	0.814	*	0.88	*	0.944		1.027	

SufI	YP_491209.1	repressor protein for FtsI	1.021		0.684		1.411		0.92	
Syd	YP_491001.1	hypothetical protein Y75_p2730	0.766		0.834		1.305		1.146	
TatD	YP_491603.1	DNase, magnesium-dependent	1.147		0.751		1.221	*	0.961	
TdcE	YP_491303.1	pyruvate formate-lyase 4/2-ketobutyrate formate-lyase	0.95		0.853		0.822	*	0.786	*
TdcG	YP_491301.1	L-serine dehydratase 3	0.854		0.817		0.95		0.741	*
ThdF	YP_491728.1	GTPase	0.862	*	0.737	*	0.88		0.768	
ThrA	YP_488308.1	fused aspartokinase I and homoserine dehydrogenase I	0.69		0.83	*	0.927		1.028	
ThrS	YP_489981.1	threonyl-tRNA synthetase	0.802		0.931		0.844		1.049	
TreB	YP_492380.1	PTS system fused trehalose(maltose)-specific transporter subunit IIBC	0.878		0.777		0.74		0.668	
TreC	YP_492379.1	trehalose-6-P hydrolase	0.726	*	0.749		0.688		0.774	*
TrmA	YP_491487.1	tRNA (uracil-5-)-methyltransferase	0.766		0.88		0.83		0.944	
TrmC	YP_490566.1	bifunctional 5-methylaminomethyl-2-thiouridine forming enzyme	0.817		1.076		0.821		0.943	
UbiC	YP_492182.1	chorismate pyruvate lyase	0.837		0.48		1.379		0.783	
UbiF	YP_488953.1	2-octaprenyl-3-methyl-6-methoxy-1,4-benzoquinol oxygenase	0.904		0.826	*	1.089		0.934	

UbiG	YP_490471.1	bifunctional 3-demethylubiquinone-9 3-methyltransferase/2-octaprenyl-6-hydroxy	0.938		1.082		0.721	*	0.768	*
UbiH	YP_491108.1	2-octaprenyl-6-methoxyphenol hydroxylase	0.757	*	0.902		1.036		0.978	
UgpB	YP_491981.1	glycerol-3-phosphate transporter subunit	0.878		0.698		1.386		1.091	
UvrA	YP_492201.1	ATPase and DNA damage recognition protein of nucleotide excision repair	0.823	*	0.831	*	1.009		1.056	
UvrB	YP_489052.1	excinulease of nucleotide excision repair, DNA damage recognition component	1.089		0.88		0.981		0.819	*
UvrY	YP_490171.1	DNA-binding response regulator in two-component regulatory system with BarA	0.968		0.822		1.027		0.737	*
UxaB	YP_489784.1	altronate oxidoreductase	0.906		0.817	*	1.125		0.906	
VisC	YP_491107.1	oxidoreductase	0.769	*	0.665	*	1.071		0.664	*
WrbA	YP_489277.1	flavoprotein in Trp regulation	0.909	*	0.791	*	1.237		1.037	
XdhD	YP_491083.1	fused xanthine/hypoxanthine oxidase, molybdopterin-binding subunit and Fe-S	0.584		0.74		0.775		0.957	
XseA	YP_490737.1	exonuclease VII, large subunit	1.056		0.715	*	1.089		0.916	
XthA	YP_490010.1	exonuclease III	1.152		0.99		1.049		0.74	*
YaaA	YP_488312.1	hypothetical protein Y75_p0006	0.996		0.979		0.911		0.828	
YabC	YP_488387.1	SAM-dependent methyltransferase	1.106		0.979		0.929		0.816	*

YafB	YP_488504.1	2,5-diketo-D-gluconate reductase B	0.81		0.65		1.333	*	0.913
YagD	YP_488556.1	S-methylmethionine:homocysteine methyltransferase	0.995		0.54	*	1.046		0.562
YaiI	YP_488680.1	hypothetical protein Y75_p0376	0.993		0.638	*	1.421		0.885
YaiM	YP_488649.1	esterase	1.087		0.527		1.367		0.943
YbaL	YP_488769.1	transporter with NAD(P)-binding Rossmann-fold domain	0.863		0.765	*	1.183	*	1.044
YbbK	YP_488780.1	protease, membrane anchored	0.909		0.825	*	1.082		1.015
YbbO	YP_488784.1	oxidoreductase with NAD(P)-binding Rossmann-fold domain	0.772		0.533		1.533		1.155
YbbX	YP_488802.1	allantoinase	0.431	*	0.581		0.96		1.121
YbdQ	YP_488897.1	universal stress protein UP12	0.764		0.858	*	0.943		1.121
YbeA	YP_488927.1	hypothetical protein Y75_p0626	0.742		0.971		0.954		1.058
YbeX	YP_488949.1	ion transport protein	0.75	*	0.734	*	0.894		0.995
YbgJ	YP_488991.1	hypothetical protein Y75_p0691	0.756		0.8		1.1		1.153
YbhC	YP_489045.1	pectinesterase	0.95		0.701		1.812		1.14
YbhE	YP_489040.1	6-phosphogluconolactonase	1.13		0.783	*	1.451	*	0.996

YbhG	YP_489068.1	membrane fusion protein (MFP) component of efflux pump, membrane	1.053		0.767	*	1.396	*	1.029	
YbhK	YP_489053.1	transferase with NAD(P)-binding Rossmann-fold domain	0.814		0.659		1.104		0.884	
YbiK	YP_489101.1	L-asparaginase	0.858		0.635		1.425		1.044	
YbiQ	YP_489090.1	transcriptional regulator of mntH	1.164		0.779		1.112		0.736	
YbjQ	YP_489139.1	hypothetical protein Y75_p0839	0.985		0.805	*	1.543	*	1.186	*
YbjV	YP_489145.1	HCP oxidoreductase	1.153		0.885		0.755		0.573	
YbjW	YP_489146.1	hybrid-cluster [4Fe-2S-2O] protein in anaerobic terminal reductases	1.006		0.797		1.043		0.583	*
YcbZ	YP_489227.1	peptidase	1.02		0.756		1.381		1.037	
YccU	YP_489237.1	CoA-binding protein with NAD(P)-binding Rossmann-fold domain	1.028		0.818		1.372	*	1.103	
YcdX	YP_489301.1	zinc-binding hydrolase	0.701	*	0.923		1.018		1.172	*
YcdY	YP_489302.1	hypothetical protein Y75_p1004	0.934		0.791	*	1.267	*	1.062	
YceD	YP_489356.1	hypothetical protein Y75_p1058	1.037		0.743		1.096		1.011	
YcfC	YP_489400.1	lysogenization regulator	0.895		0.678	*	1.406	*	0.9	
YcfD	YP_489396.1	hypothetical protein Y75_p1098	0.868	*	0.813	*	0.958		0.906	

YcfH	YP_489368.1	metallodependent hydrolase	1.166		0.796		1.211		0.818	
YcfM	YP_489373.1	outer membrane lipoprotein	1.118		0.768		1.689	*	1.084	
YcfY	YP_489388.1	deacetylase of acetyl-CoA synthetase	0.857		0.744		0.821		0.857	
YcgE	YP_489429.1	DNA-binding transcriptional regulator	0.766		0.579		1.333		0.993	
YcgL	YP_489446.1	hypothetical protein Y75_p1151	1.056		0.638		1.204		0.727	*
YcgS	YP_489466.1	dihydroxyacetone kinase, C-terminal domain	1.042		0.816		1.467	*	1.049	
YciM	YP_489548.1	hypothetical protein Y75_p1255	0.83		0.583		1.444		0.724	
YcjG	YP_489594.1	L-Ala-D/L-Glu epimerase	1.04		0.77		1.112		0.804	
YcjI	YP_489595.1	murein peptide amidase A	0.839		0.592		1.123		0.784	
YcjK	YP_489565.1	gamma-Glu-putrescine synthase	0.655	*	0.762	*	0.67	*	0.774	*
YcjL	YP_489566.1	gamma-Glu-GABA hydrolase	1.017		0.61	*	1.548	*	1.126	
YcjX	YP_489590.1	hypothetical protein Y75_p1297	0.877		0.771		1.119		0.974	
YdaO	YP_489614.1	C32 tRNA thiolase	1.114		0.625		1.465		0.814	
YdbU	YP_489664.1	3-hydroxybutyryl-CoA dehydrogenase	1.147		0.75		1.285		0.972	

YdcG	YP_489690.1	glucan biosynthesis protein, periplasmic	0.817	*	0.846	*	0.954		0.955	
YdcI	YP_489688.1	DNA-binding transcriptional regulator	1.067		0.776	*	0.768	*	0.571	*
YdcP	YP_489700.1	peptidase	0.729		0.589		0.812		0.648	
YdhH	YP_489904.1	hypothetical protein Y75_p1617	1.053		0.796		1.127	*	0.983	
YdhV	YP_489935.1	oxidoreductase	0.419		0.62		0.652		0.955	
YdjN	YP_489990.1	transporter	0.973		0.772	*	1.227		0.972	
YeaK	YP_490048.1	hypothetical protein Y75_p1762	0.912		0.808	*	1.091		1.066	
YeaO	YP_490053.1	hypothetical protein Y75_p1767	1.072		1		0.84	*	0.717	
YebE	YP_490108.1	hypothetical protein Y75_p1822	0.848		0.842		1.534	*	1.133	
YebK	YP_490115.1	DNA-binding transcriptional regulator	1.065		0.77		1.283		0.851	
YebT	YP_490096.1	hypothetical protein Y75_p1810	0.931		0.782		1.164		1.172	
YebV	YP_490098.1	hypothetical protein Y75_p1812	0.853		0.592	*	1.799	*	1.504	
YecO	YP_490132.1	methyltransferase	0.791	*	0.83		0.901		0.906	
YecP	YP_490133.1	methyltransferase	1.015		0.566		1.411		0.785	*

YeeI	YP_490225.1	hypothetical protein Y75_p1942	1.098		0.889		0.843		0.672	*
YeeN	YP_490229.1	hypothetical protein Y75_p1947	0.786		0.969		0.694	*	0.941	
YehA	YP_490348.1	fimbrial-like adhesin protein	0.761		0.324	*	1.862	*	0.785	
YehS	YP_490362.1	hypothetical protein Y75_p2085	0.897		0.762		1.22		1.027	
YeiA_1	YP_490386.1	oxidoreductase	0.564	*	0.759	*	0.573	*	0.725	*
YeiE	YP_490396.1	DNA-binding transcriptional regulator	1.082		0.664		1.649	*	0.772	
YeiG	YP_490393.1	esterase	1.002		0.82		1.282		1.043	
YeiN	YP_490404.1	hypothetical protein Y75_p2127	0.871		0.781		1.025		0.91	
YeiQ	YP_490412.1	dehydrogenase	1.112		0.685		1.154		0.732	
YeiR	YP_490413.1	hypothetical protein Y75_p2136	0.95		0.698	*	0.967		0.796	
YeiT	YP_490385.1	oxidoreductase	0.604	*	0.474	*	0.709		0.539	*
YfaO	YP_490490.1	NUDIX hydrolase	1.058		0.718		1.533		1.03	*
YfaY	YP_490488.1	hypothetical protein Y75_p2212	0.885		0.832	*	1.228	*	1.166	
YfbT	YP_490535.1	hydrolase or phosphatase	0.947		0.56		1.431		0.776	

YfcF	YP_490543.1	hypothetical protein Y75_p2267	1.029		0.529		1.501		0.764	
YfcX	YP_490583.1	fused enoyl-CoA hydratase, 3-hydroxybutyryl-CoA epimerase,	1.147		0.789		1.21		0.997	
YfcZ	YP_490585.1	hypothetical protein Y75_p2309	0.856	*	0.999		0.783	*	0.751	
YfdZ	YP_490621.1	aminotransferase, PLP-dependent	1.005		0.757	*	1.249		0.947	
YffH	YP_490694.1	NUDIX hydrolase	0.856		0.891		0.74		0.783	*
YfgB	YP_490745.1	hypothetical protein Y75_p2470	0.987		0.849		0.919		0.768	*
YfhQ	YP_490760.1	methyltransferase	0.921		0.772	*	1.007		0.985	
YfiD	YP_490807.1	pyruvate formate lyase subunit	1.081		0.853		0.758	*	0.548	
YfiH	YP_490817.1	hypothetical protein Y75_p2542	0.845		0.595		1.454		1.096	
YfiQ_1	YP_490812.1	fused acyl-CoA synthetase NAD(P)-binding subunit and ATP-binding subunit	0.888	*	0.783	*	1.187	*	1.02	
YgbB	YP_490955.1	2C-methyl-D-erythritol 2,4-cyclodiphosphate synthase	1.129		0.655	*	0.975		0.662	
YgbI	YP_490944.1	DNA-binding transcriptional regulator	0.852		0.827		1.053		1.056	
YgbO	YP_490954.1	pseudoruidine synthase	0.814		0.919		0.882		1.029	
YgcF	YP_490985.1	hypothetical protein Y75_p2714	0.527		1.001		0.882		1.439	

YgcP	YP_490977.1	anti-terminator regulatory protein	0.808		0.961		0.496		0.584	
YgcX	YP_490995.1	(D)-glucarate dehydratase 1	0.735		0.698		1.117		1.05	
YgdR	YP_491038.1	hypothetical protein Y75_p2767	1.121		0.675	*	1.158		0.727	
YgeA	YP_491045.1	racemase	0.924		0.545		2.361		1.378	*
YgeS	YP_491068.1	xanthine dehydrogenase, molybdenum binding subunit	0.655	*	0.768	*	0.997		1.034	
YgeT	YP_491069.1	xanthine dehydrogenase, FAD-binding subunit	0.829	*	0.821		0.779		0.777	*
YgeU	YP_491070.1	xanthine dehydrogenase, Fe-S binding subunit	0.793		1.013		0.614		0.776	
YgeW	YP_491072.1	hypothetical protein Y75_p2803	0.534		0.888		0.592		0.996	
YgeX	YP_491073.1	2,3-diaminopropionate ammonia-lyase	0.535		0.8	*	0.904		0.924	
YgeY	YP_491074.1	peptidase	0.55		0.79		0.684		0.924	
YgfK_2	YP_491080.1	oxidoreductase, Fe-S subunit	0.453		0.533		0.384		0.425	
YgfM	YP_491082.1	oxidoreductase	0.621	*	0.688	*	0.751		0.911	
YgfP	YP_491085.1	guanine deaminase	0.455	*	0.693		0.682	*	0.952	
YggB	YP_491124.1	mechanosensitive channel	1.084		0.698	*	1.726		1.038	

YggX	YP_491161.1	protein that protects iron-sulfur proteins against oxidative damage	1.07		1.095		0.657	*	0.829	
YgiC	YP_491230.1	hypothetical protein Y75_p2964	0.836	*	0.829	*	0.938		0.955	
YgiS	YP_491212.1	transporter subunit	0.81		0.972		0.779	*	0.912	
YgjO	YP_491275.1	methyltransferase small domain	0.743		0.781		0.824		0.857	
YhaE	YP_491313.1	tartronate semialdehyde reductase	0.806	*	0.757	*	1.035		0.974	
YhaF	YP_491314.1	alpha-dehydro-beta-deoxy-D-glucarate aldolase	1.035		0.832		1.132		1.176	
YhaG	YP_491316.1	(D)-galactarate dehydrogenase	0.761	*	0.571		1.171		0.952	
YhaR	YP_491302.1	L-PSP (mRNA) endoribonuclease	1.003		0.79	*	1.281	*	1.007	
YhbG	YP_491386.1	transporter subunit	1.02		0.748	*	1.177	*	0.901	
YhbS	YP_491343.1	acyltransferase	0.925		0.759	*	1.165	*	0.968	
YhbW	YP_491347.1	hypothetical protein Y75_p3082	1.082		0.738	*	1.137		0.836	*
YhcB	YP_491417.1	hypothetical protein Y75_p3153	0.994		0.696	*	1.334	*	0.893	*
YhfK	YP_492073.1	inner membrane protein	1.153		0.635		2.048		1.117	
YhhJ	YP_491949.1	transporter subunit	0.808		0.748		1.23		1.129	

YhhK	YP_491975.1	hypothetical protein Y75_p3719	0.786	*	0.813		1.061		1.097	
YhhW	YP_491995.1	hypothetical protein Y75_p3739	0.661	*	0.556	*	1.821	*	1.548	*
YhiH_1	YP_491948.1	fused ribosome-associated ATPases	0.893		0.696	*	1.439	*	1.128	
YhiI	YP_491947.1	HlyD family secretion protein	0.857		0.801		1.084		1.016	
YhiR	YP_491936.1	DNA (exogenous) processing protein	0.998		0.552		1.132		0.72	
YiaF	YP_491882.1	hypothetical protein Y75_p3623	0.915		0.827	*	1.31	*	1.172	*
YiaJ	YP_491860.1	DNA-binding transcriptional repressor	1.147		0.542	*	1.741	*	0.874	
YibL	YP_491831.1	hypothetical protein Y75_p3572	0.915		0.816		1.102		0.86	
YidR	YP_491746.1	hypothetical protein Y75_p3484	0.661	*	0.745		0.996		0.949	
YieF	YP_491716.1	chromate reductase, Class I, flavoprotein	1.036		0.831	*	1.183	*	0.942	
YieP	YP_491674.1	transcriptional regulator	0.771	*	0.953		0.943		1.04	
YigL	YP_491616.1	hydrolase	1.161		0.819		1.288	*	0.873	
YihX	YP_491565.1	hydrolase	0.721		0.85		1.005		1.151	
YiiU	YP_491523.1	hypothetical protein Y75_p3259	0.843		0.592	*	0.896		0.803	

YjaG	YP_491461.1	hypothetical protein Y75_p3197	0.757	*	1.038		0.918		0.979	
YjcG	YP_492210.1	acetate transporter	0.815		0.582	*	1.906		1.25	
YjdC	YP_492278.1	transcriptional regulator	1.015		0.863		1.528	*	1.136	
YjgF	YP_492383.1	ketoacid-binding protein	1.105	*	0.821	*	1.291	*	0.935	
YjhP	YP_492439.1	methyltransferase	0.736		0.978		1.047		1.084	
YjiE	YP_492461.1	DNA-binding transcriptional regulator	0.953		0.418		1.396		0.606	
YjiH	YP_492464.1	inner membrane protein	1.131		0.262	*	1.77	*	0.406	
YjiL	YP_492468.1	ATPase, activator of (R)-hydroxyglutaryl-CoA dehydratase	1.064		0.65		1.558		0.942	
YjiM	YP_492469.1	2-hydroxyglutaryl-CoA dehydratase	0.947		0.691	*	0.521	*	0.393	*
YjjI	YP_492510.1	hypothetical protein Y75_p4264	0.688	*	0.739	*	0.852	*	0.888	
YkgE	YP_488602.1	oxidoreductase	0.536	*	0.853		0.552	*	0.861	
YkgF	YP_488603.1	amino acid dehydrogenase	0.552	*	0.424		0.668	*	0.552	
YkgG	YP_488604.1	transporter	0.556		0.585		0.806		0.93	
YlaD	YP_488750.1	maltose O-acetyltransferase	0.814		0.52		1.313		0.83	

YneC	YP_489781.1	hypothetical protein Y75_p1493	1.062		0.863		1.403		0.773	
YneH	YP_489787.1	glutaminase	0.772	*	0.775		1.044		1.115	
YneI	YP_489788.1	aldehyde dehydrogenase	0.87		0.78	*	1.18	*	1.048	
YnfE	YP_489850.1	oxidoreductase subunit	0.754	*	0.668	*	0.819		0.661	*
YnfF	YP_489851.1	oxidoreductase subunit	0.686	*	0.961		0.453	*	0.62	
YniC	YP_489988.1	hydrolase	0.858	*	0.794	*	0.928		0.816	*
YnjB	YP_490015.1	hypothetical protein Y75_p1729	0.978		0.664		1.278	*	0.637	
YnjE	YP_490018.1	thiosulfate sulfur transferase	0.821	*	0.866		0.918		0.906	*
YoaE_2	YP_490077.1	membrane protein	0.831	*	0.592	*	1.238		0.883	
YojH	YP_490448.1	malate dehydrogenase domain	0.847		0.934		0.272	*	0.297	
YpfH	YP_490701.1	hydrolase	1.07		0.524		1.337	*	0.968	
YqeA	YP_491076.1	amino acid kinase	0.696	*	1.075		0.794		1.073	
YqiC	YP_491234.1	hypothetical protein Y75_p2968	1.066		0.805		0.951		0.75	*
YqjG	YP_491293.1	S-transferase	0.589	*	1.162		0.898		1.619	*

YrbD	YP_491378.1	ABC-type organic solvent transporter	0.778	*	0.735	*	1.044		0.928
YrdA	YP_492153.1	hypothetical protein Y75_p3897	1.002		0.689		1.339		0.929
YrfG	YP_492033.1	hydrolase	1.011		0.954		0.843		0.787
YtfB	YP_492348.1	cell envelope opacity-associated protein	1.051		0.821	*	0.99		0.762
YtfE	YP_492351.1	regulator of cell morphogenesis and cell wall metabolism	0.663		1.002		0.741		1.109
YtfG	YP_492353.1	NAD(P)H:quinone oxidoreductase	1.138		0.731	*	1.659	*	1.041
ZipA	YP_490648.1	cell division protein involved in Z ring assembly	0.88		0.359		1.46		0.552
Zkup	YP_491682.1	potassium transporter	1.189		0.69	*	1.427		0.82
Znfi	YP_491462.1	endonuclease V	0.92		0.639		1.223		0.84
ZntA	YP_491965.1	zinc, cobalt and lead efflux system	0.843		0.788		1.013		0.975

Note: [a] 1-vs-2, 12 h with 0 mM versus 1 mM Mn (II); 3-vs-4, 48 h with 0 mM versus 1 mM Mn (II); 1-vs-3, 12 h versus 48 h with 0 mM Mn (II); and 2-vs-4, 12 h versus 48 h with 1 mM Mn (II). Red value denotes up-regulated expression (>1.2 fold); Green value denotes down-regulated (< 0.833 fold).

[b] Sig, significance.

[c] *, p -values < 0.05 and the down-regulated value < 0.833 fold.

Table S3. Up- or down-regulated proteins related to DNA replication, transcription and translation.[a−c]

Protein	Accession	Desription	1-VS-2	Sig	3-VS-4	Sig	1-VS-3	Sig	2-VS-4	Sig
**DNA replication**										
DNA polymerase III										
DnaN	YP_491734.1	DNA polymerase III subunit beta	1.013		0.821	*	1.262	*	1.083	
DnaX	YP_488761.1	DNA polymerase III/DNA elongation factor III, tau and gamma subunits	1.005		0.884		1.183		1.127	
DnaE	YP_488486.1	DNA polymerase III alpha subunit	1.107		0.819		1.215		0.823	
HolC	YP_492397.1	DNA polymerase III, chi subunit	---		---		---		---	
HolA	YP_488931.1	DNA polymerase III subunit delta	0.808		0.58		1.748		1.241	
Other DNA polymerase										
PolA_2	YP_491586.1	fused DNA polymerase I 5~->3~ exonuclease, 3~->5~ polymerase and 3~->5~ exonuclease	0.902		0.993		0.927		1.066	
Rnt	YP_489916.1	ribonuclease T (RNase T)	1.149		1.079		1.014		0.942	
PolB	YP_488366.1	DNA polymerase II	---		---		---		---	

Replication initiation

SeqA	YP_488967.1	regulatory protein for replication initiation	0.983	0.99	0.954	0.936
YraO	YP_491336.1	DnaA initiator-associating factor for replication initiation	1.4 *	0.886	1.538 *	1.075
DnaA	YP_491733.1	chromosomal replication initiator protein DnaA, DNA-binding transcriptional dual regulator	1.013	1.011	0.84	0.909

Helicases

DnaB	YP_492195.1	replicative DNA helicase	1.056	0.879	1.086	1.049
UvrD	YP_491628.1	DNA-dependent ATPase I and helicase II	0.887	0.874	0.996	0.977
RuvB	YP_490122.1	ATP-dependent DNA helicase, component of RuvABC resolvasome	0.828 *	0.941	0.675	0.679 *
HelD	YP_489234.1	DNA helicase IV	0.761 *	0.815 *	1.022	1.108
RuvA	YP_490123.1	component of RuvABC resolvasome, regulatory subunit	0.881	0.728	1.107	0.797
Rep	YP_491661.1	DNA helicase and single-stranded DNA-dependent ATPase	1.1	1.049	1.2 *	0.953
DinG	YP_489072.1	ATP-dependent DNA helicase	1.173	0.776	1.687	1.105 *
RecG	YP_491782.1	ATP-dependent DNA helicase	---	---	---	---

Primases						
DnaG	YP_491258.1	DNA primase	1.499	0.974	1.299 *	0.836
SSB						
Ssb	YP_492202.1	Single-stranded DNA-binding protein	1.169 *	1.032	1.181 *	1.156 *
RNase H/Pol I		Removal of RNA primer and Gap-filling				
Rne	YP_489352.1	fused ribonucleaseE endoribonuclease and scaffold for formation of degradosome	0.973	1.003	0.961	1.007
VacB	YP_492321.1	exoribonuclease R, RNase R	0.894	0.998	1.05	1.124
Rnb	YP_489554.1	ribonuclease II	1.009	0.897 *	1.049	0.984
CafA	YP_491430.1	ribonuclease G	1.032	0.888 *	1.111	0.898
YoaB	YP_490070.1	hypothetical protein Y75_p1784	1.07	1.026	1.157	1.141
ZyhaR	YP_491302.1	L-PSP (mRNA) endoribonuclease	1.003	0.79 *	1.281 *	1.007
MenG	YP_491522.1	ribonuclease E (RNase E) inhibitor protein	1.215 *	1.067	1.3 *	1.231 *
XthA	YP_490010.1	exonuclease III	1.152	0.99	1.049	0.74 *

XseA	YP_490737.1	exonuclease VII, large subunit	1.056	0.715 *	1.089	0.916
YjgD	YP_492393.1	hypothetical protein Y75_p4138	0.873	0.839	1.184 *	0.925
Orn	YP_492307.1	oligoribonuclease	0.97	1.16	1.055	1.111
Rnd	YP_490065.1	ribonuclease D	0.801	0.775 *	1.024	0.991
Rnc	YP_490795.1	RNase III	0.995	0.875 *	1.094	0.962
YcfH	YP_489368.1	metallodependent hydrolase	1.166	0.796	1.211	0.818
SbcB	YP_490253.1	exonuclease I	0.731	0.823	0.995	1.109
RecD	YP_491024.1	exonuclease V (RecBCD complex) subunit alpha	1.513	1.089	0.888	0.632
RnpA	YP_491731.1	protein C5 component of RNase P	---	---	---	---
Rnt	YP_489916.1	ribonuclease T (RNase T)	1.149	1.079	1.014	0.942
ExoX	YP_490106.1	DNA exonuclease X	1.081	1.097	0.929	0.932
RecC	YP_491027.1	exonuclease V (RecBCD complex) subunit gamma	0.954	0.768	1.089 *	0.869
RnhA	YP_488511.1	ribonuclease HI, degrades RNA of DNA-RNA hybrids	---	---	---	---
DNA ligase		Jioning of Okazaki fragment				
Lig	YP_490647.1	DNA ligase, NAD(+)-dependent	1.072	0.77 *	1.442 *	0.97

DNA gyrase

GyrA	YP_490470.1	DNA gyrase (type II topoisomerase), subunit A	0.92	1.001	0.938	0.977
GyrB	YP_491736.1	DNA gyrase, subunit B	0.988	1.217	0.799	0.999
SbmC	YP_490251.1	DNA gyrase inhibitor	1.065	1.087	1.255 *	1.19
ParC	YP_491211.1	DNA topoisomerase IV, subunit A	0.917	0.966	0.836 *	0.887 *
ParE	YP_491222.1	DNA topoisomerase IV, subunit B	1.025	0.949	1.032	0.943
BarA_1	YP_490994.1	hybrid sensory histidine kinase, in two-component regulatory system with UvrY	1.383	1.433 *	0.977	1.002
DnaA	YP_491733.1	chromosomal replication initiator protein DnaA, DNA-binding transcriptional dual regulator	1.013	1.011	0.84	0.909
DnaC	YP_492492.1	DNA biosynthesis protein	0.828	0.951	0.916	1.041

DNAI

PriA	YP_491516.1	primosome factor n~	0.71	0.813	0.942	1.066

DNA topoisomerase

GyrA	YP_490470.1	DNA gyrase (type II topoisomerase), subunit A	0.92	1.001	0.938	0.977
GyrB	YP_491736.1	DNA gyrase, subunit B	0.988	1.217	0.799	0.999
TopA_	YP_489542.1	DNA topoisomerase I, omega subunit	0.77	1.134	0.843 *	1.216
ParC	YP_491211.1	DNA topoisomerase IV, subunit A	0.917	0.966	0.836 *	0.887 *
ParE	YP_491222.1	DNA topoisomerase IV, subunit B	1.025	0.949	1.032	0.943
TopB	YP_490024.1	DNA topoisomerase III	---	---	---	---

DNA repair

Base excision repair

Lig	YP_490647.1	DNA ligase, NAD(+)-dependent	1.072	0.77 *	1.442 *	0.97
XthA	YP_490010.1	exonuclease III	1.152	0.99	1.049	0.74 *
XseA	YP_490737.1	exonuclease VII, large subunit	1.056	0.715 *	1.089	0.916
RecD	YP_491024.1	exonuclease V (RecBCD complex) subunit alpha	1.513	1.089	0.888	0.632
ExoX	YP_490106.1	DNA exonuclease X	1.081	1.097	0.929	0.932
RecC	YP_491027.1	exonuclease V (RecBCD complex) subunit gamma	0.954	0.768	1.089 *	0.869

Nucleotide excision repair

Mfd	YP_489382.1	transcription-repair coupling factor	1.054	0.889 *	1.013	0.942
UvrD	YP_491628.1	DNA-dependent ATPase I and helicase II	0.887	0.874	0.996	0.977
HelD	YP_489234.1	DNA helicase IV	0.761 *	0.815 *	1.022	1.108
Rep	YP_491661.1	DNA helicase and single-stranded DNA-dependent ATPase	1.1	1.049	1.2 *	0.953
UvrA	YP_492201.1	ATPase and DNA damage recognition protein of nucleotide excision repair excinuclease UvrABC	0.823 *	0.831 *	1.009	1.056
UvrB	YP_489052.1	excinulease of nucleotide excision repair, DNA damage recognition component	1.089	0.88	0.981	0.819 *
UvrC	YP_490170.1	excinuclease UvrABC, endonuclease subunit	1.202	1.16	0.835	0.785
UvrD	YP_491628.1	DNA-dependent ATPase I and helicase II	0.887	0.874	0.996	0.977
UvrY	YP_490171.1	DNA-binding response regulator in two-component regulatory system with BarA	0.968	0.822	1.027	0.737 *
BarA_[illegible]	YP_490994.1	hybrid sensory histidine kinase, in two-component regulatory system with UvrY	1.383	1.433 *	0.977	1.002
UvrA	YP_492201.1	ATPase and DNA damage recognition protein of nucleotide excision repair excinuclease UvrABC	0.823 *	0.831 *	1.009	1.056
UvrC	YP_490170.1	excinuclease UvrABC, endonuclease subunit	1.202	1.16	0.835	0.785

Mismatch repair

UvrD	YP_491628.1	DNA-dependent ATPase I and helicase II	0.887	0.874	0.996	0.977
HelD	YP_489234.1	DNA helicase IV	0.761 *	0.815 *	1.022	1.108
Lig	YP_490647.1	DNA ligase, NAD(+)-dependent	1.072	0.77 *	1.442 *	0.97
MutS	YP_490942.1	methyl-directed mismatch repair protein	0.908	0.865	1.125	1.042
MutL	YP_492312.1	methyl-directed mismatch repair protein	1.202	0.681 *	1.588	1.083
XthA	YP_490010.1	exonuclease III	1.152	0.99	1.049	0.74 *
XseA	YP_490737.1	exonuclease VII, large subunit	1.056	0.715 *	1.089	0.916
SbcB	YP_490253.1	exonuclease I	0.731	0.823	0.995	1.109
RecD	YP_491024.1	exonuclease V (RecBCD complex) subunit alpha	1.513	1.089	0.888	0.632
ExoX	YP_490106.1	DNA exonuclease X	1.081	1.097	0.929	0.932
RecC	YP_491027.1	exonuclease V (RecBCD complex) subunit gamma	0.954	0.768	1.089 *	0.869
RecJ	YP_491093.1	ssDNA exonuclease, 5~ --> 3~-specific	0.824	0.847	1.107	1.227
Ssb	YP_492202.1	Single-stranded DNA-binding protein	1.169 *	1.032	1.181 *	1.156 *

Homologous recombination

RecA	YP_490908.1	DNA strand exchange and recombination protein with protease and nuclease activity	1.032	1.035	0.998	0.956
HepA	YP_488365.1	RNA polymerase-associated helicase protein	0.975	1.013	1.046	0.955
YigN	YP_491610.1	recombination limiting protein	1.489	0.58 *	2.072	0.726
RecJ	YP_491093.1	ssDNA exonuclease, 5~ --> 3~-specific	0.824	0.847	1.107	1.227
RecD	YP_491024.1	exonuclease V (RecBCD complex) subunit alpha	1.513	1.089	0.888	0.632
RecC	YP_491027.1	exonuclease V (RecBCD complex) subunit gamma	0.954	0.768	1.089 *	0.869
RecG	YP_491782.1	ATP-dependent DNA helicase	---	---	---	---
RuvB	YP_490122.1	ATP-dependent DNA helicase, component of RuvABC resolvasome	0.828 *	0.941	0.675	0.679 *
RuvA	YP_490123.1	component of RuvABC resolvasome, regulatory subunit	0.881	0.728	1.107	0.797
PolA_2	YP_491586.1	fused DNA polymerase I 5~->3~ exonuclease, 3~->5~ polymerase and 3~->5~ exonuclease	0.902	0.993	0.927	1.066
DnaN	YP_491734.1	DNA polymerase III subunit beta	1.013	0.821 *	1.262 *	1.083
DnaX	YP_488761.1	DNA polymerase III/DNA elongation factor III, tau and gamma subunits	1.005	0.884	1.183	1.127
DnaE	YP_488486.1	DNA polymerase III alpha subunit	1.107	0.819	1.215	0.823
HolC	YP_492397.1	DNA polymerase III, chi subunit	---	---	---	---

HolA	YP_488931.1	DNA polymerase III subunit delta	0.808	0.58	1.748	1.241
PolB	YP_488366.1	DNA polymerase II	---	---	---	---

Recombination-associated proteins

DNA integration/recombination/inversion

HsdR	YP_492481.1	endonuclease R	0.901 *	0.93	0.888 *	0.948
Dcm	YP_490214.1	DNA cytosine methylase	1.018	0.909	0.847	0.851 *
YbcK	YP_488831.1	recombinase	---	---	---	---
RdgC	YP_488686.1	DNA-binding protein, non-specific	0.874 *	1.122	0.883 *	1.087 *
YigN	YP_491610.1	recombination limiting protein	1.489	0.58 *	2.072	0.726

Transposase

YafM	YP_488525.1	hypothetical protein Y75_p0219	---	---	---	---
InsL	YP_490635.1	transposase > YP_488870.1 IS186/IS421 transposase > YP_488322.1 IS186/IS421 transposase [Escherichia coli str. K-12 [illegible]	---	---	---	---
YhgA	YP_492021.1	transposase	---	---	---	---

Other

HepA	YP_488365.1	RNA polymerase-associated helicase protein	0.975	1.013	1.046	0.955
Sms	YP_492519.1	repair protein	0.838	1.001	0.864	1.125
YajR	YP_488719.1	transporter	---	---	---	---
GyrA	YP_490470.1	DNA gyrase (type II topoisomerase), subunit A	0.92	1.001	0.938	0.977
GyrB	YP_491736.1	DNA gyrase, subunit B	0.988	1.217	0.799	0.999
TopA_	YP_489542.1	DNA topoisomerase I, omega subunit	0.77	1.134	0.843 *	1.216
ParC	YP_491211.1	DNA topoisomerase IV, subunit A	0.917	0.966	0.836 *	0.887 *
ParE	YP_491222.1	DNA topoisomerase IV, subunit B	1.025	0.949	1.032	0.943
TopB	YP_490024.1	DNA topoisomerase III	---	---	---	---
HepA	YP_488365.1	RNA polymerase-associated helicase protein	0.975	1.013	1.046	0.955
YcbY_	YP_489220.1	methyltransferase	1.135	0.96	0.958	0.968
Dcm	YP_490214.1	DNA cytosine methylase	1.018	0.909	0.847	0.851 *
Dps	YP_489085.1	Fe-binding and storage protein	0.381	0.379 *	1.185 *	1.13

Ogt	YP_489605.1	O-6-alkylguanine-DNA:cysteine-protein methyltransferase	---	---	---	---
HupA	YP_491460.1	HU, DNA-binding transcriptional regulator subunit alpha	1.577 *	1.445 *	0.776	0.653 *
HupB	YP_488732.1	HU, DNA-binding transcriptional regulator subunit beta	1.502 *	1.054	0.953	0.67 *
Ogt	YP_489605.1	O-6-alkylguanine-DNA:cysteine-protein methyltransferase	---	---	---	---
DksA	YP_488448.1	transcriptional regulator of rRNA transcription, DnaK suppressor protein	1.092	0.97	0.96	0.913
DnaK	YP_488320.1	chaperone Hsp70, co-chaperone with DnaJ	1.08	1.023	1.064	0.994
DnaJ	YP_488321.1	chaperone Hsp40, co-chaperone with DnaK	0.767 *	1.022	0.756 *	0.989
DksA	YP_488448.1	transcriptional regulator of rRNA transcription, DnaK suppressor protein	1.092	0.97	0.96	0.913
HepA	YP_488365.1	RNA polymerase-associated helicase protein	0.975	1.013	1.046	0.955
HrpA	YP_489680.1	ATP-dependent helicase	0.948	0.854 *	1.111 *	0.946
DinG	YP_489072.1	ATP-dependent DNA helicase	1.173	0.776	1.687	1.105 *
Lhr	YP_489917.1	ATP-dependent helicase	1.165	0.835	1.173	0.832

Transcription

RNA polymerase

RpoB	YP_491474.1	RNA polymerase subunit beta	0.966	1.123	0.896	1.06
RpoC	YP_491473.1	RNA polymerase, beta prime subunit	0.946	1.035	0.969	1.06
RpoA	YP_492137.1	RNA polymerase subunit alpha	0.981	1.062 *	0.89	1.054
RpoD	YP_491259.1	RNA polymerase, sigma 70 (sigma D) factor	0.974	1.041	1.042	1.016
RpoZ	YP_491785.1	RNA polymerase, omega subunit	1.337	0.933	1.045	0.783 *
RpoN	YP_491387.1	RNA polymerase, sigma 54 (sigma N) factor	0.997	0.84	0.928	1.189
RpoE	YP_490801.1	RNA polymerase, sigma 24 (sigma E) factor	0.745	0.964	1.049	1.265

Sigma factors

RpoD	YP_491259.1	RNA polymerase, sigma 70 (sigma D) factor	0.974	1.041	1.042	1.016
YhbL	YP_491394.1	isoprenoid biosynthesis protein with amidotransferase-like domain	1.009	0.84 *	1.119	1.003
YhbH	YP_491388.1	ribosome-associated, sigma 54 modulation protein	0.943	0.842 *	0.921	0.881
RseB	YP_490799.1	anti-sigma factor	0.999	0.892	1.078	1.035
Crl	YP_488535.1	DNA-binding transcriptional regulator	1.012	1.162	0.761	0.867 *
PspA	YP_489572.1	regulatory protein for phage-shock-protein operon	1.286 *	2.761 *	0.964	2.095 *

RpoN	YP_491387.1	RNA polymerase, sigma 54 (sigma N) factor	0.997	0.84	0.928	1.189
YjhU	YP_492427.1	DNA-binding transcriptional regulator	0.954	0.955	0.973	1.012
RpoE	YP_490801.1	RNA polymerase, sigma 24 (sigma E) factor	0.745	0.964	1.049	1.265
YaeL	YP_488478.1	zinc metallopeptidase	1.147	1.023	1.002	0.872
YgeV	YP_491071.1	DNA-binding transcriptional regulator	1.042	1.053	0.967	0.964
YdeW	YP_489775.1	DNA-binding transcriptional regulator	1.251	1.09	0.857	0.703 *
YfiA	YP_490820.1	cold shock protein associated with 30S ribosomal subunit	1.208	1.025	0.636	0.742

Transcription-associated proteins

Mfd	YP_489382.1	transcription-repair coupling factor	1.054	0.889 *	1.013	0.942
NusA	YP_491356.1	transcription termination/antitermination L factor	1.11 *	1.043	1.081	1.072
NusG	YP_491479.1	transcription termination factor	1.081	0.98	0.87	0.82
NusB	YP_488708.1	transcription antitermination protein	1.05	1.126 *	0.953	0.949
GreA	YP_491366.1	transcription elongation factor	1.085 *	1.022	1.309 *	1.236
ZgreB	YP_492026.1	transcription elongation factor	0.874	0.667	1.18	0.891

Rho	YP_491656.1	transcription termination factor	0.99	1.181	0.869	1.03

RNA degradation

Type A *E. coli*		RNase E+RhlB+Enolase+PNPase				
Rne	YP_489352.1	fused ribonucleaseE endoribonuclease and scaffold for formation of degradosome	0.973	1.003	0.961	1.007
MenG	YP_491522.1	ribonuclease E (RNase E) inhibitor protein	1.215 *	1.067	1.3 *	1.231 *
Rnc	YP_490795.1	RNase III	0.995	0.875 *	1.094	0.962
RnpA	YP_491731.1	protein C5 component of RNase P	---	---	---	---
Rnt	YP_489916.1	ribonuclease T (RNase T)	1.149	1.079	1.014	0.942
Pnp	YP_491351.1	polynucleotide phosphorylase/polyadenylase	1.085 *	1.067	1.035	1.01
YaiL	YP_488648.1	nucleoprotein/polynucleotide-associated enzyme	1.324	0.783	1.274	0.745 *
YhdA	YP_491435.1	inner membrane protein	---	---	---	---
Type B *Psudomonas*		RNase E+RhlE+RNase R				
VacB	YP_492321.1	exoribonuclease R, RNase R	0.894	0.998	1.05	1.124

Rnb	YP_489554.1	ribonuclease II	1.009	0.897 *	1.049	0.984
Type C *Rhodobacter*		RNase E+Helicases+Rho				
HepA	YP_488365.1	RNA polymerase-associated helicase protein	0.975	1.013	1.046	0.955
Rho	YP_491656.1	transcription termination factor	0.99	1.181	0.869	1.03
Type D *Bacillus subtil*		PNPase+RNase R+RNase J				
gloB	YP_488509.1	hydroxyacylglutathione hydrolase	0.94	0.918	1.094	1.019
associated proteins						
GroL	YP_492286.1	Cpn60 chaperonin GroEL, large subunit of GroESL	1.113	1.129	1.127	1.141
GroS	YP_492285.1	Cpn10 chaperonin GroES, small subunit of GroESL	1.146 *	1.165 *	1.027	1.088
Hfq	YP_492314.1	HF-I, host factor for RNA phage Q beta replication	0.803 *	1.076	0.744 *	0.984
DksA	YP_488448.1	transcriptional regulator of rRNA transcription, DnaK suppressor protein	1.092	0.97	0.96	0.913
Ppk	YP_490729.1	polyphosphate kinase, component of RNA degradosome	0.94	0.933	1.068	1.124 *

DnaK	YP_488320.1	chaperone Hsp70, co-chaperone with DnaJ	1.08	1.023	1.064	0.994
DksA	YP_488448.1	transcriptional regulator of rRNA transcription, DnaK suppressor protein	1.092	0.97	0.96	0.913
DnaJ	YP_488321.1	chaperone Hsp40, co-chaperone with DnaK	0.767 *	1.022	0.756 *	0.989

Nucleases

Rne	YP_489352.1	fused ribonucleaseE endoribonuclease and scaffold for formation of degradosome	0.973	1.003	0.961	1.007
VacB	YP_492321.1	exoribonuclease R, RNase R	0.894	0.998	1.05	1.124
Rnb	YP_489554.1	ribonuclease II	1.009	0.897 *	1.049	0.984
CafA	YP_491430.1	ribonuclease G	1.032	0.888 *	1.111	0.898
YoaB	YP_490070.1	hypothetical protein Y75_p1784	1.07	1.026	1.157	1.141
MenG	YP_491522.1	ribonuclease E (RNase E) inhibitor protein	1.215 *	1.067	1.3 *	1.231 *
XthA	YP_490010.1	exonuclease III	1.152	0.99	1.049	0.74 *
XseA	YP_490737.1	exonuclease VII, large subunit	1.056	0.715 *	1.089	0.916
YjgD	YP_492393.1	hypothetical protein Y75_p4138	0.873	0.839	1.184 *	0.925
Orn	YP_492307.1	oligoribonuclease	0.97	1.16	1.055	1.111

Rnd	YP_490065.1	ribonuclease D	0.801	0.775 *	1.024	0.991
Rnc	YP_490795.1	RNase III	0.995	0.875 *	1.094	0.962
YcfH	YP_489368.1	metallodependent hydrolase	1.166	0.796	1.211	0.818
SbcB	YP_490253.1	exonuclease I	0.731	0.823	0.995	1.109
RecD	YP_491024.1	exonuclease V (RecBCD complex) subunit alpha	1.513	1.089	0.888	0.632
RnpA	YP_491731.1	protein C5 component of RNase P	---	---	---	---
Rnt	YP_489916.1	ribonuclease T (RNase T)	1.149	1.079	1.014	0.942
ExoX	YP_490106.1	DNA exonuclease X	1.081	1.097	0.929	0.932
RecC	YP_491027.1	exonuclease V (RecBCD complex) subunit gamma	0.954	0.768	1.089 *	0.869
RnhA	YP_488511.1	ribonuclease HI, degrades RNA of DNA-RNA hybrids	---	---	---	---
Add	YP_489886.1	adenosine deaminase	1.077	0.86 *	1.158 *	0.937
PolA_2	YP_491586.1	fused DNA polymerase I 5~->3~ exonuclease, 3~->5~ polymerase and 3~->5~ exonuclease	0.902	0.993	0.927	1.066
VacB	YP_492321.1	exoribonuclease R, RNase R	0.894	0.998	1.05	1.124
Rnb	YP_489554.1	ribonuclease II	1.009	0.897 *	1.049	0.984
XthA	YP_490010.1	exonuclease III	1.152	0.99	1.049	0.74 *

XseA	YP_490737.1	exonuclease VII, large subunit	1.056	0.715 *	1.089	0.916
Nfo	YP_490398.1	endonuclease IV with intrinsic 3~-5~ exonuclease activity	0.876	1.035	0.738 *	0.951
Orn	YP_492307.1	oligoribonuclease	0.97	1.16	1.055	1.111
Rnd	YP_490065.1	ribonuclease D	0.801	0.775 *	1.024	0.991
RecJ	YP_491093.1	ssDNA exonuclease, 5~ --> 3~-specific	0.824	0.847	1.107	1.227
Exo	YP_491006.1	exonuclease IX (5~-3~ exonuclease)	0.939	1.094	1.171	1.351
SbcB	YP_490253.1	exonuclease I	0.731	0.823	0.995	1.109
RecD	YP_491024.1	exonuclease V (RecBCD complex) subunit alpha	1.513	1.089	0.888	0.632
Rnt	YP_489916.1	ribonuclease T (RNase T)	1.149	1.079	1.014	0.942
ExoX	YP_490106.1	DNA exonuclease X	1.081	1.097	0.929	0.932
SbcC	YP_488689.1	exonuclease, dsDNA, ATP-dependent	---	---	---	---
RecC	YP_491027.1	exonuclease V (RecBCD complex) subunit gamma	0.954	0.768	1.089 *	0.869
XthA	YP_490010.1	exonuclease III	1.152	0.99	1.049	0.74 *
XseA	YP_490737.1	exonuclease VII, large subunit	1.056	0.715 *	1.089	0.916
Orn	YP_492307.1	oligoribonuclease	0.97	1.16	1.055	1.111

RecJ	YP_491093.1	ssDNA exonuclease, 5~ --> 3~-specific	0.824		0.847		1.107		1.227	
Exo	YP_491006.1	exonuclease IX (5~-3~ exonuclease)	0.939		1.094		1.171		1.351	
SbcB	YP_490253.1	exonuclease I	0.731		0.823		0.995		1.109	
ExoX	YP_490106.1	DNA exonuclease X	1.081		1.097		0.929		0.932	
SbcC	YP_488689.1	exonuclease, dsDNA, ATP-dependent	---		---		---		---	
RecC	YP_491027.1	exonuclease V (RecBCD complex) subunit gamma	0.954		0.768		1.089	*	0.869	
XthA	YP_490010.1	exonuclease III	1.152		0.99		1.049		0.74	*
Rnc	YP_490795.1	RNase III	0.995		0.875	*	1.094		0.962	
RnpA	YP_491731.1	protein C5 component of RNase P	---		---		---		---	

Note: [a] 1-vs-2, 12 h with 0 mM versus 1 mM Mn (II); 3-vs-4, 48 h with 0 mM versus 1 mM Mn (II); 1-vs-3, 12 h versus 48 h with 0 mM Mn (II); and 2-vs-4, 12 h versus 48 h with 1 mM Mn (II). Red value denotes up-regulated expression (>1.2 fold); Green value denotes down-regulated (< 0.833 fold).

[b] Sig, significance.

[c] *, *p* -values < 0.05 and the up-regulated value >1.2 fold / down-regulated value < 0.833 fold.

Table S4. Up- or down-regulated protein synthesis related proteins. [a–c]

Protein_or_Domain	Accession num	COG Function-Desc	1-vs-2	Sig	3-vs-4	Sig	1-vs-3	Sig	2-vs-4	Sig
Aminoacyl-tRNA biosynthesis										
L-Alanine+tRNA-Ala→L-Alanyl-tRNA(Ala)										
AlaS	YP_490906.1	Alanyl-tRNA syntheta	1.001		1.175		0.866		0.974	
L-Arginine+tRNA-Ala→L-Arginyl-tRNA(Ala)										
ArgS	YP_490138.1	Arginyl-tRNA synthe	0.875		1.025		0.938		1.041	
L-Aspartate+tRNA-Asp→L-Aspartyl-tRNA(Asp)										
AspS	YP_490128.1	Aspartyl-tRNA synth	1.023		1.041		1.033		1.069	
L-Aspartate+tRNA-Asn→L-Aspartyl-tRNA(Asn)										
AspC	YP_489200.1	Aspartate/tyrosine/aro	0.895	*	0.964		0.888	*	0.996	
L-Asparagine+tRNA-Asn→L-Asparaginyl-tRNA(Asn)										
AsnS	YP_489202.1	Aspartyl/asparaginyl-t	0.957		0.994		0.952		1.039	
L-Aspartyl-tRNA(Asn)→L-Asparaginyl-tRNA(Asn)										
GatZ	YP_490335.1	Predicted tagatose 6-p	1.005		0.951		1.021		1.02	

	GatB	YP_490331.1	Phosphotransferase sy	1.293	*	0.792 *	0.607		0.381	*
	GatY	YP_490336.1	Fructose/tagatose bisp	1.05		1.012	1.118	*	1.053	
	GatD	YP_490329.1	Threonine dehydroger	1.029		1.101	0.773	*	0.89	
	GatC	YP_490330.1	Phosphotransferase sy	1.071		0.815 *	0.692	*	0.539	*
L-Cysteine+tRNA-Cys→L-Cysteinyl-tRNA(Cys)										
	CysS	YP_488815.1	Cysteinyl-tRNA synth	0.885	*	0.884 *	1.042		0.984	
L-Glutamate+tRNA-Glu/Gln→L-Glutamyl-tRNA(Glu/Gln)										
	GltX	YP_490639.1	Glutamyl- and glutam	0.835	*	0.884	0.91		0.946	
L-Glutamyl-tRNA(Gln)→L-Glutaminyl-tRNA(Gln)										
	GatZ	YP_490335.1	Predicted tagatose 6-p	1.005		0.951	1.021		1.02	
	GatB	YP_490331.1	Phosphotransferase sy	1.293	*	0.792 *	0.607		0.381	*
	GatY	YP_490336.1	Fructose/tagatose bisp	1.05		1.012	1.118	*	1.053	
	GatD	YP_490329.1	Threonine dehydroger	1.029		1.101	0.773	*	0.89	
	GatC	YP_490330.1	Phosphotransferase sy	1.071		0.815 *	0.692	*	0.539	*
	PepN	YP_489204.1	Aminopeptidase N	1.011		1.072	0.923		0.99	
	GltX	YP_490639.1	Glutamyl- and glutam	0.835	*	0.884	0.91		0.946	
	GlnS	YP_488960.1	Glutamyl- and glutam	0.837	*	1.096	0.871		1.013	

Glycine+tRNA-Gly→Glycyl-tRNA(Gly)										
GlyS	YP_491876.1	Glycyl-tRNA syntheta	1.003		1.048		0.993		0.985	
L-Histidine+tRNA-His→L-Histidyl-tRNA(His)										
HisS	YP_490742.1	Histidyl-tRNA synthe	0.94		1.074		0.956		1.046	
L-Isoleucine+tRNA-Ile→L-Isoleucyl-tRNA(Ile)										
IleS	YP_488332.1	Isoleucyl-tRNA synth	0.912		1.028		0.963		1.115	
L-Leucine+tRNA-Leu→L-Leucyl-tRNA(Leu)										
LeuS	YP_488933.1	Leucyl-tRNA syntheta	0.938		1.088	*	0.912	*	1.058	
L-Lysine+tRNA-Lys→L-Lysyl-tRNA(Lys)										
LysS	YP_491091.1	Lysyl-tRNA synthetas	1.022		1.157	*	0.824		0.97	
L-Methionine+tRNA-Met→L-Methionyl-tRNA(Met)										
MetG_1	YP_490354.1	Methionyl-tRNA synt	0.975		1.133		0.986		1.088	*
L-Methionyl-tRNA(Met)+10-Formyl-THF→N-Formyl-L-Methionyl-tRNA(fMet)										
Fmt	YP_492145.1	Methionyl-tRNA forn	1.187	*	0.92		1.286	*	0.884	
L-Phenylalanine+tRNA-Phe→L-Phenylalanyl-tRNA(Phe)										
PheS	YP_489976.1	Phenylalanyl-tRNA sy	0.767	*	0.87	*	0.893	*	1.016	
ECs2420_	YP_489975.1	Phenylalanyl-tRNA sy	0.839		1.004		0.867	*	1.029	

L-Proline+tRNA-Pro→L-Prolyl-tRNA(Pro)							
	ProS	YP_488497.1	Prolyl-tRNA syntheta	1.079 *	1.006	1.135	0.976
L-Serine+tRNA-Ser→L-Seryl-tRNA(Ser)							
	SerS	YP_489165.1	Seryl-tRNA synthetas	0.824 *	0.931	0.845 *	1.029
L-Threonine+tRNA-Thr→L-Threonyl-tRNA(Thr)							
	ThrS	YP_489981.1	Threonyl-tRNA synth	0.802	0.931	0.844	1.049
L-Tryptophan+tRNA-Trp→L-Tryptophanyl-tRNA(Trp)							
	TrpS	YP_492048.1	Tryptophanyl-tRNA s	1.208 *	1.396 *	0.777	0.984
L-Tyrosine+tRNA-Tyr→L-Tyrosyl-tRNA(Tyr)							
	ZtyrS	YP_489901.1	Tyrosyl-tRNA synthe	1.068	0.997	1.093 *	0.988
L-Valine+tRNA-Val→L-Valyl-tRNA(Val)							
	ValS	YP_492396.1	Valyl-tRNA synthetas	1.045	0.995	0.938	0.893
Ribosomal structural proteins							
	RpsA	YP_489183.1	Ribosomal protein S1	1.024	1.055	1.002	1.018
	RplA	YP_491477.1	Ribosomal protein L1	1.035	1.099	0.833	0.815
	RplL	YP_491475.1	Ribosomal protein L7	1.097	1.118	0.959	0.974

RplE	YP_492124.1	Ribosomal protein L5	1.025		0.934		0.864	*	0.835	
RpsB	YP_488471.1	Ribosomal protein S2	0.965		1.045		0.881		0.919	
RplI	YP_492345.1	Ribosomal protein L9	0.996		1.062		0.933		0.995	
RpsC	YP_492118.1	Ribosomal protein S3	0.944		1.117	*	0.789		0.963	
RpsE	YP_492129.1	Ribosomal protein S5	0.94	*	1.251	*	0.654	*	0.807	*
RplO	YP_492131.1	Ribosomal protein L1	1.1		0.982		1.198	*	0.983	
RpsM	YP_492134.1	Ribosomal protein S1	0.953		1.156		0.8		0.981	
RpsD	YP_492136.1	Ribosomal protein S4	0.875		1.197	*	0.829	*	1.085	
RplB	YP_492115.1	Ribosomal protein L2	0.972		1.189		1.003		1.195	
RplF	YP_492127.1	Ribosomal protein L6	1.199	*	1.299		0.931	*	0.995	
RpsG	YP_492091.1	Ribosomal protein S7	0.927		1.125	*	0.748	*	0.865	
RplJ	YP_491476.1	Ribosomal protein L1	1.017		0.94		0.996		0.988	
RplK	YP_491478.1	Ribosomal protein L1	1.24	*	0.952		1.211	*	0.91	
RplD	YP_492113.1	Ribosomal protein L4	0.825	*	0.975		0.973		1.173	
RplP	YP_492119.1	Ribosomal protein L1	0.977		1.087		0.929	*	1.039	
RpsH	YP_492126.1	Ribosomal protein S8	1.136	*	0.922	*	1.184		0.967	
RplV	YP_492117.1	Ribosomal protein L2	1.214	*	1.406		0.937		1.083	

RpsJ	YP_492111.1	Ribosomal protein S1	0.929	*	1.14		0.722		0.9	
RplN	YP_492122.1	Ribosomal protein L1	0.927		1.008		0.92		1.018	
RpsI	YP_491414.1	Ribosomal protein S9	0.803		1.12	*	0.811		1.053	
RplX	YP_492123.1	Ribosomal protein L2	1.302		1.472	*	0.765	*	0.931	
RpsF	YP_492342.1	Ribosomal protein S6	0.834	*	0.97		0.753	*	0.862	
RplQ	YP_492138.1	Ribosomal protein L1	1.067		1.139	*	1.051		1.063	
RplM	YP_491415.1	Ribosomal protein L1	0.966		1.015		0.91		1.016	
RplC	YP_492112.1	Ribosomal protein L3	1.403	*	1.334	*	1.036		0.95	
RplU	YP_491371.1	Ribosomal protein L2	1.054		1.218	*	0.873	*	0.95	
RplS	YP_490829.1	Ribosomal protein L1	1.062	*	1.091	*	0.831	*	0.857	*
RpmA	YP_491370.1	Ribosomal protein L2	1.131		1.032		1.061		0.979	
RpsS	YP_492116.1	Ribosomal protein S1	1.045		1.437	*	0.645		0.969	
RpsK	YP_492135.1	Ribosomal protein S1	0.797	*	0.991		0.918		1.089	
RplY	YP_490425.1	Ribosomal protein L2	0.93		1.101		0.757		0.886	
RplT	YP_489978.1	Ribosomal protein L2	0.998		1.191	*	0.794	*	0.912	*
RpsR	YP_492344.1	Ribosomal protein S1	1.387	*	1.447	*	0.93		1.075	
RpmD	YP_492130.1	Ribosomal protein L3	0.971		1.283	*	0.862	*	0.996	

RpsT	YP_488329.1	Ribosomal protein S2	1.601		1.587		1.145		1.102	
RpsP	YP_490832.1	Ribosomal protein S1	1.007		1.026		0.613		0.679	
RpsO	YP_491352.1	Ribosomal protein S1	0.986		0.881	*	1.087		1.09	
RpmC	YP_492120.1	Ribosomal protein L2	1.155	*	1.217		0.621	*	0.741	
RpsQ	YP_492121.1	Ribosomal protein S1	0.782		1.067		0.909		1.11	
YfcB	YP_490572.1	Methylase of polypep	0.995		1.483		0.659		0.922	
RpsU	YP_491257.1	Ribosomal protein S2	2.398	*	2.674	*	1.299		1.286	*
RpsN	YP_492125.1	Ribosomal protein S1	2.161	*	2.247		1.017		0.994	
PrmA	YP_491441.1	Ribosomal protein L1	1.081		0.605		1.515	*	1.032	
RpsL	YP_492090.1	Ribosomal protein S1	1.491	*	1.171		1.138		1.038	
RpmF	YP_489357.1	Ribosomal protein L3	0.733	*	1.46	*	0.846	*	1.762	*
RpmI	YP_489979.1	Ribosomal protein L3	---		---		---		---	
RpmE	YP_491515.1	Ribosomal protein L3	---		---		---		---	
RplW	YP_492114.1	Ribosomal protein L2	0.668		1.366	*	0.834		1.687	
RpmH	YP_491732.1	Ribosomal protein L3	---		---		---		---	
RplR	YP_492128.1	Ribosomal protein L1	---		---		---		---	

Ribosomal biogenesis--associated protein

YciL	YP_489537.1	16S rRNA uridine-51(	0.89	*	0.958		0.905		0.944	
SfhB	YP_490818.1	Pseudouridylate synth	0.936		1.054		0.994		0.993	
YjbC	YP_492165.1	16S rRNA uridine-51(	1.14		0.836		1.42		1.019	
RluC	YP_489354.1	Pseudouridylate synth	0.789		0.887		0.992		1.104	
YmfC	YP_489403.1	16S rRNA uridine-51(	---		---		---		---	
Frr	YP_488474.1	Ribosome recycling fa	1.325	*	0.926		1.047		0.754	*
YhbH	YP_491388.1	Ribosome-associated	0.943		0.842	*	0.921		0.881	
YbeB	YP_488928.1	Uncharacterized homo	1.056		1.048		0.884		0.886	
YfiA	YP_490820.1	Ribosome-associated	1.208		1.025		0.636		0.742	

Ribosomal-protein-alanine/serine acetyltransferase

RimJ	YP_489334.1	Acetyltransferases, in(	1.492		0.708	*	1.796	*	0.865	

rRNA tRNA processing--associated protein

FtsJ	YP_491364.1	23S rRNA methylase	0.826		0.907		1.107		1.362	
YciL	YP_489537.1	16S rRNA uridine-51(	0.89	*	0.958		0.905		0.944	

RimM	YP_490831.1	RimM protein, require	1.251	*	1.067	1.183	1.045	
Sun	YP_492144.1	tRNA and rRNA cyto	1.056		0.905	1.064	1.025	
YjbC	YP_492165.1	16S rRNA uridine-51	1.14		0.836	1.42	1.019	
Era	YP_490794.1	GTPase	0.803	*	0.87	0.835	0.815	
YmfC	YP_489403.1	16S rRNA uridine-51	---		---	---	---	
TruA	YP_490560.1	Pseudouridylate synth	---		---	---	---	
YfiF	YP_490809.1	rRNA methylases	1		1.018	1.057	1.166	*
Sun	YP_492144.1	tRNA and rRNA cyto	1.056		0.905	1.064	1.025	
FtsJ	YP_491364.1	23S rRNA methylase	0.826		0.907	1.107	1.362	
LasT	YP_492533.1	rRNA methylase	1.021		0.892	1.653	1.43	
MiaA	YP_492313.1	tRNA delta(2)-isopent	0.83		0.994	1.174	1.035	
MiaB	YP_488952.1	2-methylthioadenine s	0.929		0.901	0.903	0.834	
TruB	YP_491353.1	Pseudouridine synthas	0.855		1.003	0.904	0.903	
GidA	YP_491688.1	NAD/FAD-utilizing e	0.878		0.893	0.885	0.95	

GidB	YP_491689.1	Predicted S-adenosylr	---		---		---		---
TrmD	YP_490830.1	tRNA-(guanine-N1)-r	1.076		1.102		0.865		0.885
RimM	YP_490831.1	RimM protein, require	1.251	*	1.067		1.183		1.045
TrmU	YP_489401.1	Predicted tRNA(5-me	0.915		1.069		1.17		1.187
Dtd	YP_491563.1	D-Tyr-tRNAtyr deacy	1.499	*	0.981		1.815	*	1.176
Tgt	YP_488698.1	Queuine/archaeosine t	1.237	*	1.185		0.954		1.04
QueA	YP_488697.1	S-adenosylmethionine	1.07		0.853		1.046		0.967
FtsJ	YP_491364.1	23S rRNA methylase	0.826		0.907		1.107		1.362
YggH	YP_491159.1	Predicted S-adenosylr	1.071		1.527	*	0.625	*	0.959
GidA	YP_491688.1	NAD/FAD-utilizing e	0.878		0.893		0.885		0.95
GidB	YP_491689.1	Predicted S-adenosylr	---		---		---		---
ThdF	YP_491728.1	Predicted GTPase	0.862	*	0.737	*	0.88		0.768

Translation-associated proteins

YjgF	YP_492383.1	Putative translation in	1.105	*	0.821	*	1.291	*	0.935
YoaB	YP_490070.1	Putative translation in	1.07		1.026		1.157		1.141

YhaR	YP_491302.1	Putative translation in	1.003		0.79	*	1.281	*	1.007	
Pth	YP_489471.1	Peptidyl-tRNA hydrol	1.023		0.995		0.921		0.83	
FusA	YP_492092.1	Translation elongatior	1.024		1.086		0.986		1.093	
YihK	YP_491579.1	Predicted membrane (	1.035		1.085	*	1		1.032	
LepA	YP_490797.1	Membrane GTPase Lε	0.96		0.998		1.001		1.037	
GreA	YP_491366.1	Transcription elongati	1.085	*	1.022		1.309	*	1.236	
PrfC	YP_492504.1	Peptide chain release :	0.983		1.116		0.827	*	0.957	
GreB	YP_492026.1	Transcription elongati	0.874		0.667		1.18		0.891	
TufB	YP_491481.1	GTPases - translation	0.999		1.003		1.004		1.035	
Tsf	YP_488472.1	Translation elongatior	1.028		1.188		0.948		1.078	
SelB	YP_491844.1	Selenocysteine-specif	0.806		1.028		0.926		1.136	
InfB	YP_491355.1	Translation initiation :	1.003		1.081		0.943		0.998	
InfC	YP_489980.1	Translation initiation :	1.132	*	1.055		0.815	*	0.831	
InfA	YP_489156.1	Translation initiation :	1.331	*	1.261		0.829		0.785	*
YjgF	YP_492383.1	Putative translation in	1.105	*	0.821	*	1.291	*	0.935	

YoaB	YP_490070.1	Putative translation in	1.07		1.026		1.157		1.141	
YhaR	YP_491302.1	Putative translation in	1.003		0.79	*	1.281	*	1.007	
Tsf	YP_488472.1	Translation elongatior	1.028		1.188		0.948		1.078	
Efp	YP_492290.1	Translation elongatior	1.073		1.135		0.899		0.934	
ZyeiP	YP_490411.1	Translation elongatior	1.007		1.078		0.883	*	1.085	
InfB	YP_491355.1	Translation initiation	1.003		1.081		0.943		0.998	
InfC	YP_489980.1	Translation initiation	1.132	*	1.055		0.815	*	0.831	
InfA	YP_489156.1	Translation initiation	1.331	*	1.261		0.829		0.785	*
PrfB	YP_491092.1	Protein chain release f	0.97		1.199	*	0.797	*	0.998	
PrfC	YP_492504.1	Peptide chain release	0.983		1.116		0.827	*	0.957	
YfcB	YP_490572.1	Methylase of polypep	0.995		1.483		0.659		0.922	
PrfA	YP_489478.1	Protein chain release f	0.774		0.757	*	1.215		1.033	
YchF	YP_489470.1	Predicted GTPase, prc	1.059		0.988		1.051		0.952	

Protein export and secretion system

Preprotein translocase and signal peptidase

SecA	YP_488403.1	Preprotein translocase	0.933		1.038		1.009		1.072	
SecD	YP_488700.1	Preprotein translocase	1.095	*	1.022		1.078		0.856	*
SecB	YP_491824.1	Preprotein translocase	1.327	*	1.257		0.843	*	0.901	
YidC	YP_491729.1	Preprotein translocase	1.211		1.066		0.987		1.016	
YajC	YP_488699.1	Preprotein translocase	1.209	*	1.333	*	0.939		0.968	
SecF	YP_488701.1	Preprotein translocase	1.263		1.017		1.061		0.894	
SecG	YP_491360.1	Preprotein translocase	1.104		1.577		0.569	*	0.769	
SecY	YP_492132.1	Preprotein translocase	1.121		1.045		0.865		0.837	
Ffh	YP_490833.1	Signal recognition par	0.945		0.932		0.902		0.888	
TatA	YP_491606.1	Sec-independent prote	---		---		---		---	
SecA	YP_488403.1	Preprotein translocase	0.933		1.038		1.009		1.072	
SecD	YP_488700.1	Preprotein translocase	1.095	*	1.022		1.078		0.856	*
SecB	YP_491824.1	Preprotein translocase	1.327	*	1.257		0.843	*	0.901	
YidC	YP_491729.1	Preprotein translocase	1.211		1.066		0.987		1.016	
SecF	YP_488701.1	Preprotein translocase	1.263		1.017		1.061		0.894	
SecG	YP_491360.1	Preprotein translocase	1.104		1.577		0.569	*	0.769	
SecY	YP_492132.1	Preprotein translocase	1.121		1.045		0.865		0.837	

Ffh	YP_490833.1	Signal recognition pai	0.945		0.932		0.902		0.888	
FtsY	YP_491970.1	Signal recognition pai	0.925	*	0.954		1.11	*	1.043	
YajC	YP_488699.1	Preprotein translocase	1.209	*	1.333	*	0.939		0.968	
LepB	YP_490796.1	Signal peptidase I	1.121		1.023		1.138		0.981	

Protein recycle proteinase and peptidase

HflB	YP_491363.1	ATP-dependent Zn pr	1.064		1.074		0.915		0.929
Eco	YP_490447.1	Serine protease inhibi	1.405	*	1.066		0.951		0.701
HtrA	YP_488464.1	Trypsin-like serine pro	1.916		1.27	*	1.308	*	0.952
DegQ	YP_491418.1	Trypsin-like serine pro	1.188		0.858		1.25		0.962
DegS	YP_491419.1	Trypsin-like serine pro	---		---		---		---
SohB	YP_489540.1	Periplasmic serine pro	1.003		1.082		1.032		1.103
SppA	YP_490027.1	Periplasmic serine pro	1.311		1.136		1.249		1.074
HycI	YP_490926.1	Ni,Fe-hydrogenase ma	0.728		0.429		1.797		1.048
PtrB	YP_490107.1	Protease II	---		---		---		---

CLP

	ClpB	YP_490816.1	ATPases with chaperc	0.902	*	0.949	1.013		1.141	
	ClpP	YP_488729.1	Protease subunit of A'	0.875	*	0.961	1.114	*	1.144	*
	HslU	YP_491520.1	ATP-dependent prote:	1.01		1.089 *	0.92	*	0.971	
	ClpA	YP_489155.1	ATPases with chaperc	0.963		0.899	1.084		1.016	
	ClpX	YP_488730.1	ATP-dependent prote:	1.134		0.91	0.802		0.581	
	SohB	YP_489540.1	Periplasmic serine prc	1.003		1.082	1.032		1.103	
	HslV	YP_491519.1	ATP-dependent prote:	0.728		0.863	1.239		1.446	*
	SppA	YP_490027.1	Periplasmic serine prc	1.311		1.136	1.249		1.074	
LON										
	YcbZ	YP_489227.1	Predicted ATP-depen(	1.02		0.756	1.381		1.037	
	Lon	YP_488731.1	ATP-dependent Lon p	1.016		1.056	1.007		0.991	
	HslU	YP_491520.1	ATP-dependent prote:	1.01		1.089 *	0.92	*	0.971	
	HslV	YP_491519.1	ATP-dependent prote:	0.728		0.863	1.239		1.446	*
	HtrA	YP_488464.1	Trypsin-like serine pr	1.916		1.27 *	1.308	*	0.952	
	HtpX	YP_490091.1	Zn-dependent proteas	0.63	*	0.619	1.203		1.116	

Peptidase 48

Map	YP_488470.1	Methionine aminopep	1.084		1.132	*	0.953		1.062
YgjD	YP_491256.1	Metal-dependent prot	1.173	*	0.951		1.216	*	0.993
PepN	YP_489204.1	Aminopeptidase N	1.011		1.072		0.923		0.99
NlpD	YP_490951.1	Membrane proteins re	---		---		---		---
Map	YP_488470.1	Methionine aminopep	1.084		1.132	*	0.953		1.062
PepE	YP_492164.1	Peptidase E	0.961		0.651		0.966		0.858
PepN	YP_489204.1	Aminopeptidase N	1.011		1.072		0.923		0.99
PepQ	YP_491597.1	Xaa-Pro aminopeptid	0.778		1.01		0.888		1.047
PepP	YP_491109.1	Xaa-Pro aminopeptid	0.923	*	0.893	*	1.102	*	1.034
MepA	YP_490570.1	Murein endopeptidase	0.91		1.404		0.634		0.763
PepD	YP_488532.1	Di- and tripeptidases	1.062		1.1	*	0.934		0.978
PepN	YP_489204.1	Aminopeptidase N	1.011		1.072		0.923		0.99
PepE	YP_492164.1	Peptidase E	0.961		0.651		0.966		0.858
PepQ	YP_491597 .1	Xaa-Pro aminopeptid	0.778		1.01		0.888		1.047
PepT	YP_489395 .1	Di- and tripeptidases	0.818		0.892	*	0.84	*	0.983
PepA	YP_492398 .1	Leucyl aminopeptidas	0.968		1.08		0.864	*	1.064

SecA	YP_488403.1	Preprotein translocase	0.933		1.038		1.009		1.072	
SecD	YP_488700.1	Preprotein translocase	1.095	*	1.022		1.078		0.856	*
SecB	YP_491824.1	Preprotein translocase	1.327	*	1.257		0.843	*	0.901	
YidC	YP_491729.1	Preprotein translocase	1.211		1.066		0.987		1.016	
YajC	YP_488699.1	Preprotein translocase	1.209	*	1.333	*	0.939		0.968	
SecF	YP_488701.1	Preprotein translocase	1.263		1.017		1.061		0.894	
SecG	YP_491360.1	Preprotein translocase	1.104		1.577		0.569	*	0.769	
secY	YP_492132.1	Preprotein translocase	1.121		1.045		0.865		0.837	

Note: [a] 1-vs-2, 12 h with 0 mM versus 1 mM Mn (II); 3-vs-4, 48 h with 0 mM versus 1 mM Mn (II); 1-vs-3, 12 h versus 48 h with 0 mM Mn (II); and 2-vs-4, 12 h versus 48 h with 1 mM Mn (II). Red value denotes up-regulated expression (>1.2 fold); Green value denotes down-regulated (< 0.833 fold).

[b] Sig, significance.

[c] *, p -values < 0.05 and the up-regulated value >1.2 fold / down-regulated value < 0.833 fold.

Table S5. All of the signal transduction related proteins.[a−c]

Protein or Domain	Accession	Desription	1-VS-2	Sig	3-VS-4	Sig	1-VS-3	Sig	2-VS-4	Sig
AceK	YP_492159.1	isocitrate dehydrogenase kinase/phosphatase	1.065		1.014		1.338		1.242	
ArcA	YP_492531.1	DNA-binding response regulator in two-component regulatory system with ArcB or CpxA	1.114		1.034		0.962		0.978	
ArgT	YP_490552.1	lysine/arginine/ornithine transporter subunit	1.218	*	0.822	*	1.952	*	1.264	*
ArtI	YP_489136.1	arginine transporter subunit	1.298	*	0.947		1.414		0.972	
BaeR	YP_490321.1	DNA-binding response regulator in two-component regulatory system with BaeS	1.023		1.099		0.835		0.962	
BolA	YP_488727.1	regulator of penicillin binding proteins and beta lactamase transcription (morphogene)	0.897		0.755		2.542	*	1.843	
CpxA	YP_491539.1	sensory histidine kinase in two-component regulatory system with CpxR	1.005		1.143		0.993		0.903	
CpxR	YP_491538.1	DNA-binding response regulator in two-component regulatory system with CpxA	0.875		1.049		0.724	*	0.879	
Crp	YP_492074.1	DNA-binding transcriptional dual regulator	0.943		1.035		0.875		1.027	
CsrA	YP_490905.1	pleiotropic regulatory protein for carbon source metabolism	1.174	*	1.101		1.146		1.135	
CstA	YP_488887.1	carbon starvation protein	0.924		0.929		1.002		0.912	
DksA	YP_488448.1	transcriptional regulator of rRNA transcription, DnaK suppressor protein	1.092		0.97		0.96		0.913	
Gltl	YP_488946.1	glutamate and aspartate transporter subunit	1.301	*	0.838	*	1.308	*	0.834	

PhoP	YP_489398.1	DNA-binding response regulator in two-component regulatory system with PhoQ	0.629	*	0.615	*	1.097		1.022	
UvrY	YP_490171.1	DNA-binding response regulator in two-component regulatory system with BarA	0.968		0.822		1.027		0.737	*
RcsD	YP_490454.1	phosphotransfer intermediate protein in two-component regulatory system with RcsBC	0.993		1.034		1.127		1.132	
EvgA	YP_490611.1	DNA-binding response regulator in two-component regulatory system with EvgS	0.713	*	0.81	*	0.926		1.122	
Ffh	YP_490833.1	Signal Recognition Particle (SRP) component with 4.5S RNA (ffs)	0.945		0.932		0.902		0.888	
FliY	YP_490177.1	cystine transporter subunit	1.027		0.804	*	1.474	*	1.115	*
Fnr	YP_489604.1	DNA-binding transcriptional dual regulator	1.012		0.889		0.984		0.856	
FtsY	YP_491970.1	fused Signal Recognition Particle (SRP) receptor	0.925	*	0.954		1.11	*	1.043	
GlnE	YP_491245.1	fused deadenylyltransferase and adenylyltransferase for glutamine synthetase	0.907		1.036		1.165		0.927	
GlnG	YP_491582.1	fused DNA-binding response regulator in two-component regulatory system with GlnL, nitrogen	0.965		0.875		0.98		0.87	*
GlnH	YP_489084.1	glutamine transporter subunit	1.637	*	1.138		1.037		0.701	*
GlnL	YP_491581.1	sensory kinase in two-component regulatory system with GlnG	1.06		0.687		1.124		0.789	
QseC	YP_491218.1	sensory histidine kinase in two-component regulatory system with QseB	0.879		0.899		1.129		1.24	*
HisJ	YP_490551.1	histidine/lysine/arginine/ornithine transporter subunit	1.371	*	0.876		1.14	*	0.715	*
LepB	YP_490796.1	leader peptidase	1.121		1.023		1.138		0.981	

LexA	YP_492186.1	DNA-binding transcriptional repressor	1.086		0.852		0.83		0.717	*
LuxS	YP_490901.1	S-ribosylhomocysteinase	1.379	*	0.947		1.235	*	0.86	*
NarL	YP_489491.1	DNA-binding response regulator in two-component regulatory system with NarX (or NarQ)	0.85		0.957		0.893		0.884	
NarP	YP_490431.1	DNA-binding response regulator in two-component regulatory system with NarQ or NarX	0.908		0.858	*	0.74		0.773	*
OmpR	YP_492027.1	DNA-binding response regulator in two-component regulatory system with EnvZ	0.925		1.189		0.835		0.986	
QseB	YP_491217.1	DNA-binding response regulator in two-component regulatory system with QseC	1.106		1.558		0.88		1.227	*
ProQ	YP_490093.1	structural transport element	0.89		0.978		0.653		0.789	
PspA	YP_489572.1	regulatory protein for phage-shock-protein operon	1.286	*	2.761	*	0.964		2.095	*
PtsN	YP_491389.1	PTS system sugar-specific transporter subunit IIA	1.045		1.092		0.855		0.897	
PtsP	YP_491034.1	fused PEP-protein phosphotransferase (enzyme I) of PTS system	0.991		0.879	*	1.15	*	1.064	
RcsB	YP_490455.1	DNA-binding response regulator in two-component regulatory system with RcsC and YojN	1.032		1.073		0.803	*	0.887	
RelA	YP_490992.1	(p)ppGpp synthetase I/GTP pyrophosphokinase	1.059		0.991		1.059		0.999	
RseB	YP_490799.1	anti-sigma factor	0.999		0.892		1.078		1.035	
Rtn_2	YP_490416.1	hypothetical protein Y75_p2139	1.316		0.757		1.467		0.835	
SixA	YP_490582.1	phosphohistidine phosphatase	1.517		1.076		1.477		1.037	

SpoT	YP_491784.1	bifunctional (p)ppGpp synthetase II/guanosine-3~,5~-bis pyrophosphate 3~-pyrophosphohydrolase	1.161	*	0.916		1.375	*	1.099	
Spy	YP_490004.1	envelope stress induced periplasmic protein	1.174	*	1.614	*	0.969		1.501	*
UspA	YP_491940.1	universal stress global response regulator	0.977		0.997		1.131	*	1.181	*
YbdQ	YP_488897.1	universal stress protein UP12	0.764		0.858	*	0.943		1.121	
YbeZ	YP_488951.1	hypothetical protein Y75_p0650	1.057		1.06		0.95		0.893	
YcgF_2	YP_489430.1	FAD-binding phosphodiesterase	0.852		0.92		1.117	*	1.33	
YdaA	YP_489603.1	stress-induced protein	0.901		1.202		0.888	*	1.148	
YeaG	YP_490044.1	hypothetical protein Y75_p1758	0.989		1.072		2.143		2.24	
YebR	YP_490094.1	hypothetical protein Y75_p1808	0.954		0.899		1.112		1.082	
YfhA	YP_490782.1	DNA-binding response regulator in two-component system	0.722		0.827	*	1.263		1.243	
YgeV	YP_491071.1	DNA-binding transcriptional regulator	1.042		1.053		0.967		0.964	
YgiM	YP_491247.1	signal transduction protein	1.681	*	1.563		1.009		1.046	
YiiT	YP_491528.1	stress-induced protein	0.85	*	1.216	*	0.652	*	0.991	
YjiY	YP_492485.1	inner membrane protein	1.607	*	1.105		0.984		0.862	
YnaF	YP_489644.1	stress-induced protein, ATP-binding protein	1.275		1.216		1.115	*	1.016	

YtfE	YP_492351.1	regulator of cell morphogenesis and cell wall metabolism	0.663	1.002		0.741		1.109
ArcB	YP_491395.1	hybrid sensory histidine kinase in two-component regulatory system with ArcA	1.009	0.768	*	1.147	*	0.884
BarA	YP_490994.1	hybrid sensory histidine kinase, in two-component regulatory system with UvrY	1.383	1.433	*	0.977		1.002
YihK	YP_491579.1	GTP-binding protein	1.035	1.085	*	1		1.032

Note: [a] 1-vs-2, 12 h with 0 mM versus 1 mM Mn (II); 3-vs-4, 48 h with 0 mM versus 1 mM Mn (II); 1-vs-3, 12 h versus 48 h with 0 mM Mn (II); and 2-vs-4, 12 h versus 48 h with 1 mM Mn (II). Red value denotes up-regulated expression (>1.2 fold); Green value denotes down-regulated (< 0.833 fold).

[b] Sig, significance.

[c] *, p -values < 0.05 and the up-regulated value >1.2 fold / down-regulated value < 0.833 fold.

Table S6. All of the inorganic ion transport and metabolism related proteins.[a−c]

Protein or Domain	Accession	Desription	1-vs-2	Sig	3-vs-4	Sig	1-vs-3	Sig	2-vs-4	Sig
PspE	YP_489576.1	thiosulfate:cyanide sulfurtransferase	2.168	*	0.76		0.656		0.187	*
FeoB	YP_492023.1	fused ferrous iron transporter, protein B	2.335		0.897		0.999		0.659	
PstS	YP_491701.1	phosphate transporter subunit	1.73	*	1.331	*	0.95		0.674	*
ApaG	YP_488356.1	protein associated with Co2+ and Mg2+ efflux	1.142		0.923		0.943		0.754	
YcdO	YP_489289.1	hypothetical protein Y75_p0990	1.762	*	1.38		1.211		0.784	
YdeN	YP_489763.1	hypothetical protein Y75_p1474	1.733		0.844		1.519		0.787	
Zkup	YP_491682.1	potassium transporter	1.189		0.69	*	1.427		0.82	
CysA	YP_490658.1	sulfate/thiosulfate transporter subunit	0.954		0.792		1.168		0.851	
NrfA	YP_492213.1	nitrite reductase, formate-dependent, cytochrome	0.724	*	0.813		0.706	*	0.853	
CorA	YP_491626.1	magnesium/nickel/cobalt transporter	1.126		0.894		1.167		0.861	
Fiu	YP_489078.1	iron outer membrane transporter	2.484	*	1.879		1.672	*	0.861	
YibN	YP_491822.1	rhodanese-related sulfurtransferase	1.23	*	0.974		1.045		0.875	*
YfeX	YP_490667.1	hypothetical protein Y75_p2392	0.925	*	0.874		0.872	*	0.887	

YnjE	YP_490018.1	thiosulfate sulfur transferase	0.821	*	0.866		0.918		0.906	*
YaeC	YP_488500.1	DL-methionine transporter subunit	1.24		0.994		1.139		0.923	
MdoG	YP_489317.1	glucan biosynthesis protein, periplasmic	1.289	*	1.123		1.051		0.93	
YfgD	YP_490723.1	oxidoreductase	1.138		1.18		1.069		0.933	
Zur	YP_492189.1	DNA-binding transcriptional activator	1.027		1.134		0.898		0.934	
YhjA	YP_491917.1	cytochrome C peroxidase	1.012		1.217		0.794		0.945	
Ftn	YP_490165.1	cytoplasmic ferritin iron storage protein	0.487	*	0.63		0.715	*	0.951	
YdcG	YP_489690.1	glucan biosynthesis protein, periplasmic	0.817	*	0.846	*	0.954		0.955	
Fur	YP_488963.1	DNA-binding transcriptional dual regulator	1.069		1.118		1.016		0.956	
YadF	YP_488429.1	carbonic anhydrase	1.398	*	1.21	*	1.056		0.961	
SodA	YP_491542.1	superoxide dismutase, Mn	2.036	*	1.756	*	1.144		0.967	
ZntA	YP_491965.1	zinc, cobalt and lead efflux system	0.843		0.788		1.013		0.975	
YdaN	YP_489612.1	Zn(II) transporter	0.915		0.93		0.88		0.979	
CyaY	YP_491635.1	frataxin, iron-binding and oxidizing protein	0.943		0.824		1.148		0.986	
YbeX	YP_488949.1	ion transport protein	0.75	*	0.734	*	0.894		0.995	

PhnA	YP_492251.1	phosphonate metabolizing protein	1.103	1.581		0.715		1.006	
KatG	YP_491509.1	catalase/hydroperoxidase HPI(I)	1.002	1.062		0.943		1.007	
NarK	YP_489493.1	nitrate/nitrite transporter	1.098	0.722		1.392		1.026	
YbaR	YP_488775.1	copper transporter	1.11	0.976		1.199		1.034	
Abc	YP_488502.1	DL-methionine transporter subunit	1.004	1.536		0.684		1.036	
ModA	YP_489036.1	molybdate transporter subunit	0.913	0.524	*	1.885		1.036	
YbaL	YP_488769.1	transporter with NAD(P)-binding Rossmann-fold domain	0.863	0.765	*	1.183	*	1.044	
TrkA	YP_492143.1	NAD-binding component of TrK potassium transporter	0.947	0.876		1.153		1.056	
OppD	YP_489514.1	oligopeptide transporter subunit	0.916	0.917	*	1.115	*	1.069	
CirA	YP_490394.1	ferric iron-catecholate outer membrane transporter	2.595	2.558	*	1.41		1.072	
SseA	YP_490749.1	3-mercaptopyruvate sulfurtransferase	0.973	0.687		1.479	*	1.097	*
FhuA	YP_488453.1	ferrichrome outer membrane transporter	1.293	0.818		1.595	*	1.117	
Ppk	YP_490729.1	polyphosphate kinase, component of RNA degradosome	0.94	0.933		1.068		1.124	*
Dps	YP_489085.1	Fe-binding and storage protein	0.381	0.379	*	1.185	*	1.13	
PitA	YP_491942.1	phosphate transporter, low-affinity	1.386	1.462		1.085	*	1.133	

YheM	YP_492088.1	intraceullular sulfur oxidation protein	1.426		0.635		2.592		1.142	
CysP	YP_490661.1	thiosulfate transporter subunit	1.076		0.851	*	1.521	*	1.178	*
CysN	YP_490960.1	sulfate adenylyltransferase, subunit 1	0.637	*	0.812	*	1.056		1.193	
SodB	YP_489920.1	superoxide dismutase, Fe	0.677		0.999		0.85		1.202	*
YccK	YP_489241.1	sulfite reductase subunit	0.698	*	0.982		0.879		1.218	
NlpA	YP_491774.1	cytoplasmic membrane lipoprotein-28	0.742	*	0.779		1.133		1.271	
CutC	YP_490136.1	copper homeostasis protein	0.98		0.733	*	1.382		1.313	
CysQ	YP_492356.1	PAPS (adenosine 3~-phosphate 5~-phosphosulfate) 3~(2~),5~-bisphosphate	1.334		0.902		1.887	*	1.362	
CysI	YP_490972.1	sulfite reductase subunit beta	0.635		0.78	*	1.089	*	1.371	*
CysJ	YP_490973.1	sulfite reductase subunit alpha, flavoprotein	0.763		0.845		1.293		1.47	
KatE	YP_489993.1	hydroperoxidase HPII(III)	0.874	*	1.155	*	1.19	*	1.599	
Bfr	YP_492096.1	bacterioferritin, iron storage and detoxification protein	0.735	*	0.878	*	1.375	*	1.604	*

Note: [a] 1-vs-2, 12 h with 0 mM versus 1 mM Mn (II); 3-vs-4, 48 h with 0 mM versus 1 mM Mn (II); 1-vs-3, 12 h versus 48 h with 0 mM Mn (II); and 2-vs-4, 12 h versus 48 h with 1 mM Mn (II). Red value denotes up-regulated expression (>1.2 fold); Green value denotes down-regulated (< 0.833 fold).

[b] Sig, significance.

[c] *, *p* -values < 0.05 and the up-regulated value >1.2 fold / down-regulated value < 0.833 fold.

Table S7. All of the metabolism pathway-related proteins.[a–c]

Protein or Domain	Accession	Desription	1-vs-2 [a]	Sig [b]	3-vs-4	Sig	1-vs-3	Sig	2-vs-4	Sig
**Pyruvate metabolism related proteins**										
Acs	YP_492212.1	bifunctional acetyl-CoA synthetase/propionyl-CoA synthetase	0.864		0.999		1.114		1.392	*
AldB	YP_491846.1	aldehyde dehydrogenase B	0.857	* [c]	0.866	*	1.81	*	1.897	
PflB	YP_489175.1	pyruvate formate lyase I	0.911		1.081		0.853		0.977	
AceE	YP_488417.1	pyruvate dehydrogenase, decarboxylase component E1, thiamin-binding	0.903		1.04		0.872		1.018	
AceF	YP_488418.1	pyruvate dehydrogenase, dihydrolipoyltransacetylase component E2	1.119		1.04		1.096		1	
Lpd	YP_488419.1	lipoamide dehydrogenase, E3 component is part of three enzyme complexes	1.034		1.077		0.976		1.06	
AdhE	YP_489507.1	bifunctional acetaldehyde-CoA dehydrogenase and iron-dependent alcohol dehydrogenase and pyruvate-formate lyase	1.029		1.008		1.02		0.991	
PckA	YP_492029.1	phosphoenolpyruvate carboxykinase	0.944		1.054		0.826		0.938	
PtsI	YP_490652.1	PEP-protein phosphotransferase of PTS system	0.879		1.006		0.954		1.135	
PykA	YP_490116.1	pyruvate kinase II	0.967		0.948		1.007		0.985	
PykF	YP_489938.1	pyruvate kinase I	0.954		0.995		0.975		1.071	
SucB	YP_489006.1	dihydrolipoyltranssuccinase	0.88		1.016		0.939		0.983	

PpsA	YP_489964.1	phosphoenolpyruvate synthase	0.991		1.11		0.978		1.071	
SthA	YP_491490.1	pyridine nucleotide transhydrogenase, soluble	0.972		1.055		0.852		0.946	
Ppc	YP_491496.1	phosphoenolpyruvate carboxylase	1.053		0.909		1.046		0.936	
YfiD	YP_490807.1	pyruvate formate lyase subunit	1.081		0.853		0.758	*	0.548	*
YcdW	YP_489300.1	2-ketoacid reductase	0.841		1.001		0.888		1.081	*
SseA	YP_490749.1	3-mercaptopyruvate sulfurtransferase	0.973		0.687		1.479	*	1.097	
Gor	YP_491935.1	glutathione oxidoreductase	0.934		0.98		1.113		1.091	*
TdcE	YP_491303.1	pyruvate formate-lyase 4/2-ketobutyrate formate-lyase	0.95		0.853		0.822	*	0.786	
PtsP	YP_491034.1	fused PEP-protein phosphotransferase (enzyme I) of PTS system	0.991		0.879	*	1.15	*	1.064	
YiaE	YP_491883.1	2-keto-D-gluconate reductase	1.02		1.074		1.133		1.056	
Dxs	YP_488712.1	1-deoxyxylulose-5-phosphate synthase, thiamine-requiring, FAD-requiring	1.095		1.065		0.948		0.933	*
YdbK	YP_489646.1	fused Fe-S subunit of pyruvate-flavodoxin oxidoreductase	0.997		0.903		0.993		0.87	*
YnjE	YP_490018.1	thiosulfate sulfur transferase	0.821	*	0.866		0.918		0.906	
YfdZ	YP_490621.1	aminotransferase, PLP-dependent	1.005		0.757	*	1.249		0.947	*
PoxB	YP_489144.1	pyruvate dehydrogenase (pyruvate oxidase), thiamin-dependent, FAD-binding	0.74	*	1.128	*	1.15	*	1.716	

YccX	YP_489240.1	acylphosphatase	0.732		1.567	*	0.814		1.725	
IlvB	YP_491763.1	acetolactate synthase I, large subunit	0.754	*	0.536	*	1.245	*	0.972	*
YfbQ	YP_490532.1	aminotransferase	0.872		1.127		0.981		1.162	
PflA	YP_489174.1	pyruvate formate lyase activating enzyme 1	0.85		1.09		0.717	*	1.025	*
YdiA	YP_489965.1	hypothetical protein Y75_p1678	0.906		0.902		1.088		1.178	
YcgC_3	YP_489465.1	fused dihydroxyacetone-specific PTS enzyme HPr component and EI component	1.15		1.053		0.975		0.913	
YbiW	YP_489096.1	pyruvate formate lyase	0.758		1.443		0.757	*	1.426	
PdhR	YP_488416.1	DNA-binding transcriptional dual regulator	0.623		0.612		1.125		1.082	
YcgM	YP_489447.1	isomerase/hydrolase	1.06		1.373		0.893		1.206	
PtsA_1	YP_491504.1	fused PTS enzymes Hpr component, enzyme I component, and enzyme IIA component	1.077		0.832	*	1.261	*	1.009	
UbiC	YP_492182.1	chorismate pyruvate lyase	0.837		0.48		1.379		0.783	
YkgC	YP_488600.1	oxidoreductase with FAD/NAD(P)-binding domain and dimerization domain	0.645		0.837		0.997		1.28	
YgbM	YP_490948.1	hypothetical protein Y75_p2677	---		---		---		---	
IlvI	YP_488383.1	acetolactate synthase III, large subunit	1.295		1.164		1.089		0.969	
PrpB	YP_488626.1	2-methylisocitrate lyase	1.087		0.682	*	5.738	*	3.627	

PrpC	YP_488627.1	2-methylcitrate synthase	0.815	0.566 *	5.056	3.659
PrpD	YP_488628.1	2-methylcitrate dehydratase	0.778	0.665 *	7.08	3.758

Fatty acid metabolism related proteins

Aas	YP_491041.1	bifunctional 2-acylglycerophospho-ethanolamine acyl transferase and acyl-acyl carrier protein synthetase	0.94	0.751	1.239	1.004
AccA	YP_488487.1	acetylCoA carboxylase, carboxytransferase subunit alpha	1.001	0.941	1.051	0.964 *
AldB	YP_491846.1	aldehyde dehydrogenase B	0.857 *	0.866 *	1.81 *	1.897
AldH	YP_489568.1	gamma-Glu-gamma-aminobutyraldehyde dehydrogenase, NAD(P)H-dependent	0.487	0.613 *	0.836 *	0.98 *
AstD	YP_490007.1	succinylglutamic semialdehyde dehydrogenase	0.908 *	0.838 *	1.91 *	1.726 *
FabA	YP_489226.1	beta-hydroxydecanoyl thioester dehydrase	0.89 *	0.928	1.235 *	1.248
FabB	YP_490565.1	3-oxoacyl-[acyl-carrier-protein] synthase I	1.112	1.18	1.156 *	1.193
FabD	YP_489360.1	malonyl-CoA-[acyl-carrier-protein] transacylase	1.04	1.067	1.195 *	1.241
FabF	YP_489363.1	3-oxoacyl-[acyl-carrier-protein] synthase II	1.031	0.873 *	1.115 *	0.983 *
FabG	YP_489882.1	7alpha-hydroxysteroid dehydrogenase qiangjinneiguc	0.908 *	1.115 *	1.366 *	1.714
FabH	YP_489359.1	3-oxoacyl-[acyl-carrier-protein] synthase III	1.042	0.999	1.134	1.084

FabI	YP_489556.1	enoyl-[acyl-carrier-protein] reductase	1.07	0.98	1.061 *	1.046 *
FabZ	YP_488482.1	(3R)-hydroxymyristol acyl carrier protein dehydratase	1.23	1.093 *	1.189 *	1.098 *
FadA	YP_491599.1	3-ketoacyl-CoA thiolase	0.965	0.869	1.552	1.442
FadD	YP_490066.1	acyl-CoA synthetase	0.912	0.81	1.687	1.533
FolE	YP_490392.1	GTP cyclohydrolase I	1.034	0.97	1.053	0.964
GlyA	YP_490779.1	serine hydroxymethyltransferase	0.846	1.073	0.874	1.096 *
HdhA	YP_489882.1	7alpha-hydroxysteroid dehydrogenase	0.908 *	1.115 *	1.366 *	1.714
Ndh	YP_489377.1	respiratory NADH dehydrogenase 2/cupric reductase	1.315	0.835 *	1.139	0.89
PaaG	YP_489663.1	acyl-CoA hydratase	1.121	1.318	0.8	0.931
Qor	YP_492194.1	quinone oxidoreductase, NADPH-dependent	1.026	1.139 *	1.287	1.319
SrlD	YP_490914.1	sorbitol-6-phosphate dehydrogenase	1.538 *	1.073	1.347 *	0.88
UcpA	YP_490662.1	oxidoredutase, sulfate metabolism protein	0.854 *	0.919	1.009	1.105
YafB	YP_488504.1	2,5-diketo-D-gluconate reductase B	0.81	0.65	1.333 *	0.913 *
YafH	YP_488518.1	acyl coenzyme A dehydrogenase	1.002	1.027	1.641 *	1.63 *
YahK	YP_488620.1	oxidoreductase	0.865 *	1.173	1.145 *	1.299

YeaE	YP_490042.1	oxidoreductase	1.242	1.332	1.153	1.223
YgfF	YP_491103.1	NAD(P)-binding oxidoreductase with NAD(P)-binding Rossmann-fold domain	1.108	1.198	1.178	1.167 *
YghA	YP_491198.1	glutathionylspermidine synthase, with NAD(P)-binding Rossmann-fold domain	1.118	1.108	2.177	1.961 *
YohF	YP_490376.1	oxidoreductase with NAD(P)-binding Rossmann-fold domain	0.989	1.053	1.439 *	1.516
YqeF	YP_491049.1	acyltransferase	1.537 *	1.252	1.213	1.087

TCA cycle related proteins

AcnA	YP_489544.1	aconitate hydratase 1	0.752	0.815	1.045	1.117
Apt	YP_488760.1	adenine phosphoribosyltransferase	1.157 *	1.239 *	0.861 *	0.922
AspA	YP_492282.1	aspartate ammonia-lyase	0.857 *	0.921	0.788	0.83
Dxs	YP_488712.1	1-deoxyxylulose-5-phosphate synthase, thiamine-requiring, FAD-requiring	1.095	1.065	0.948	0.933
EntC	YP_488882.1	isochorismate synthase 1	---	---	---	---
FrdA	YP_492299.1	fumarate reductase (anaerobic) catalytic and NAD/flavoprotein subunit	0.621	0.623	1.042	1.076 *
FrdB	YP_492298.1	fumarate reductase (anaerobic), Fe-S subunit	0.894 *	0.566 *	1.138 *	0.742
FumC	YP_489874.1	fumarate hydratase (fumarase C), aerobic Class II	0.871 *	1.135 *	0.891	1.099

GalK	YP_489030.1	galactokinase	1.228	0.904	1.109	0.882
GltA	YP_489000.1	citrate synthase	0.843	0.868	0.909	1.022
Icd	YP_489404.1	isocitrate dehydrogenase,-specific for NADP+	0.872	0.875	1.007	1.059
Lpd	YP_488419.1	lipoamide dehydrogenase, E3 component is part of three enzyme complexes	1.034	1.077	0.976	1.06 *
ManA	YP_489876.1	mannose-6-phosphate isomerase	0.962	1.201	0.842	0.907
Mdh	YP_491420.1	malate dehydrogenase	1.041	0.838	1.077	0.875
MenF	YP_490505.1	isochorismate synthase 2	0.856	0.951	1.007	1.12
PckA	YP_492029.1	phosphoenolpyruvate carboxykinase	0.944	1.054	0.826	0.938
PrpC	YP_488627.1	2-methylcitrate synthase	0.815	0.566 *	5.056	3.659
SdhA	YP_489003.1	succinate dehydrogenase, flavoprotein subunit	0.992	0.785	1.237	0.954
SdhB	YP_489004.1	succinate dehydrogenase, FeS subunit	0.96	0.773 *	1.189	1.006
SthA	YP_491490.1	pyridine nucleotide transhydrogenase, soluble	0.972	1.055	0.852	0.946
SucA	YP_489005.1	2-oxoglutarate decarboxylase, thiamin-requiring	0.897	0.936	0.97	0.998
SucB	YP_489006.1	dihydrolipoyltranssuccinase	0.88	1.016	0.939	0.983
SucC	YP_489007.1	succinyl-CoA synthetase subunit beta	0.965	1.05	1.001	1.056

SucD	YP_489008.1	succinyl-CoA synthetase subunit alpha	0.942	1.085	0.939		1.06	
YbjU	YP_489143.1	L-allo-threonine aldolase, PLP-dependent	0.843	1.041	0.989		1.206	
YkgC	YP_488600.1	oxidoreductase with FAD/NAD(P)-binding domain and dimerization domain	0.645	0.837	0.997		1.28	

Amino acid transport and metabolism

TnaA	YP_491725.1	tryptophanase/L-cysteine desulfhydrase, PLP-dependent	0.926	1.091	0.815		0.964	
oppA	YP_489511.1	oligopeptide transporter subunit	1.069	1.233	0.873		0.978	
GcvP	YP_491104.1	glycine decarboxylase, PLP-dependent, subunit (protein P) of glycine cleavage complex	1.049	0.983	1.086		1.007	
CarB	YP_488339.1	carbamoyl-phosphate synthase large subunit	0.978	1.094	0.846		0.935	
CysK	YP_490650.1	cysteine synthase A, O-acetylserine sulfhydrolase A subunit	0.859	1.042	1.165		1.444	
Acs	YP_492212.1	Abifunctional acetyl-CoA synthetase/propionyl-CoA synthetase	0.864	0.999	1.114		1.392	
FabI	YP_489556.1	enoyl-[acyl-carrier-protein] reductase	1.07	0.98	1.061	*	1.046	
FabB	YP_490565.1	3-oxoacyl-[acyl-carrier-protein] synthase I	1.112	1.18	1.156	*	1.193	
YgeY	YP_491074.1	peptidase	0.55	0.79	0.684		0.924	*
GlnA	YP_491580.1	glutamine synthetase	1.009	1.083	0.853		0.925	
YgfK_2	YP_491080.1	oxidoreductase, Fe-S subunit	0.453	0.533	0.384		0.425	

PepN	YP_489204.1	aminopeptidase N	1.011		1.072		0.923		0.99
GlyA	YP_490779.1	serine hydroxymethyltransferase	0.846		1.073		0.874		1.096
AspA	YP_492282.1	aspartate ammonia-lyase	0.857	*	0.921		0.788		0.83
Dcp	YP_489801.1	dipeptidyl carboxypeptidase II	0.837	*	0.902	*	1.152	*	1.307
GcvT	YP_491106.1	glycine cleavage complex aminomethyltransferase	1.012		1.045		0.995		1.033
PepD	YP_488532.1	aminoacyl-histidine dipeptidase	1.062		1.1	*	0.934		0.978
YfhO	YP_490758.1	cysteine desulfurase (tRNA sulfurtransferase), PLP-dependent	1.196	*	1.205		1.374		1.388
AspC	YP_489200.1	aspartate aminotransferase, PLP-dependent	0.895	*	0.964		0.888	*	0.996
SpeA	YP_491137.1	biosynthetic arginine decarboxylase, PLP-binding	0.86		0.914		0.867		0.942
AnsB	YP_491156.1	periplasmic L-asparaginase II	1.01		0.622	*	1.099	*	0.643
DppA	YP_491891.1	dipeptide transporter	1.021		1.036		1.398	*	1.389
PrsA	YP_489474.1	phosphoribosylpyrophosphate synthase	0.986		0.964		1.045		1.002
PepB	YP_490751.1	aminopeptidase B	0.973		0.748		1.207	*	1.003
PepQ	YP_491597.1	proline dipeptidase	0.778		1.01		0.888		1.047
FadB_2	YP_491598.1	bifunctional 3-hydroxybutyryl-CoA epimerase/delta(3)-cis-delta(2)-trans-enoyl-CoA isomerase/enoyl-CoA hydratase and 3-	0.822	*	0.936		1.262	*	1.417

PyrG	YP_490988.1	CTP synthetetase	0.81	*	0.941		0.745		0.9	*
GlnH	YP_489084.1	glutamine transporter subunit	1.637	*	1.138		1.037		0.701	
IadA	YP_492462.1	isoaspartyl dipeptidase	0.86	*	0.7		1.395		1.077	
PepP	YP_491109.1	proline aminopeptidase P II	0.923	*	0.893	*	1.102	*	1.034	
GdhA	YP_490022.1	glutamate dehydrogenase, NADP-specific	0.993		1.095		1.058		1.143	
OppF	YP_489515.1	oligopeptide transporter subunit	0.861		1.096		0.848		1.031	*
FliY	YP_490177.1	cystine transporter subunit	1.027		0.804	*	1.474	*	1.115	*
SerC	YP_489179.1	3-phosphoserine/phosphohydroxythreonine aminotransferase	0.996		1.109	*	1.037		1.187	*
YcdW	YP_489300.1	2-ketoacid reductase	0.841		1.001		0.888		1.081	
YeaU	YP_490061.1	dehydrogenase	1.019		0.93		0.881	*	0.855	
PrlC	YP_491937.1	oligopeptidase A	1.034		0.989		1.077		0.991	*
CarA	YP_488338.1	carbamoyl phosphate synthetase small subunit, glutamine amidotransferase	1.093	*	0.971		0.995		0.9	
YgeW	YP_491072.1	hypothetical protein Y75_p2803	0.534		0.888		0.592		0.996	
NanA	YP_491409.1	N-acetylneuraminate lyase	1.091		1.147	*	0.888	*	0.96	*
SelD	YP_490025.1	selenophosphate synthase	1.131	*	1.185	*	1.216	*	1.233	

CstC	YP_490009.1	succinylornithine transaminase, PLP-dependent	0.866	*	0.825		2.301	*	2.008	
ProC	YP_488679.1	pyrroline-5-carboxylate reductase	1.076		0.982		1.087		0.952	
GltI	YP_488946.1	glutamate and aspartate transporter subunit	1.301	*	0.838	*	1.308	*	0.834	*
DadA	YP_489456.1	D-amino acid dehydrogenase	0.881		0.694	*	1.047		0.751	
GabT	YP_490877.1	4-aminobutyrate aminotransferase, PLP-dependent	1.069	*	0.9	*	1.663	*	1.455	
Tdh	YP_491817.1	threonine 3-dehydrogenase	0.957		1.215	*	0.815	*	1.009	
Asd	YP_492000.1	aspartate-semialdehyde dehydrogenase	1.204	*	1.003		1.094		0.979	
YbjU	YP_489143.1	L-allo-threonine aldolase, PLP-dependent	0.843		1.041		0.989		1.206	
DapA	YP_490706.1	dihydrodipicolinate synthase	0.97		0.825	*	1.177	*	0.976	*
GoaG	YP_489570.1	GABA aminotransferase, PLP-dependent	0.665	*	0.603	*	0.905	*	0.866	
GltB	YP_491397.1	glutamate synthase, large subunit	0.922	*	0.833	*	0.875	*	0.83	
DapD	YP_488468.1	2,3,4,5-tetrahydropyridine-2-carboxylate N-succinyltransferase	1.016		1.076		1.052		1.054	
ProX	YP_490894.1	glycine betaine transporter subunit	1.708		1.068		1.297	*	0.789	
ThrA	YP_488308.1	fused aspartokinase I and homoserine dehydrogenase I	0.69		0.83	*	0.927		1.028	
GatD	YP_490329.1	galactitol-1-phosphate dehydrogenase, Zn-dependent and NAD(P)-binding	1.029		1.101		0.773	*	0.89	*

AcpD	YP_489679.1	NADH-azoreductase, FMN-dependent	1.021		1.281	*	1.276	*	1.788	
ThrC	YP_488310.1	threonine synthase	0.841	*	0.882	*	0.966		0.973	
SelA	YP_491843.1	selenocysteine synthase	0.746	*	0.85	*	0.938		1.027	
SpeB	YP_491136.1	agmatinase	0.986		0.951		1.151	*	1.123	*
UspA	YP_491940.1	universal stress global response regulator	0.977		0.997		1.131	*	1.181	
OxyR	YP_491491.1	DNA-binding transcriptional dual regulator	0.964		0.903		0.864		0.877	
YbiB	YP_489073.1	transferase/phosphorylase	0.913		0.87	*	1.062		1.015	
FadD	YP_490066.1	acyl-CoA synthetase	0.912		0.81		1.687		1.533	*
TolC	YP_491227.1	transport channel	1.33	*	0.927		1.761	*	1.224	
PotD	YP_489391.1	polyamine transporter subunit	1.25		1.132		1.115		0.964	
PepT	YP_489395.1	peptidase T	0.818		0.892	*	0.84	*	0.983	
PdxB	YP_490562.1	erythronate-4-phosphate dehydrogenase	0.927	*	1.04		0.877		0.989	
DapB	YP_488337.1	dihydrodipicolinate reductase	0.942		0.97		1.006		1.197	
AroG	YP_489027.1	3-deoxy-D-arabino-heptulosonate-7-phosphate synthase, phenylalanine repressible	1.169	*	0.924		1.065		0.954	*
AsnA	YP_491685.1	asparagine synthetase A	1.124		1.039		0.918		0.854	*

ProA	YP_488538.1	gamma-glutamylphosphate reductase	1.01		1.186		1.187	*	1.208	*
YcjK	YP_489565.1	gamma-Glu-putrescine synthase	0.655	*	0.762	*	0.67	*	0.774	
SpeE	YP_488424.1	spermidine synthase	0.801	*	0.688	*	1.438	*	1.313	
ProV	YP_490892.1	glycine betaine transporter subunit	1.454	*	1.303	*	1.013		0.87	
ZilvE	YP_491668.1	branched-chain amino-acid aminotransferase	0.895		0.842		1.149		1.131	*
ArgT	YP_490552.1	lysine/arginine/ornithine transporter subunit	1.218	*	0.822	*	1.952	*	1.264	*
OppB	YP_489512.1	oligopeptide transporter subunit	0.982		1.019		1.14	*	1.102	
MsbA	YP_489186.1	lipid ABC transporter ATP-binding protein/membrane protein	1.164	*	0.962		1.327	*	0.993	
ZaroK	YP_492042.1	shikimate kinase I	1.021		0.803		1.098		0.919	
YbiT	YP_489093.1	ABC transporter ATP-binding protein	0.921		0.936		1.094	*	1.017	*
PotA	YP_489394.1	polyamine transporter subunit	0.989		0.841	*	1.257	*	1.306	
AsnB	YP_488954.1	asparagine synthetase B	0.955		0.944		0.907		0.994	
TyrB	YP_492197.1	tyrosine aminotransferase, tyrosine-repressible, PLP-dependent	1.037		1.133		0.908		1.072	
DsdA	YP_490608.1	D-serine ammonia-lyase	0.714	*	0.646	*	0.977		0.901	
TrpA	YP_489528.1	tryptophan synthase subunit alpha	1.036		0.949		1.1		0.975	

YfdZ	YP_490621.1	aminotransferase, PLP-dependent	1.005		0.757	*	1.249		0.947	*
YcaC	YP_489169.1	hydrolase	1.006		1.181		1.455	*	1.787	
YgeX	YP_491073.1	2,3-diaminopropionate ammonia-lyase	0.535		0.8	*	0.904		0.924	*
PoxB	YP_489144.1	pyruvate dehydrogenase (pyruvate oxidase), thiamin-dependent, FAD-binding	0.74	*	1.128	*	1.15	*	1.716	*
YedO	YP_490176.1	D-cysteine desulfhydrase, PLP-dependent	1.001		1.076		1.33	*	1.391	*
MppA	YP_489599.1	murein tripeptide (L-ala-gamma-D-glutamyl-meso-DAP) transporter subunit	1.404	*	1.026		1.09		0.757	
EntB_1	YP_488884.1	isochorismatase	2.961	*	2.203	*	1.107		0.932	
IlvB	YP_491763.1	acetolactate synthase I, large subunit	0.754	*	0.536	*	1.245	*	0.972	

Note: [a] 1-vs-2, 12 h with 0 mM versus 1 mM Mn (II); 3-vs-4, 48 h with 0 mM versus 1 mM Mn (II); 1-vs-3, 12 h versus 48 h with 0 mM Mn (II); and 2-vs-4, 12 h versus 48 h with 1 mM Mn (II). Red value denotes up-regulated expression (>1.2 fold); Green value denotes down-regulated (< 0.833 fold).

[b] Sig, significance.

[c] *, *p* -values < 0.05 and the up-regulated value >1.2 fold / down-regulated value < 0.833 fold.

Table S8. The two-component regulatory system related proteins.[a–c]

Protein or Domain	Accession	Desription	1-vs-2	Sig	3-vs-4	Sig	1-vs-3	Sig	2-vs-4	Sig
ArcA	YP_492531.1	DNA-binding response regulator in two-component regulatory system with ArcB or CpxA	1.114		1.034		0.962		0.978	
NarL	YP_489491.1	DNA-binding response regulator in two-component regulatory system with NarX (or NarQ)	0.85		0.957		0.893		0.884	
ArcB	YP_491395.1	hybrid sensory histidine kinase in two-component regulatory system with ArcA	1.009		0.768	*	1.147	*	0.884	
RcsD	YP_490454.1	phosphotransfer intermediate protein in two-component regulatory system with RcsBC	0.993		1.034		1.127		1.132	
UvrY	YP_490171.1	DNA-binding response regulator in two-component regulatory system with BarA	0.968		0.822		1.027		0.737	*
GlnL	YP_491581.1	sensory kinase in two-component regulatory system with GlnG	1.06		0.687		1.124		0.789	
Spy	YP_490004.1	envelope stress induced periplasmic protein	1.174	*	1.614	*	0.969		1.501	*
CpxA	YP_491539.1	sensory histidine kinase in two-component regulatory system with CpxR	1.005		1.143		0.993		0.903	
QseC	YP_491218.1	sensory histidine kinase in two-component regulatory system with QseB	0.879		0.899		1.129		1.24	*
QseB	YP_491217.1	DNA-binding response regulator in two-component regulatory system with QseC	1.106		1.558		0.88		1.227	*
BarA	YP_490994.1	hybrid sensory histidine kinase, in two-component regulatory system with UvrY	1.383		1.433	*	0.977		1.002	

Note: [a] 1-vs-2, 12 h with 0 mM versus 1 mM Mn (II); 3-vs-4, 48 h with 0 mM versus 1 mM Mn (II); 1-vs-3, 12 h versus 48 h with 0 mM Mn (II); and 2-vs-4, 12 h versus 48 h with 1 mM Mn (II). Red value denotes up-regulated expression (>1.2 fold); Green value denotes down-regulated (< 0.833 fold).

[b] Sig, significance.

[c] *, p -values < 0.05 and the up-regulated value >1.2 fold / down-regulated value < 0.833 fold.

Table S9. The stress response-related proteins.[a–c]

Proteins	Accession	Desription	1-vs-2 [a]	Sig [b]	3-vs-4	Sig	1-vs-3	Sig	2-vs-4	Sig
YihK	YP_491579.1	GTP-binding protein	1.035		1.085	* [c]	1		1.032	
YbdQ	YP_488897.1	universal stress protein UP12	0.764		0.858	*	0.943		1.121	
YdaA	YP_489603.1	stress-induced protein	0.901		1.202		0.888	*	1.148	
TrxA	YP_491658.1	thioredoxin 1	1.254		1.124	*	1.047		0.993	
YicC	YP_491790.1	hypothetical protein Y75_p3530	0.886	*	0.958		1.014		1.113	
UspA	YP_491940.1	universal stress global response regulator	0.977		0.997		1.131	*	1.181	*
CspC	YP_490084.1	stress protein, member of the CspA-family	1.765		1.235	*	0.78	*	0.536	*
RplY	YP_490425.1	50S ribosomal protein L25	0.93		1.101		0.757		0.886	
OsmC	YP_489747.1	osmotically inducible, stress-inducible membrane protein	0.904		1.119	*	1.642	*	1.847	
YnaF	YP_489644.1	stress-induced protein, ATP-binding protein	1.275		1.216		1.115	*	1.016	
YjbJ	YP_492188.1	stress response protein	1.199		0.8	*	2.792		1.914	*
CyaY	YP_491635.1	frataxin, iron-binding and oxidizing protein	0.943		0.824		1.148		0.986	
YiiT	YP_491528.1	stress-induced protein	0.85	*	1.216	*	0.652	*	0.991	
BolA	YP_488727.1	regulator of penicillin binding proteins and beta lactamase transcription (morphogene)	0.897		0.755		2.542	*	1.843	
Spy	YP_490004.1	envelope stress induced periplasmic protein	1.174	*	1.614	*	0.969		1.501	*

Note: [a] 1-vs-2, 12 h with 0 mM versus 1 mM Mn (II); 3-vs-4, 48 h with 0 mM versus 1 mM Mn (II); 1-vs-3, 12 h versus 48 h with 0 mM Mn (II); and 2-vs-4, 12 h versus 48 h with 1 mM Mn (II). Red value denotes up-regulated expression (>1.2 fold); Green value denotes down-regulated (< 0.833 fold).

[b] Sig, significance.

[c] *, p -values < 0.05 and the up-regulated value >1.2 fold / down-regulated value < 0.833 fold.

Table S10. The genes selected for RT-qPCR analysis of transcriptional activity with and without Mn(II) induction.

Gene	Protein_ID (GenBank)	Size (bp)	Predicted function	Reference
fliS	AP_002540.1	137	Flagellar protein potentiates	[1]
fliC	AP_002538.1	118	Fagellar filament structural protein	[2]
flhA	AP_002499.1	113	Flagellar biosynthesis pathway, component	[3]
fliG	AP_002551.1	118	Flagellar motor switching and energizing component	[4]
fliE	AP_001709.1	139	Flagellar basal-body component	[5]
mco266	AP_000784.1	123	Multicopper oxidase	[6]
katE	AP_002351.1	168	Hydroperoxidase HPII(III)	[7]
katG	AP_003867.1	113	Catalase/hydroperoxidase HPI(I)	[8]
motA	AP_002510.1	86	Proton conductor component of flagella motor	[9]
motB	AP_002509.1	126	Protein that enables flagellar motor rotation	[9]
sdhC	AP_001359.1	114	Succinate dehydrogenase, membrane subunit, binds cytochrome b556	[10]
sdhB	AP_001362.1	113	Succinate dehydrogenase, FeS subunit	[11]
icd	AP_001762.1	92	Isocitrate dehydrogenase	[12]
aceK	AP_004517.1	150	Isocitrate dehydrogenase kinase/phosphatase	[13]
ccmG	AP_002791.1	148	periplasmic thioredoxin of cytochrome c	[14]
ccmE	AP_002793.1	149	periplasmic heme chaperone	[15]
napC	AP_002798.1	95	nitrate reductase, cytochrome *c*-type,periplasmic	[16]
sodA	AP_003900.1	183	superoxide dismutase, Mn	[17]
sodC	AP_002268.1	183	superoxide dismutase, Cu, Zn	[17]
tynA	AP009048.1	91	Cu^{2+}-containing amine oxidase	[18]

References:

1 Minamino, T. & Namba, K. Self-assembly and type III protein export of the bacterial flagellum. *J Mol Microbiol Biotechnol* **7**, 5−17 (2004).

2 Majander, K., Korhonen, T. K. & Westerlund-Wikstrom, B. Simultaneous display of multiple foreign peptides in the FliD capping and FliC filament proteins of the *Escherichia coli* flagellum. *Appl Environ Microbiol* **71**, 4263−4268 (2005).

3 Bange, G. *et al.* FlhA provides the adaptor for coordinated delivery of late flagella building blocks to the type III secretion system. *Proc Natl Acad Sci USA* **107**, 11295−11300 (2010).

4 Ge, Y., Old, I. G., Girons, I. S. & Charon, N. W. The flgK motility operon of *Borrelia burgdorferi* is initiated by a sigma 70-like promoter. *Microbiology* **143**, 1681−1690 (1997).

5 Paul, K., Brunstetter, D., Titen, S. & Blair, D. F. A molecular mechanism of direction switching in the flagellar motor of *Escherichia coli*. *Proc Natl Acad Sci USA* **108**, 17171−17176 (2011).

6 Singh, S. K. *et al.* Crystal structures of multicopper oxidase CueO bound to copper(I) and silver(I): functional role of a methionine-rich sequence. *J Biol Chem* **286**, 37849−37857 (2011).

7 Jha, V., Chelikani, P., Carpena, X., Fita, I. & Loewen, P. C. Influence of main channel structure on H_2O_2 access to the heme cavity of catalase KatE of *Escherichia coli*. *Arch Biochem Biophys* **526**, 54−59 (2012).

8 Kim, Y. H. & Yu, M. H. Overexpression of reactive cysteine-containing 2-nitrobenzoate nitroreductase (NbaA) and its mutants alters the sensitivity of *Escherichia coli* to reactive oxygen species by reprogramming a regulatory network of disulfide-bonded proteins. *J Proteome Res* **11**, 3219−3230 (2012).

9 Hosking, E. R., Vogt, C., Bakker, E. P. & Manson, M. D. The *Escherichia coli* MotAB proton channel unplugged. *J Mol Biol* **364**, 921−937 (2006).

10 Cheng, V. W., Johnson, A., Rothery, R. A. & Weiner, J. H. Alternative sites for proton entry from the cytoplasm to the quinone binding site in *Escherichia coli* succinate dehydrogenase. *Biochemistry* **47**, 9107−9116 (2008).

11 Wang, B. *et al.* Biochemical properties and physiological roles of NADP-dependent malic enzyme in *Escherichia coli*. *J Microbiol* **49**, 797−802 (2011).

12 Paul, K., Nieto, V., Carlquist, W. C., Blair, D. F. & Harshey, R. M. The c-di-GMP binding protein YcgR controls flagellar motor direction and speed to affect chemotaxis by a "backstop brake" mechanism. *Mol Cell* **38**, 128−139 (2010).

13 Renilla, S. *et al.* Acetate scavenging activity in *Escherichia coli*: interplay of acetyl-CoA synthetase and the PEP-glyoxylate cycle in chemostat cultures. *Appl Microbiol Biotechnol* **93**, 2109–2124 (2012).

14 Ferguson, S. J., Stevens, J. M., Allen, J. W. & Robertson, I. B. Cytochrome c assembly: a tale of ever increasing variation and mystery? *Biochim Biophys Acta* **1777**, 980–984 (2008).

15 Frawley, E. R. & Kranz, R. G. CcsBA is a cytochrome c synthetase that also functions in heme transport. *Proc Natl Acad Sci USA* **106**, 10201–10206 (2009).

16 Shi, L. *et al.* Molecular underpinnings of Fe(III) oxide reduction by *Shewanella oneidensis* MR-1. *Front Microbiol* **3**, 50 (2012).

17 Sanjay, M. K. *et al.* Copper, Zinc-superoxide dismutase from clinically isolated *Escherichia coli*: cloning, analysis of *sodC* and its possible role in pathogenicity. *Indian J Microbiol* **51**, 326–331 (2011).

18 Rankin, L. D. *et al.* *Escherichia coli* NsrR regulates a pathway for the oxidation of 3-nitrotyramine to 4-hydroxy-3-nitrophenylacetate. *J Bacteriol* **190**, 6170–6177 (2008).

Table S11. Heat shock proteins and cold shock proteins identified by iTRAQ.[a–c]

Protein or Domain	Accession	Desription	1-vs-2 [a]	Sig [b]	3-vs-4	Sig	1-vs-3	Sig	2-vs-4	Sig
**Heat shock proteins**										
GroL	YP_492286.1	Cpn60 chaperonin GroEL, large subunit of GroESL	1.113		1.129		1.127		1.141	
HtpG	YP_488764.1	molecular chaperone HSP90 family	0.898	* [c]	1.011		0.974		1.095	
MreB	YP_491434.1	cell wall structural complex MreBCD, actin-like component	1.173		0.952		1.274		1.079	
GrpE	YP_490836.1	heat shock protein	0.958		0.908		1.103		1.022	
HscA	YP_490754.1	DnaK-like molecular chaperone-specific for IscU	1.054		1.009		1.101	*	1.11	*
HslO	YP_492031.1	heat shock protein Hsp33	1.01		0.904		1.332	*	1.062	
YrfH	YP_492032.1	ribosome-associated heat shock protein Hsp15	---		---		---		---	
IbpA	YP_491748.1	heat shock chaperone	0.956		0.881		1.092		0.996	
IbpB	YP_491749.1	heat shock chaperone	---		---		---		---	
**Cold shock proteins**										
CspC	YP_490084.1	stress protein, member of the CspA-family	1.765		1.235	*	0.78	*	0.536	*
CspA	YP_491880.1	major cold shock protein	1.567	*	0.915		0.746	*	0.396	*
CspE	YP_488914.1	DNA-binding transcriptional repressor	1.189	*	1.384	*	0.642	*	0.678	*
YfiA	YP_490820.1	cold shock protein associated with 30S ribosomal subunit	1.208		1.025		0.636		0.742	
CspD	YP_489153.1	cold shock protein	3.245	*	0.627		0.961		0.184	*

Note: [a] 1-vs-2, 12 h with 0 mM versus 1 mM Mn (II); 3-vs-4, 48 h with 0 mM versus 1 mM Mn (II); 1-vs-3, 12 h versus 48 h with 0 mM Mn (II); and 2-vs-4, 12 h versus 48 h with 1 mM Mn (II). Red value denotes up-regulated expression (>1.2 fold); Green value denotes down-regulated (< 0.833 fold).

[b] Sig, significance.

[c] *, p -values < 0.05 and the up-regulated value >1.2 fold / down-regulated value < 0.833 fold.

Table S12. The CheY-related proteins identified by iTRAQ.[a-c]

Protein or Domain	Accession	Desription	1-VS-2	Sig	3-VS-4	Sig	1-VS-3	Sig	2-VS-4	Sig
ArcA	YP_492531.1	DNA-binding response regulator in two-component regulatory system with ArcB or CpxA	1.114		1.034		0.962		0.978	
NarL	YP_489491.1	DNA-binding response regulator in two-component regulatory system with NarX (or NarQ)	0.85		0.957		0.893		0.884	
PhoP	YP_489398.1	DNA-binding response regulator in two-component regulatory system with PhoQ	0.629	*	0.615	*	1.097		1.022	
OmpR	YP_492027.1	DNA-binding response regulator in two-component regulatory system with EnvZ	0.925		1.189		0.835		0.986	
NarP	YP_490431.1	DNA-binding response regulator in two-component regulatory system with NarQ or NarX	0.908		0.858	*	0.74		0.773	*
EvgA	YP_490611.1	DNA-binding response regulator in two-component regulatory system with EvgS	0.713	*	0.81	*	0.926		1.122	
RcsB	YP_490455.1	DNA-binding response regulator in two-component regulatory system with RcsC and YojN	1.032		1.073		0.803	*	0.887	
GlnG	YP_491582.1	fused DNA-binding response regulator in two-component regulatory system with GlnL, nitrogen regulator I (NRI)	0.965		0.875		0.98		0.87	*
UvrY	YP_490171.1	DNA-binding response regulator in two-component regulatory system with BarA	0.968		0.822		1.027		0.737	*
BaeR	YP_490321.1	DNA-binding response regulator in two-component regulatory system with BaeS	1.023		1.099		0.835		0.962	
CpxR	YP_491538.1	DNA-binding response regulator in two-component regulatory system with CpxA	0.875		1.049		0.724	*	0.879	
QseB	YP_491217.1	DNA-binding response regulator in two-component regulatory system with QseC	1.106		1.558		0.88		1.227	*
YfhA	YP_490782.1	DNA-binding response regulator in two-component system	0.722		0.827	*	1.263		1.243	

Note: [a] 1-vs-2, 12 h with 0 mM versus 1 mM Mn (II); 3-vs-4, 48 h with 0 mM versus 1 mM Mn (II); 1-vs-3, 12 h versus 48 h with 0 mM Mn (II); and 2-vs-4, 12 h versus 48 h with 1 mM Mn (II).

[b] Sig, significance.

[c] *, *p*-values < 0.05 and the up-regulated value >1.2 fold / down-regulated value < 0.833 fold.

Table S13. *E. coli* strains and plasmids used in this study.

Strains or plasmids	Phenotypes	Sources
E. coli strains		
DH5α	s*upE44ΔlacU169(Φ80 lacZΔM15) hdsR17 recA1 endA1 gyrA96 thi-1 relA1*	Lab collection
JM109	*recA1endA1gyrA96thihsdR17supE44relA1Δ(lac-proAB)/F'* [*traD36 proAB+ lacIq lacZΔM15*]	Lab collection
MB266	Wild-type strain with high Mn(II)-oxidizing activity	Lab collection
MB279	MB266-Δ*katE*	This study
MB280	Transformed MB279 harboring pMB262	This study
MB502	MB266-Δ*poxB*	This study
MB503	Transformed *E. coli* MB502 harboring pMB503	This study
MB505	MB266-Δ*sodA*	This study
MB506	Transformed *E. coli* MB505 harboring pMB506	This study
MB507	MB266-Δ*spy*	This study
MB508	Transformed MB507 harboring pMB508	This study
WM3064	*dap* genetically defective strain	[1]
Plasmids		
pTrcHis-B	*E. coli* expression vector, Amp^r; 4412 bp	Invitrogen
pTrcHis-C	*E. coli* expression vector, Amp^r; 4412 bp	Invitrogen
pKD46	Red recombination help plasmid; P_{araB}; Amp^r; exo^+; bet^+; gam^+; 6.3 kb	Lab collection
pKD4	To clone Kan gene with FRT site; Kan^r ; 3267 bp	Lab collection
pCP20	Red recombination; Amp^r; Cm^r; FLP^+; 9.4 kb	Lab collection
pDS3.0	Suicide plasmid, $Sucrose^r$, Gm^r	[1]
pMB262	Amp^r; 2262bp of PCR-amplified *katE* gene cloned to pTrcHis-C at *Bgl*II/ *Kpn*I sites; 6674 bp	This study
pMB506	Amp^r; 620 bp of PCR-amplified *sodA* gene cloned to pTrcHis-B at *Bgl*II/*Eco*RI sites; 5032 bp	This study
pMB508	Amp^r; 485 bp of PCR-amplified *spy* gene cloned to pTrcHis-B at *Bgl*II/ *Kpn*I sites; 4897 bp	This study
pMB503	Amp^r; 1718bp of PCR-amplified *poxB* gene cloned to pTrcHisB at *Bgl*II/ *Kpn*I sites; 6130 bp	This study

References

1 Gao, W. *et al.* Knock-out of SO1377 gene, which encodes the member of a conserved hypothetical bacterial protein family COG2268, results in alteration of iron metabolism, increased spontaneous mutation and hydrogen peroxide sensitivity in *Shewanella oneidensis* MR-1. *BMC Genomics* **7**, 76 (2006).

Table S14. The oligonucleotide primers used for RT-qPCR analyses.

Genes	Primer sequences
16S rRNA gene	27F: 5′–AATGGCGCATACAAAGAGAAGC–3′
	1492R: 5′–GTTGCAGACTCCAATCCGGA–3′
fliS	fliSF: 5′– CACTGGTTAGAGCGAGCCT –3′
	flliSR: 5′– TTGGGTTAGTTCGTCTTTGC –3′
fliC	fliCF: 5′– CCTTTCTACGGAAGCAGC –3′
	fliCR: 5′– CCCATCGTTATCACCACC –3′
flhA	flhAF: 5′– GGAAAGTTATACGCTATTGACC –3′
	flhAR: 5′– CATCCTGATCGGTGCTGA –3′
fliG	fliGF: 5′–GCAGGGCTATCAGGAAGG–3′
	fliGR: 5′–CTATCAAGTGCATCAAGGGT–3′
fliE	flgLF: 5′–GCGAAGCGGATAAAGAAA–3′
	flgLR: 5′–CCTAATGAATCCAGCGACT–3′
mco266	mco266F: 5′–CAATCTACCCGCAACACG–3′
	mco266R: 5′–CGCTGGCAATCACATACA–3′
katE	katEF: 5′–TTGTGGGAAGCCATTGAA–3′
	katER: 5′–GCGATTGAGCACCATTTT–3′
katG	katGF: 5′–TACAACCCGACCGAGCAG–3′
	katGR: 5′–CCACGGAAGGTAGAAGCAG–3′
motA	motAF: 5′–TTATCCCAACCTGTCACTACAT–3′
	motAR: 5′–ATCTTCGCCTTTCTCATTCA–3′
motB	motBF: 5′–TTGCTGTCGTGGTTCCTG–3′
	motBR: 5′–CGCAATCAGCCCTTCATA–3′
sdhC	sdhCF: 5′–GCGATAGCGTCCATTCTC–3′
	sdhCR: 5′–AGCTTGCTCGAAACCTTC–3′
sdhB	sdhBF: 5′–TTCAACCTCTTGTCCGTCTT–3′
	sdhBR: 5′–GGCTGTCAGTCTCGGTATCA–3′
icd	icdF: 5′–GTTACTATCAGGGCACTCCA–3′
	icdR: 5′–ACCCGCATAAATGTCTTC–3′
aceK	aceKF: 5′–CAAGGCTTCGATGCTCAG–3′

	aceKR: 5′−TTGCTCCACGACCAGACC−3′
ccmG	ccmG: 5′−GGCTGGATCTCGGTGTCT −3′
	ccmGR: 5′− GCGGCCTCCTTACTGTATT−3′
ccmE	ccmEF: 5′−ATTGCCTGTGCCGTGTTG −3′
	ccmER:5′−AGACGCTGACCGACTTCC−3′
napC	napCF: 5′− GGGACGCTGCTGTTGATC’ −3′
	napCR: 5′− CGCTACGGTTGTTGTAGTGC−3′
sodA	sodAF: 5′− ATGTGGTCAGTCGGTGGG −3′
	sodAR: 5′− GAGATAACGCATGGTCAGG −3′
sodC	sodCF: 5′− ATGTGGTCAGTCGGTGGG −3′
	SodCR: 5′− GAGATAACGCATGGTCAGG −3′
tynA	tynAF: 5′− AGCGTATCTGGACTCTGGT −3′
	tynAF: 5′− AAGGAGCACTGCGTTAGA−3′

Table S15. The oligonucleotide primers used for gene disruption and complementation.

Primers	Primer sequences
The primers for gene disruption	
K1H1	5′–ATGTCGCAACATAACGAAAAGAACCCACATCAGCACCAGTCACCACTACAGTGTAGGCTGGAGCTGCTTC–3′
K2H2	5′–AGTCCGTCCTTAAAACAGTTAGAATCCTTACGCACTGGTGTGCGCCACACATGGGAATTAGCCATGGTCC–3′
PoxBF1	5′–CGAGCTCATGCGTCACCAGGCGGACAATC–3′ (*Sac*I)
PoxBR1	5′–CATTCAGGAGATGGAGAACCAAAGGGTGGCATTTCCCGTCA–3′
PoxBF2	5′–TGACGGGAAATGCCACCCTTTGGTTCTCCATCTCCTGAATG–3′
PoxBR2	5′–CGAGCTCGTGATTATCTCTGGCTTTCGG–3′ (*Sac*I)
SpyF1	5′–CGAGCTCTAAAAGCCGCAGGGTGTG–3′ (*Sac*I)
SpyR1	5′–CTGAAAGGAAGGATATAGAATTCTTTCAGCCAAAAAACTTAAGACC–3′
SpyF2	5′–GGTCTTAAGTTTTTTGGCTGAAAGAATTCTATATCCTTCCTTTCAG–3′
SpyR2	5′–CGAGCTCGGAACTGACGCCATTAAC–3′ (*Sac*I)
SodAF1	5′–CGAGCTCGCCAGCGTTGAAAGTCCGAA–3′ (*Sac*I)
SodAR1	5′–GACAATACTGGAGATGAATTCATTTGCCGCCTGCTGCAATGAGG–3′
SodAF2	5′–CCTCATTGCAGCAGGCGGCAAATGAATTCATCTCCAGTATTGTC–3′
SodAR2	5′–CGAGCTCCAGCCCTTTTTTCAGATTG–3′ (*Sac*I)
The primers for gene complementation	
poxBCF	5′–GAAGATCTTCGCCTTATGCCCGATGAT–3′ (*Bgl*II)
poxBCR	5′–CGGAATTCGGCATGTCCTTATTATGACGGG–3′ (*Eco*RI)
spyCF	5′–CGC GAGCTC G ATGCGTAAATTAACTGCAC–3′ (*Sac*I)
spyCR	5′–TGC GGTACC TTATTCAGCAGTTGCAGG–3′ (*Kpn*I)
sodACF	5′–GGAAGATCTATGAGCTATACCCTGCCATC–3′ (*Bgl*II)
sodACR	5′–CCGGAATTCCCTCAATGGAGCATCAGT–3′ (*Eco*RI)
katECF	5′–AGGAGATCTATGTCGCAACATAACGAA–3′ (*Bgl*II)
katECR	5′–GACCTGCAGTCAGGCAGGAATTTTGTC–3′ (*Pst*I)

Supplementary Figures

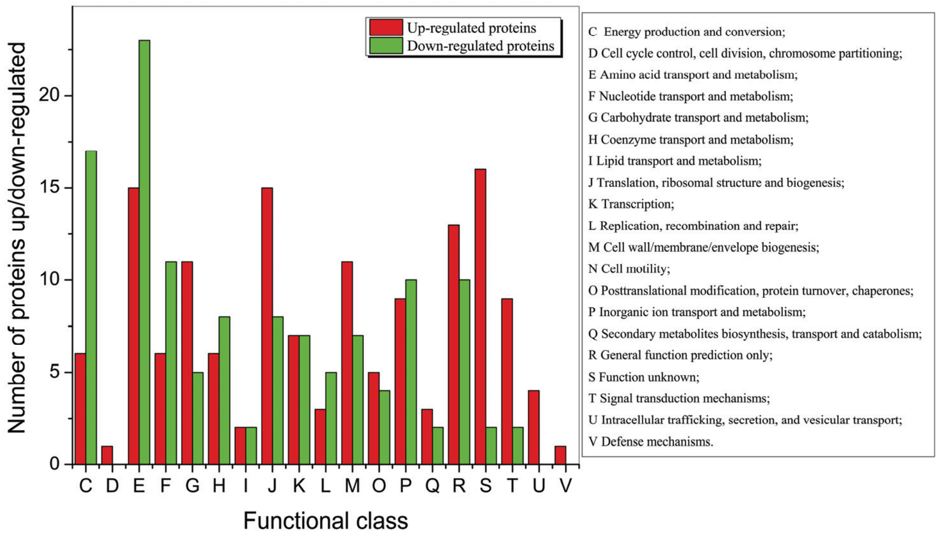

Figure S1. Functional classification of differential expressed proteins in the pairwise comparison 1-*vs*-2. In total 244 proteins were classified into 21 different categories based on their predicted functions. Among them, 135 proteins were found to be up-regulated and 109 proteins were down-regulated.

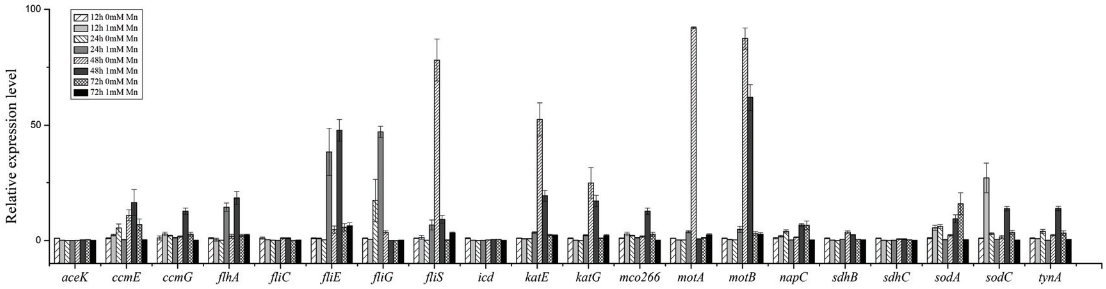

Figure S2. Real time quantitative PCR analysis of the transcriptional activities of 20 selected genes. Total RNA was isolated from strain MB266 cultured for 12 h, 24 h, 48 h and 72 h with or without 1 mM Mn(II) in Lept medium. The 16S rRNA gene was used as a reference.

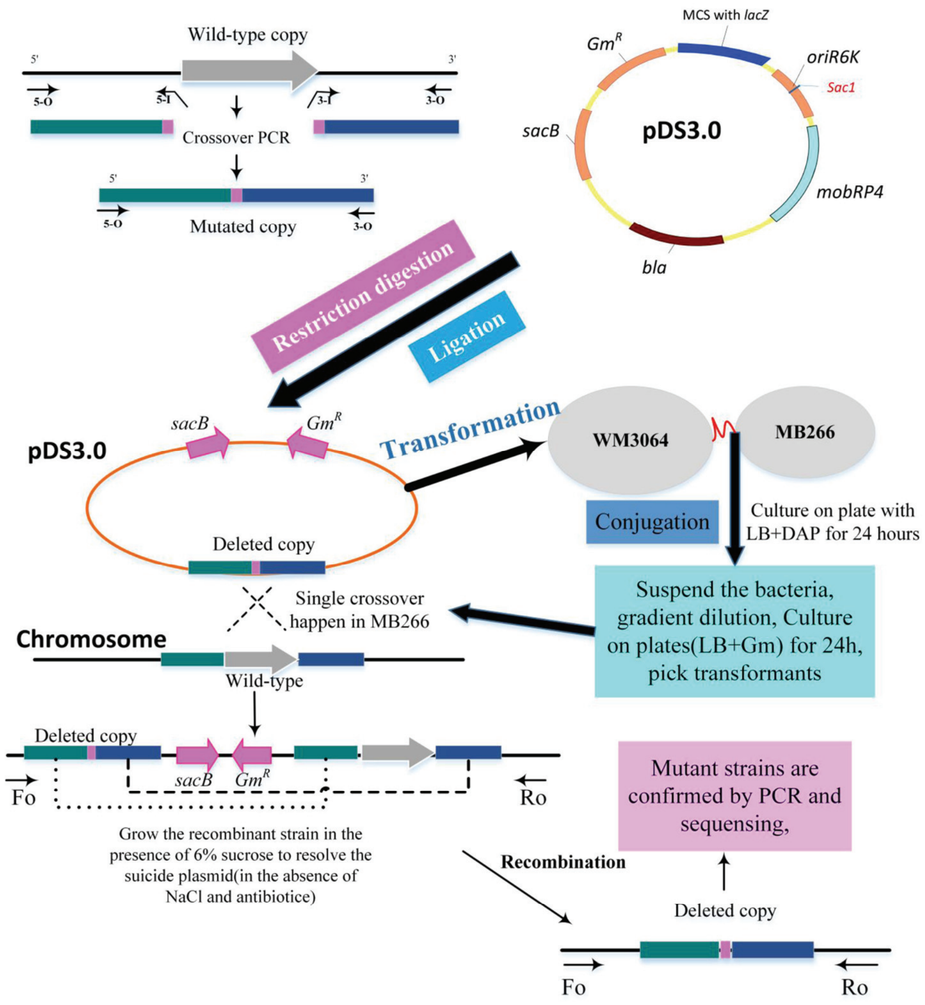

Figure S3. **Schematic illustration of in-frame gene disruption strategy for *E. coli* MB266 target genes using the suicide vector pDS3.0 recombinant system.**
